# Supplementary material for: Statistically unbiased prediction enables accurate denoising of voltage imaging data
Source: Nat Methods. 2023 Sep 18;20(10):1581–92. doi: 10.1038/s41592-023-02005-8 (PMC10555843; doi:10.1038/s41592-023-02005-8)
Supplement: Supplementary file 1 — Supplementary Figs. 1–65, Videos 1–9 and Tables 1 and 2. [file 41592_2023_2005_MOESM1_ESM.pdf]

nature methods

Article https://doi.org/10.1038/s41592-023-02005-8

Statistically unbiased prediction enables accurate denoising of voltage imaging data

In the format provided by the authors and unedited

SUPPLEMENTARY INFORMATION

Supplementary Figures 1–65

Supplementary Videos 1–9

Supplementary Tables 1-2

References

SUPPLEMENTARY FIGURES

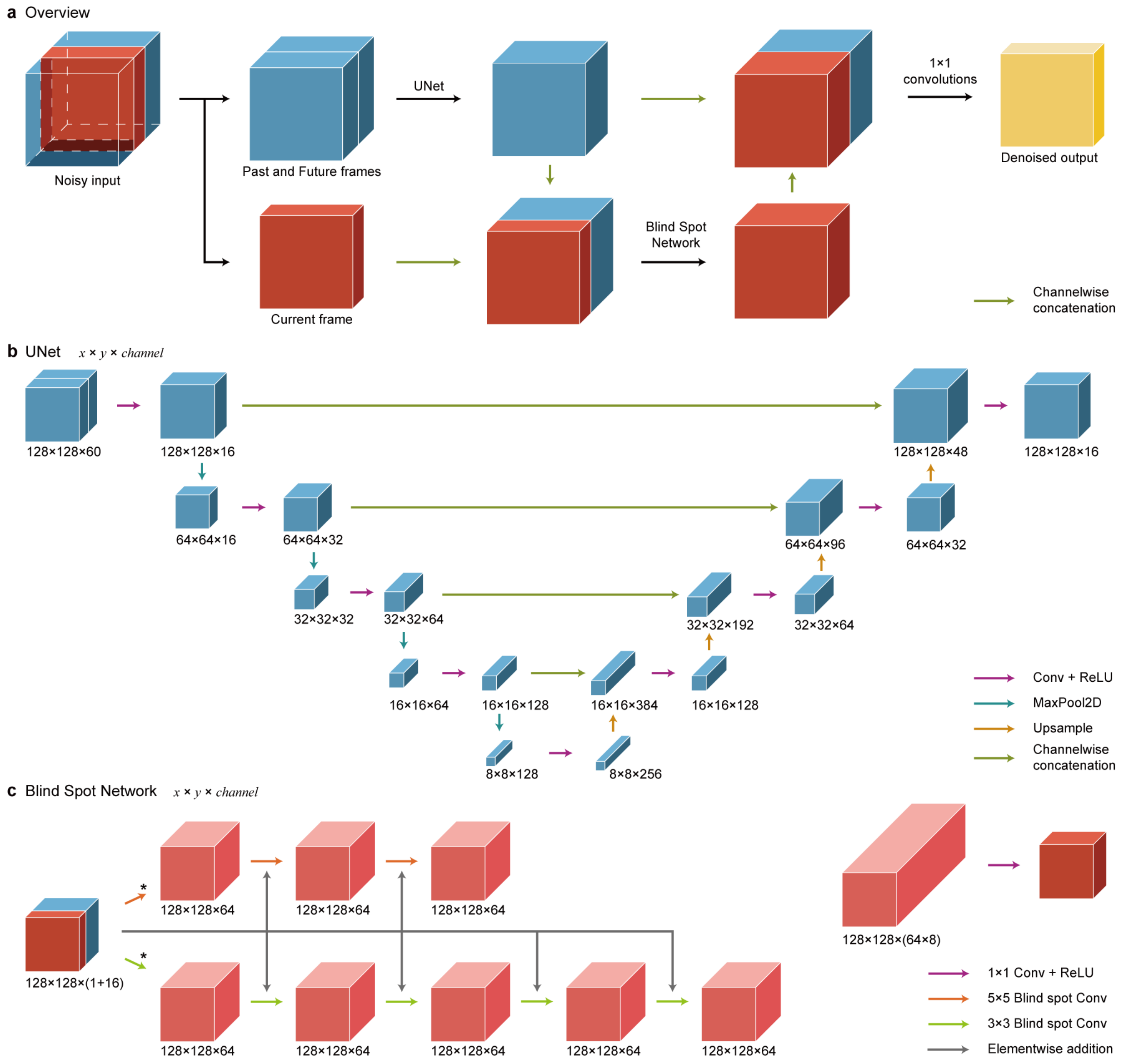

Supplementary Fig. 1: Network architecture. a, Overall architecture of the network. The network predicts the center value based on its spatiotemporally neighboring pixels. Pixels in past and future frames are processed by 2D U-Net, and spatially neighboring pixels in the current frame are processed by a blind spot network. **b**, 2D U-Net, which consists of a 2D encoder, a 2D decoder, and skip connections, processes the past and future frames. The past and future frames are channelwise concatenated and given to the 2D U-Net as input. **c**, The blind spot network consists of blind spot convolution layers, which are convolution layers with zero at the center of the kernels. In addition to the current frame, feature maps from the 2D U-Net, which encode the information in temporally neighboring frames, are channelwise concatenated and given as input to the blind spot network. (*) The first blind spot convolution layer has a blind spot property only in the first channel, which corresponds to the current frame. For the other layers, there are blind spots in all channels.

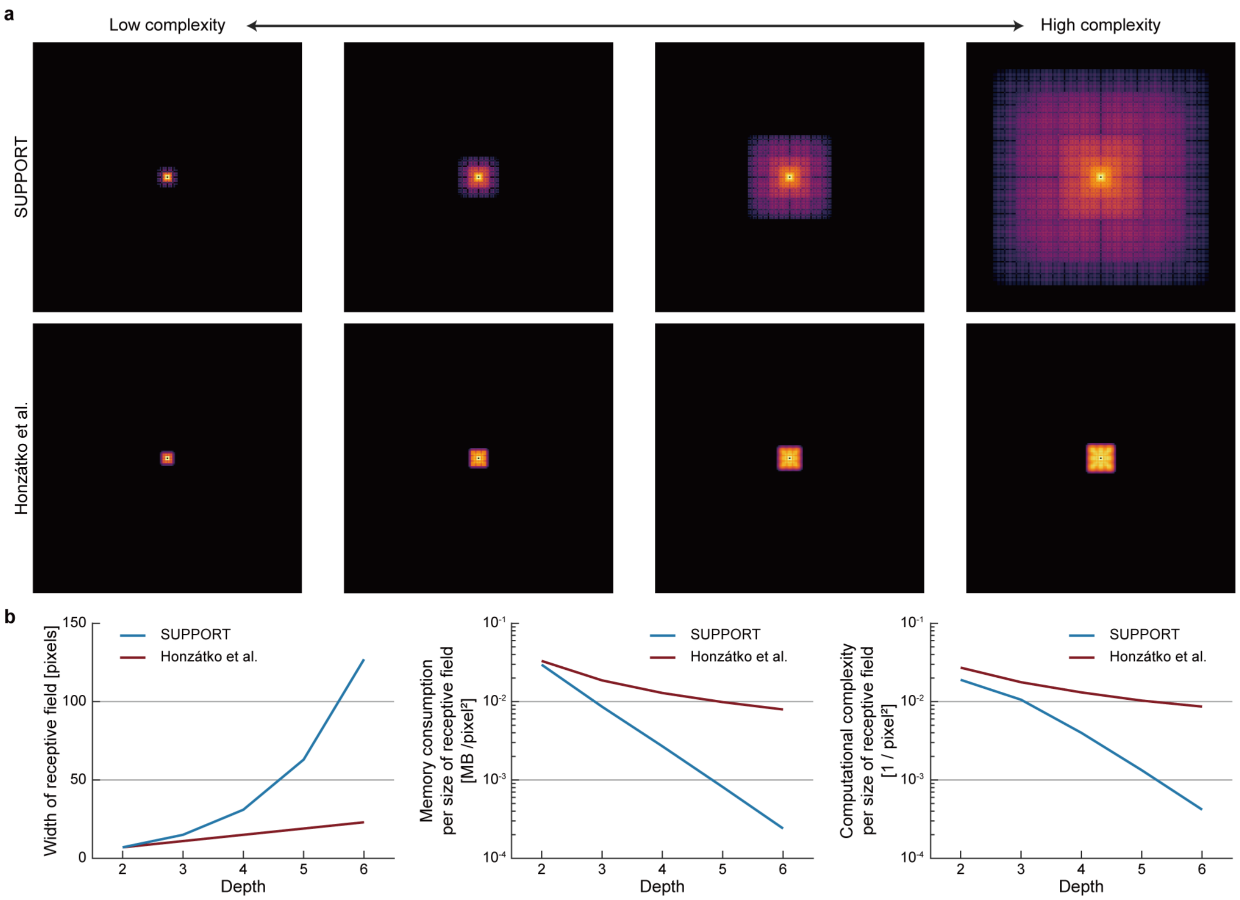

Supplementary Fig. 2: Comparison of blind spot networks with different complexities. a, Impulse response of two blind spot network architectures (SUPPORT, Honzátko et al.[1]) with different network complexities. While the width of SUPPORT's receptive field increases exponentially, Honzátko et al. increases linearly. **b**, The width of the receptive field, memory consumption, and the number of multiply-add operations over the size of the receptive field of SUPPORT and Honzátko et al. for different depths.

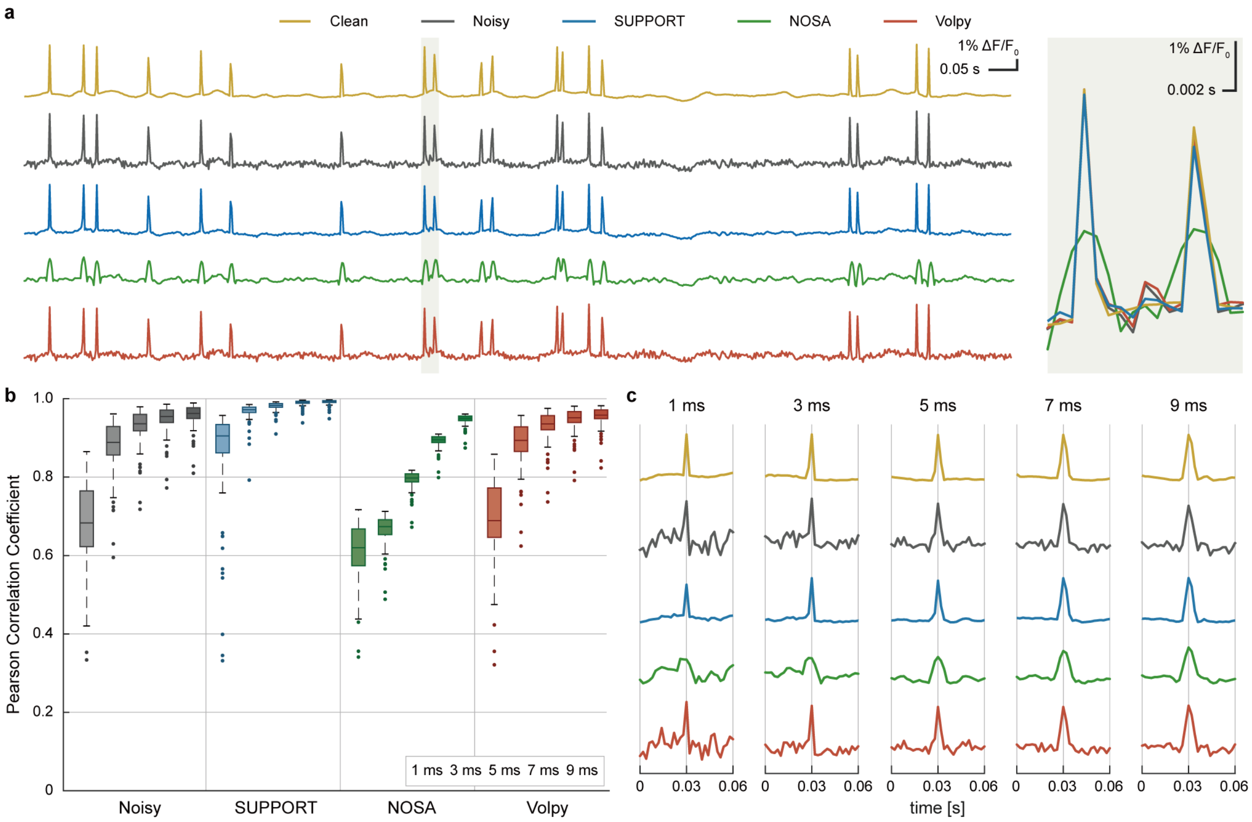

Supplementary Fig. 3: SUPPORT performance comparison with other voltage imaging data analysis algorithms on simulated data. a, Traces from clean, noisy, SUPPORT-denoised, NOSA-denoised, and Volpy-denoised data. Enlarged view of the gray region in **a** is displayed on the right. **b**, Box-and-whisker plot showing Pearson correlation coefficients before and after denoising for different spike widths. N=116 for each test, which represents the number of neurons. **c**, Single cell fluorescence traces near spiking events. From top to bottom: Clean, noisy, SUPPORT-denoised, NOSA-denoised, and Volpy-denoised data. From left to right: spike widths of 1 ms, 3 ms, 5 ms, 7 ms, and 9 ms.

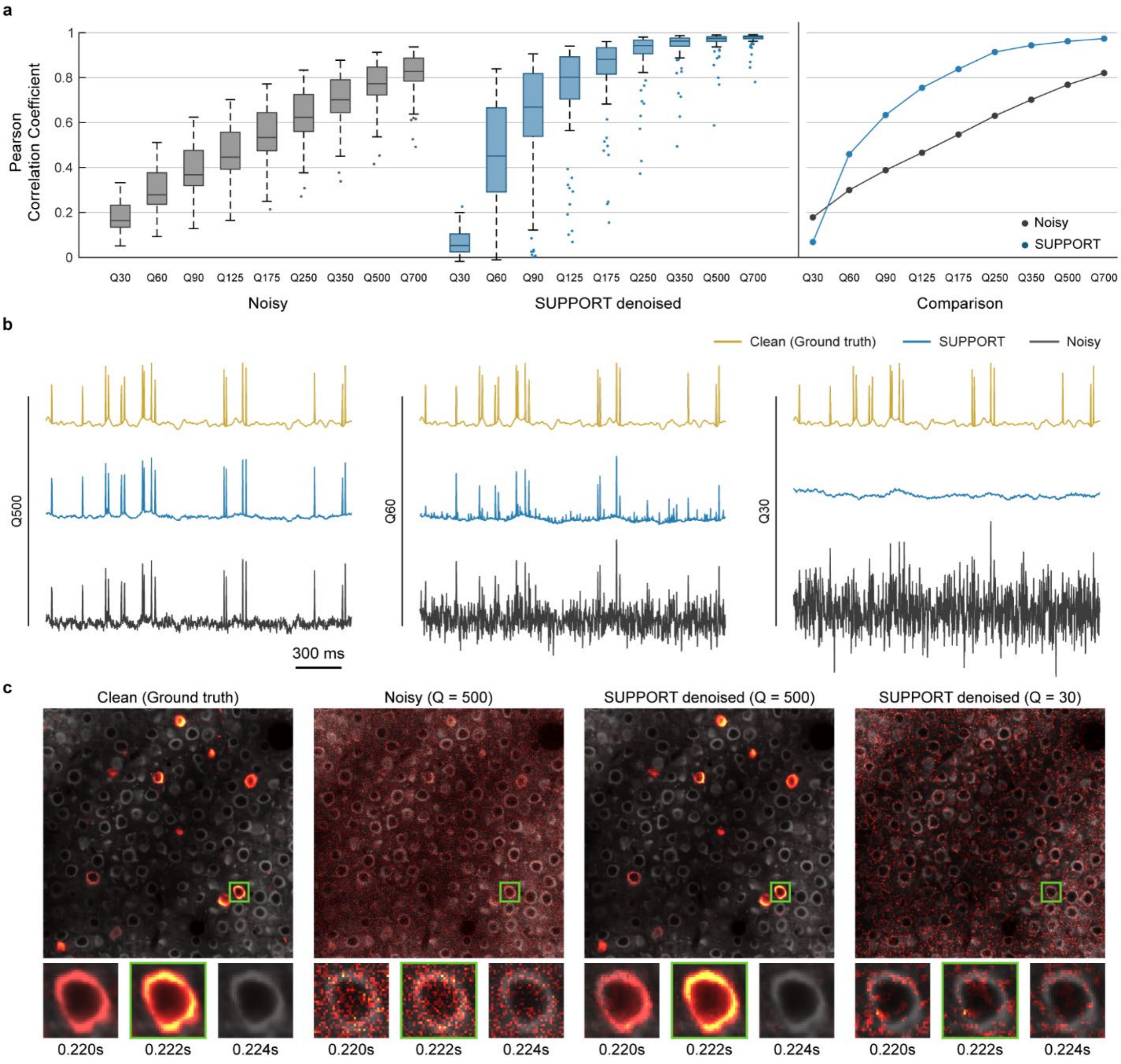

Supplementary Fig. 4: SUPPORT denoising of simulated images with various noise levels. a, Left: Box-and-whisker plot showing Pearson correlation coefficients before and after denoising data with the different noise levels. Right: Line chart showing average Pearson correlation coefficients before and after denoising data with the different noise levels. Q30 indicates each pixel captured 30 photons in maximum and contained corresponding level of Poisson noise. N=116 for each test, which represents the number of neurons. **b**, Fluorescence traces extracted by averaging the ROI of the image. From left to right: noise level of Q500, Q60, and Q30. From top to bottom: Ground truth, SUPPORT denoised data, and noisy data. **c**, Representative frames of clean, noisy (Q500) and SUPPORT denoised data (Q500 and Q30). Baseline and activity components are decomposed from the data. The baseline component with a gray colormap and activity component with a hot colormap are overlaid. Magnified views of the boxed regions are presented underneath with the consecutive frames of the spiking event (t=0.222s).

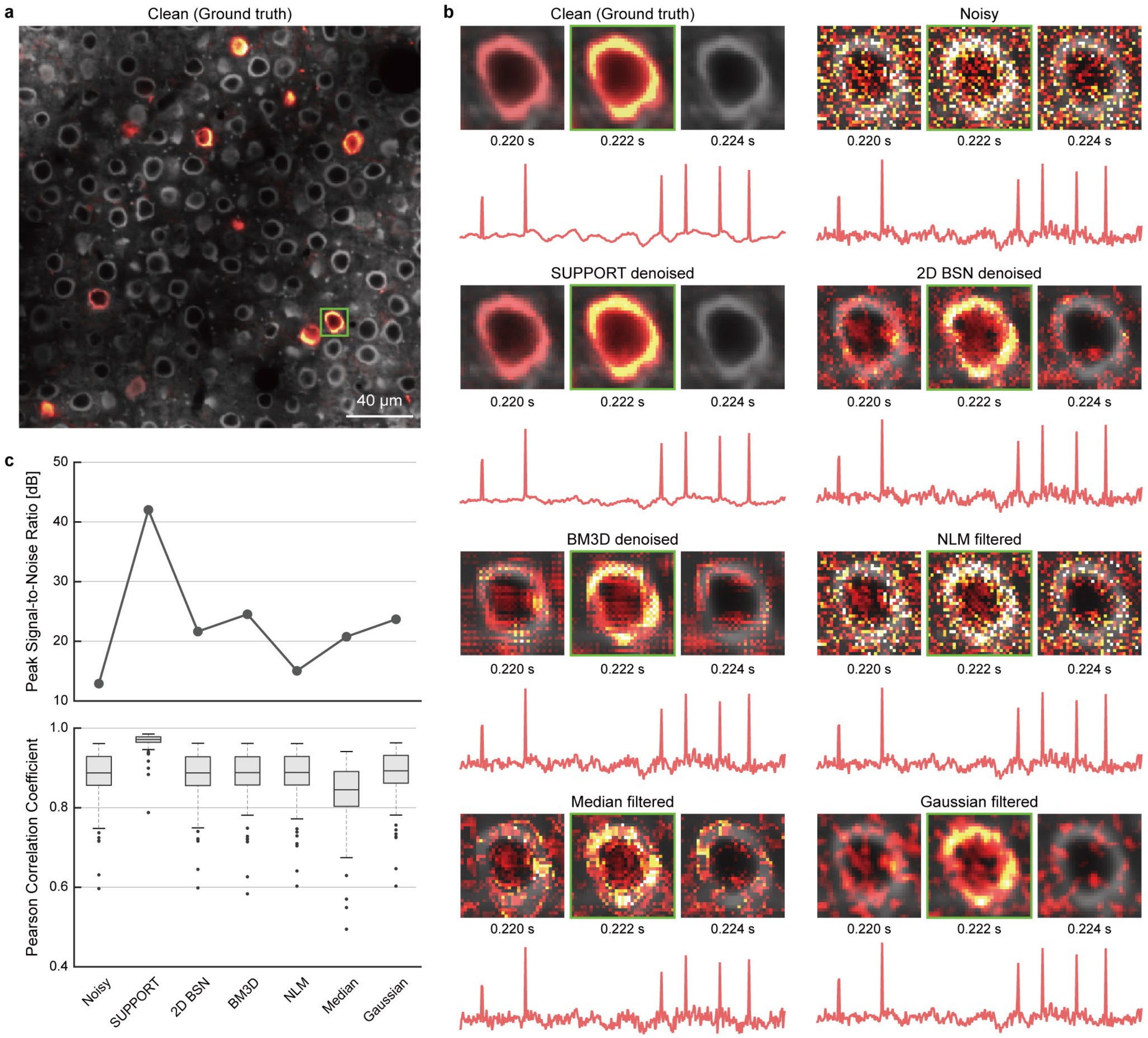

Supplementary Fig. 5: Denoising performance compared with other 2D denoising methods on simulated data. a, Clean synthetic population voltage imaging data (t=0.222 s). Baseline and activity components are decomposed from the data. The baseline component with a gray colormap and activity component with a hot colormap are overlaid. **b**, Consecutive frames of a green box in **a** from clean, noisy, SUPPORT, 2D BSN, BM3D denoised, NLM, median and gaussian filtered data. Traces are plotted below. **c**, PSNR and Pearson correlation coefficients of the baseline-corrected data before and after denoising. N=116 for each test, which represents the number of neurons.

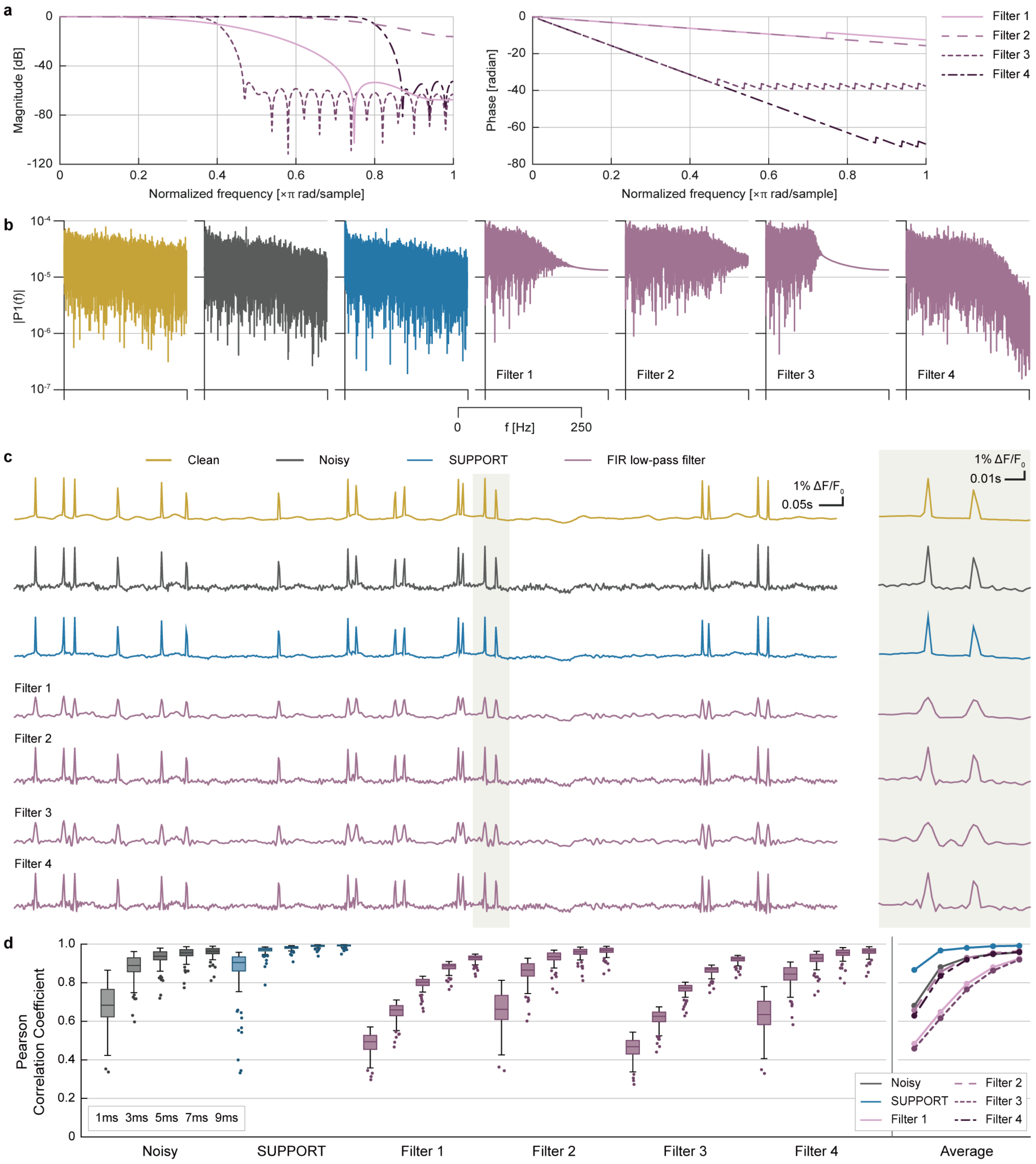

Supplementary Fig. 6: Comparison between SUPPORT and low-pass FIR filtering on simulated voltage imaging data. a, Frequency responses of FIR filters with different filter order and cutoff frequencies. Filter specifications are (filter order, cutoff frequency): Filter 1 = (10, 100Hz), Filter 2 = (10, 200Hz), Filter 3 = (50, 100Hz), Filter 4 = (50, 200Hz). **b**, Single-sided amplitude spectrum of traces. Voltage traces with 3ms spike width are shown. **c**, Traces used in **b**. Magnified view is presented on the right side. **d**, Left: Box-and-whisker plot showing Pearson correlation coefficients before and after denoising data with different spike widths. Right: Line chart showing average Pearson correlation coefficients before and after

denoising data with different spike widths, N = 116 for each test, which represents the number of neurons.

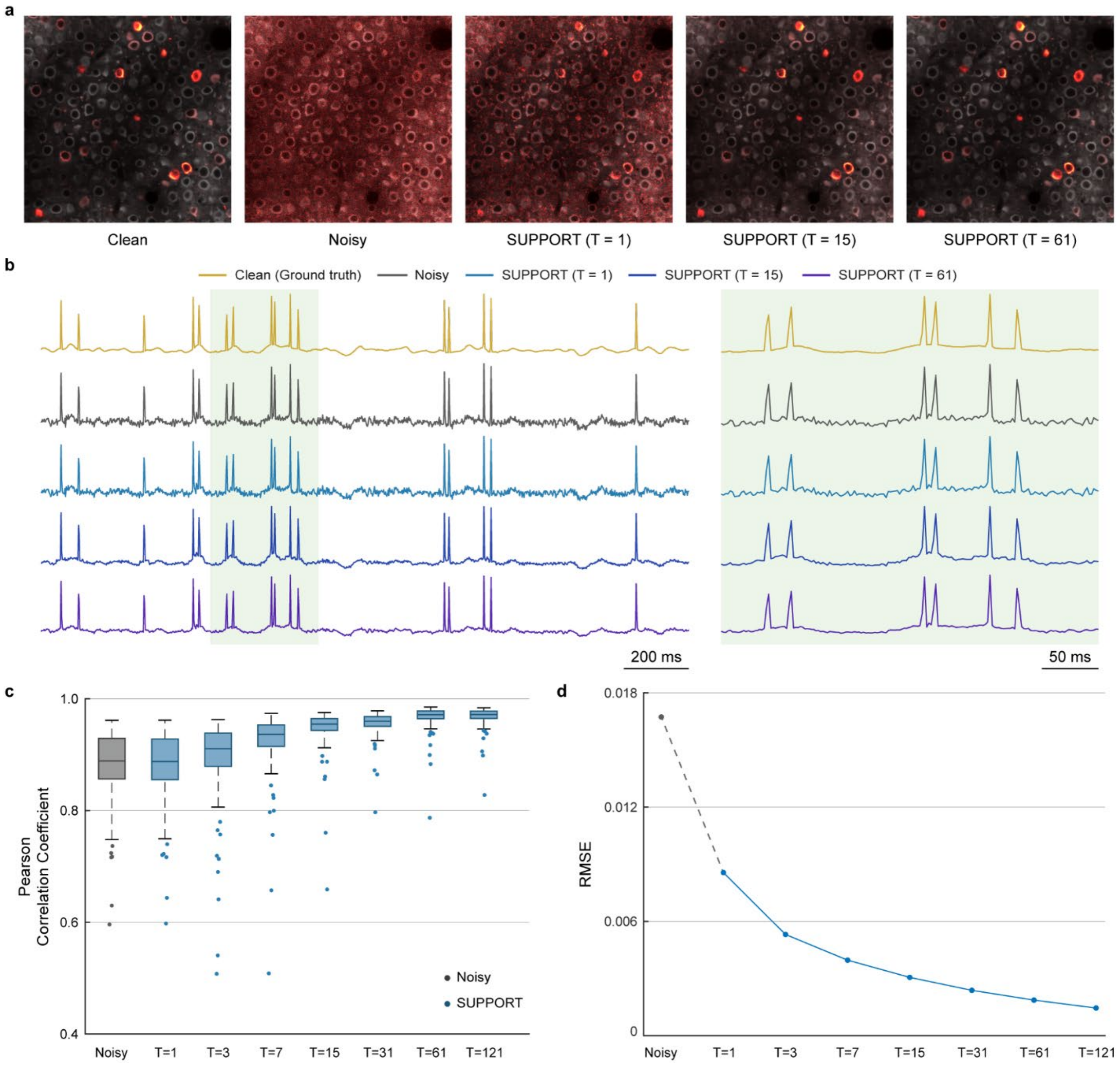

Supplementary Fig. 7: Comparison of denoising performance of SUPPORT with varying temporal receptive field sizes. a, Representative frames of a clean video, a noisy video, and SUPPORT-denoised videos using different receptive fields after baseline correction. Simulated data with spike width of 3ms was used. Receptive fields of 1, 15, and 61 in the temporal direction were used. **b**, Traces extracted from a single cell in the clean video, the noisy video, and the SUPPORT-denoised videos. Temporally expanded traces from the green area on the left are shown on the right. **c**, Box-and-whisker plot showing Pearson correlation coefficients from the extracted traces before and after denoising data with different receptive fields. N=116 for each test, which represents the number of neurons. **d**, Line plot showing RMSE before and after denoising data with different receptive fields.

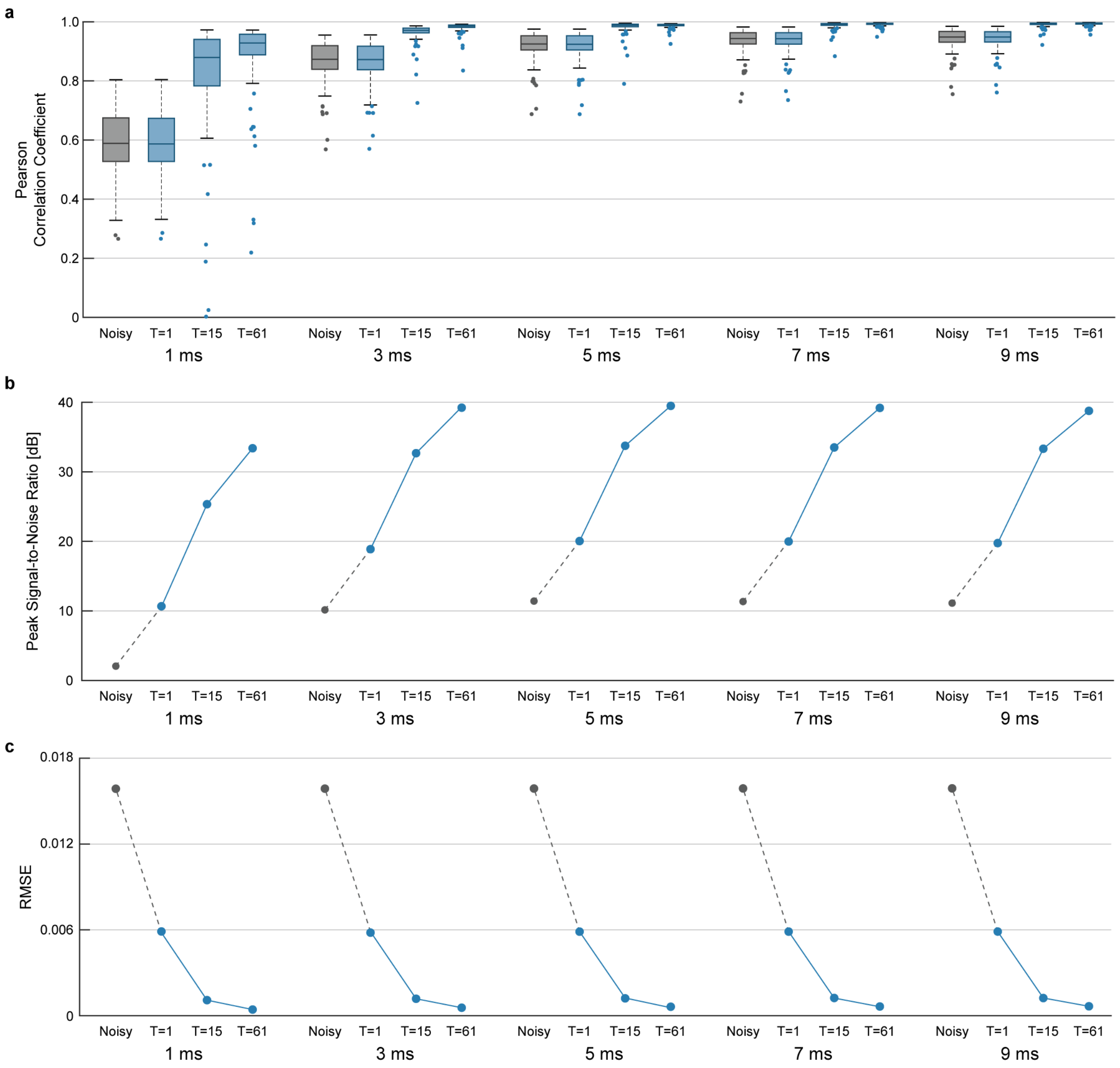

Supplementary Fig. 8: Denoising performance of SUPPORT on simulation data with various spike widths and receptive fields. a, Box-and-whisker plot showing Pearson correlation coefficients before and after denoising data with different receptive fields in the temporal direction. Simulated data with changing spike widths of 1ms, 3ms, 5ms, 7ms, and 9ms were used. N=116 for each test, which represents the number of neurons. **b**, Line plot showing peak signal-to-noise ratio before and after denoising data with different receptive fields in the temporal direction. **c**, Line plot showing RMSE before and after denoising data with different receptive fields in the temporal direction.

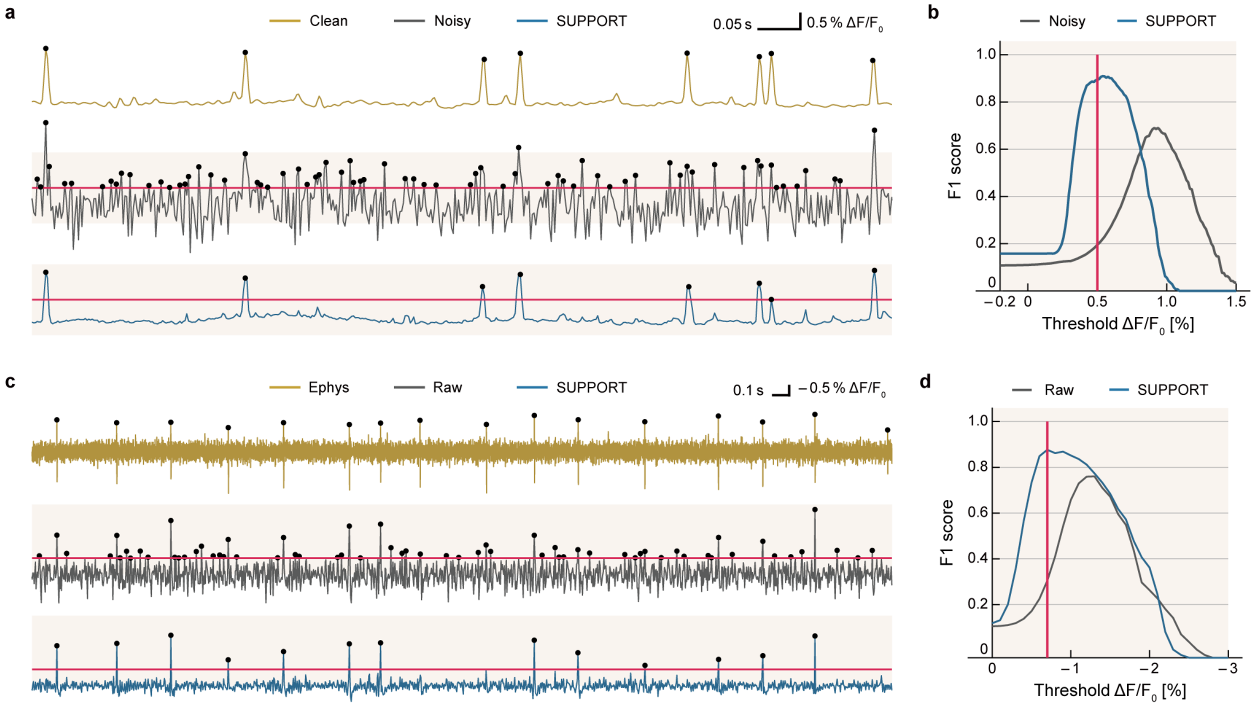

Supplementary Fig. 9: SUPPORT enhances spike detection accuracy. **a**, Traces from clean, noisy, and SUPPORT-denoised data tested with synthetic population voltage imaging data. Spikes are detected by thresholding the clean trace and finding local maximum locations. Red horizontal lines indicate the example threshold within the threshold region colored in pink. **b**, F1 scores across dF/F0 threshold values used for spike detection. Red vertical line and pink region correspond to those in **a**. **c**, Electrophysiological recording and traces from voltage imaging data before and after SUPPORT denoising. Spikes are detected by thresholding the electrophysiological recording and finding local maximum locations. **d**, F1 scores across dF/F0 threshold values used for spike detection.

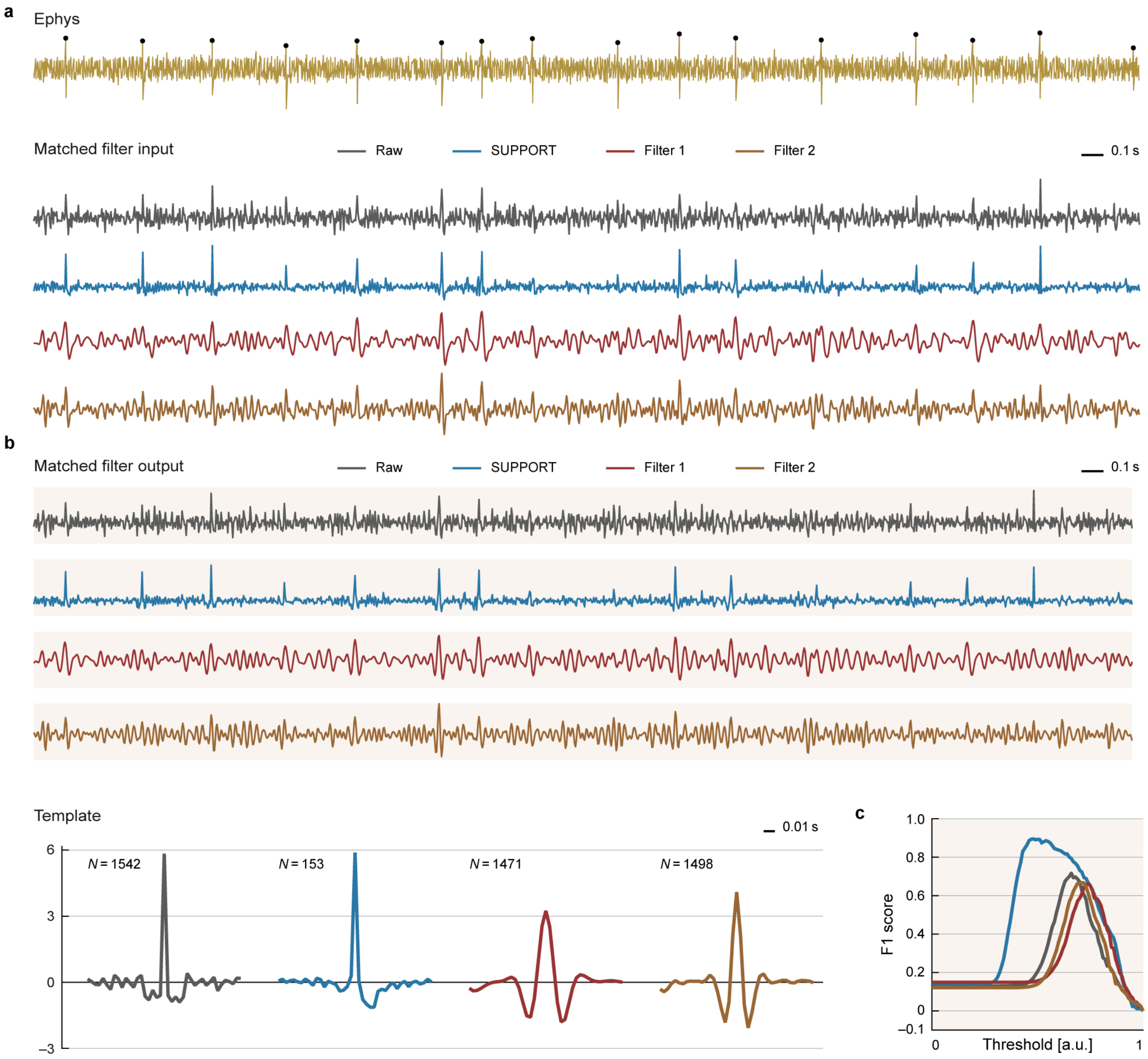

Supplementary Fig. 10: Comparison between SUPPORT and low-pass FIR filtering on single-neuron voltage imaging data. a, Signal traces from simultaneous electrophysiological recording and voltage imaging. From top to bottom: Electrophysiological recording, traces extracted from raw data, SUPPORT-denoised data, and FIR low-pass filtered data. Filter orders are 10 for both FIR filters. Cutoff frequencies of Filter 1 and Filter 2 are 25Hz and 75Hz, respectively. Traces from voltage imaging data are used in matched filtering. **b**, Matched filtering outputs and extracted templates. Templates are obtained from the corresponding voltage traces. The salmon-colored areas behind the traces indicate the region of the threshold value used for F1 score calculation in **c**. **c**, F1 scores across threshold values used for spike detection.

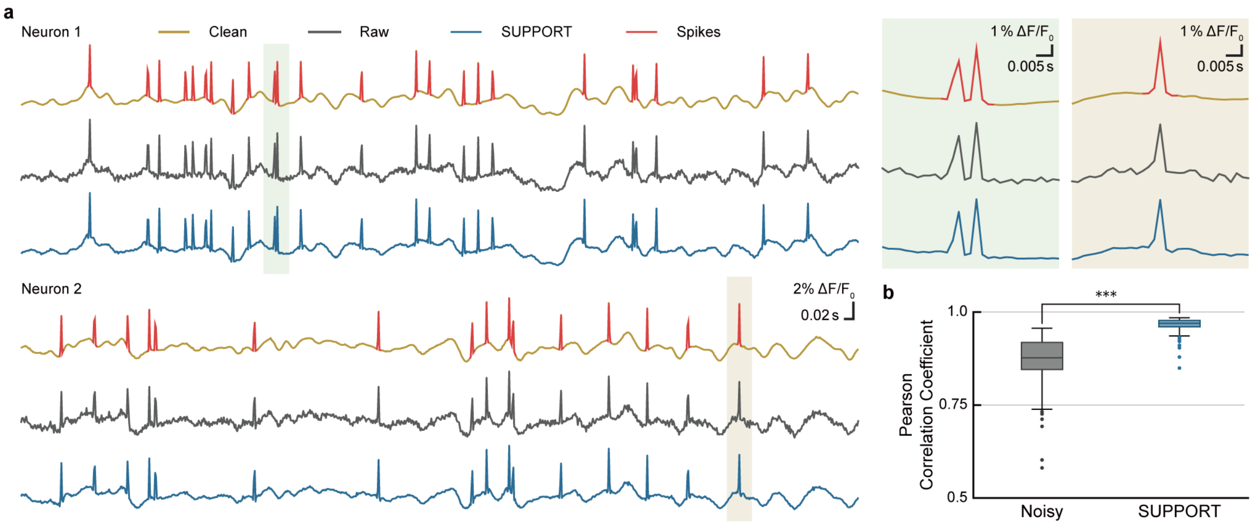

Supplementary Fig. 11: SUPPORT can be applied for subthreshold signal recovery from simulated voltage imaging data. a. Traces from raw and SUPPORT-denoised data extracted from two ROIs. Detected spike regions are indicated in red color. Spike regions are excluded in subthreshold analysis. **b**. Box-and-whisker plot showing Pearson correlation coefficient of subthreshold regions. A two-sided paired-sample t-test is used (***, p-value<0.001). N=116, which represents the number of pixels.

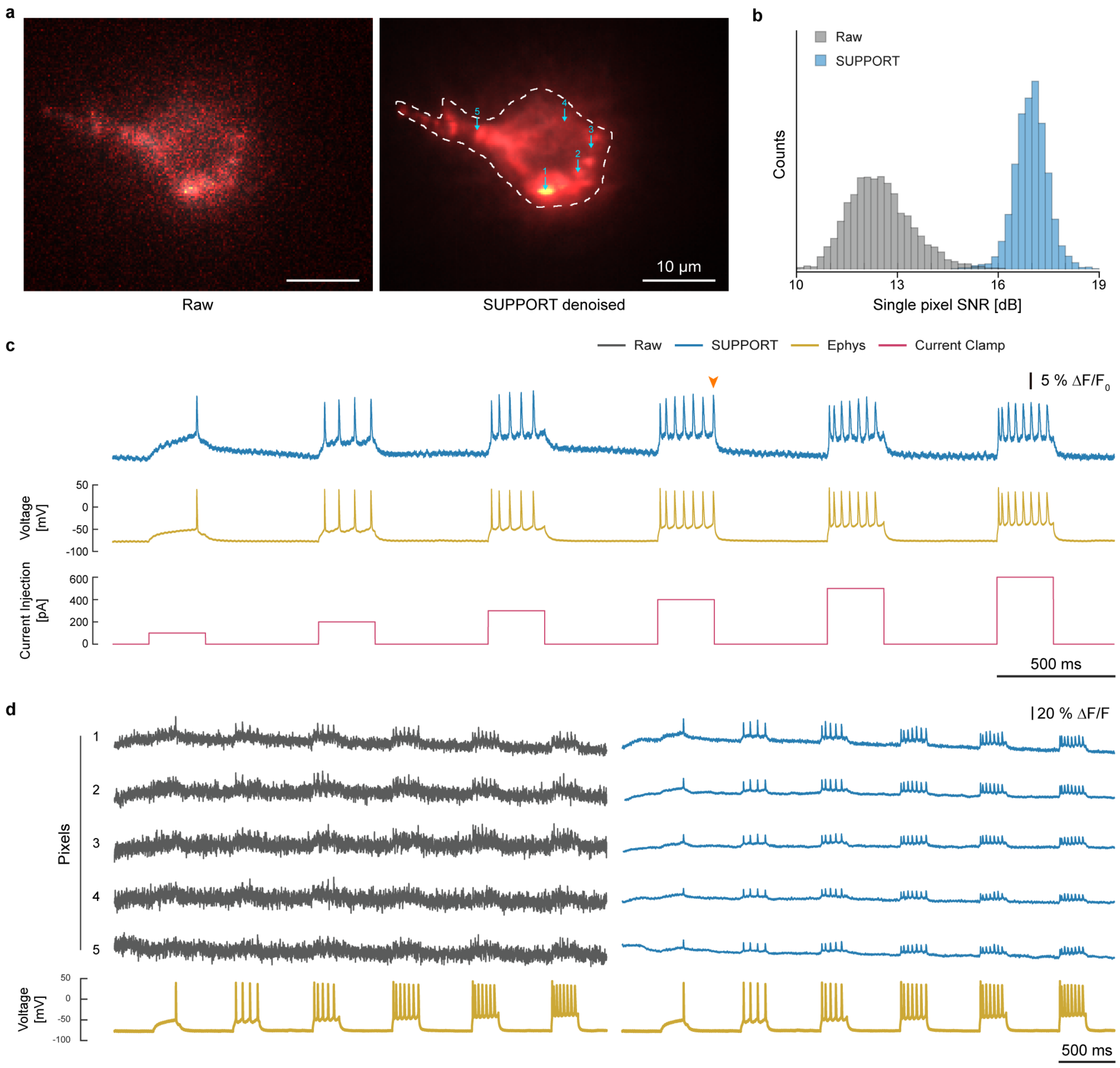

Supplementary Fig. 12: SUPPORT reveals single pixel traces from voltage imaging data with simultaneous electrophysiological recording. a, Representative frames of raw and SUPPORT-denoised videos are shown after baseline correction. The frames used are indicated on **c** with an orange arrow. QuasAr6a-expressing mouse cortex L2/3 was used as a dataset (Supplementary Table 1). The baseline component with gray colormap and the activity component with hot colormap are overlaid. The boundary of the region of interest (ROI) is drawn with a white dotted line. Five single pixels to be analyzed are marked with cyan arrows. **b**, Histogram of single pixel signal-to-noise ratio (SNR) from raw data and SUPPORT-denoised data. **c**, Traces extracted from SUPPORT-denoised data for the ROI in **a**, electrophysiological recording, injected currents. **d**, Traces extracted from raw and SUPPORT-denoised videos from single pixels in **a** and electrophysiological recording. Left: From raw video. Right: From SUPPORT-denoised video.

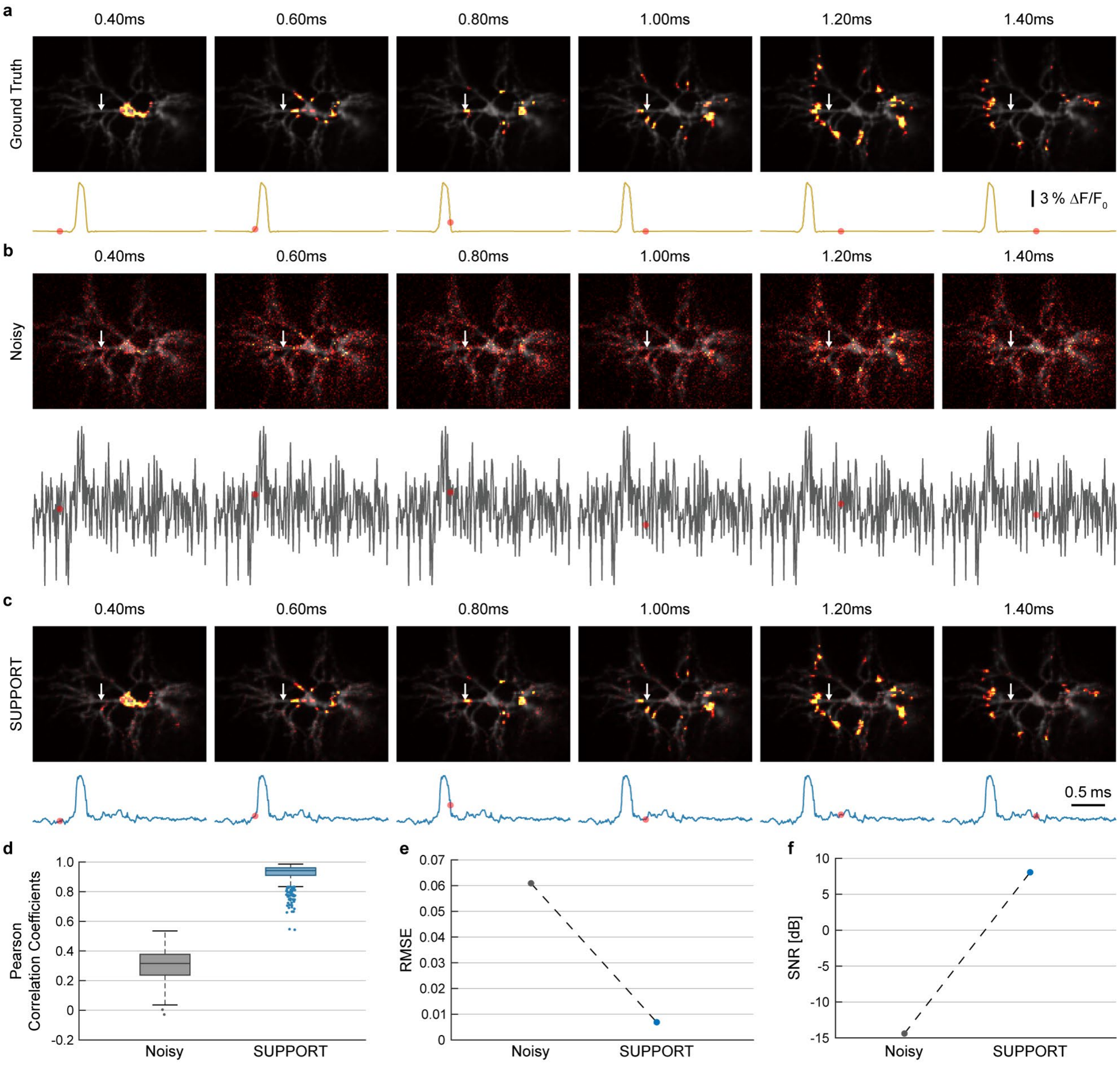

Supplementary Fig. 13: SUPPORT improves voltage imaging data during propagating action potential. a, Top: Synthetic voltage imaging data recorded at 100kHz, shown at six different time points. Baseline and activity components are decomposed from the data. The baseline component with a gray colormap and the activity component with a hot colormap are overlaid. Bottom: A trace extracted from the single pixel indicated by a white arrow was plotted. The red dot indicates the current frame. **b**, Gaussian noise added to the ground truth video is shown. **c**, Denoised videos using SUPPORT are shown. **d**, Box-and-whisker plot of single pixel Pearson correlation coefficients before and after denoising. N=12800 which represents the number of pixels. **e**, Line plot of single pixel RMSE before and after denoising. **f**, Line plot of single pixel SNR before and after denoising.

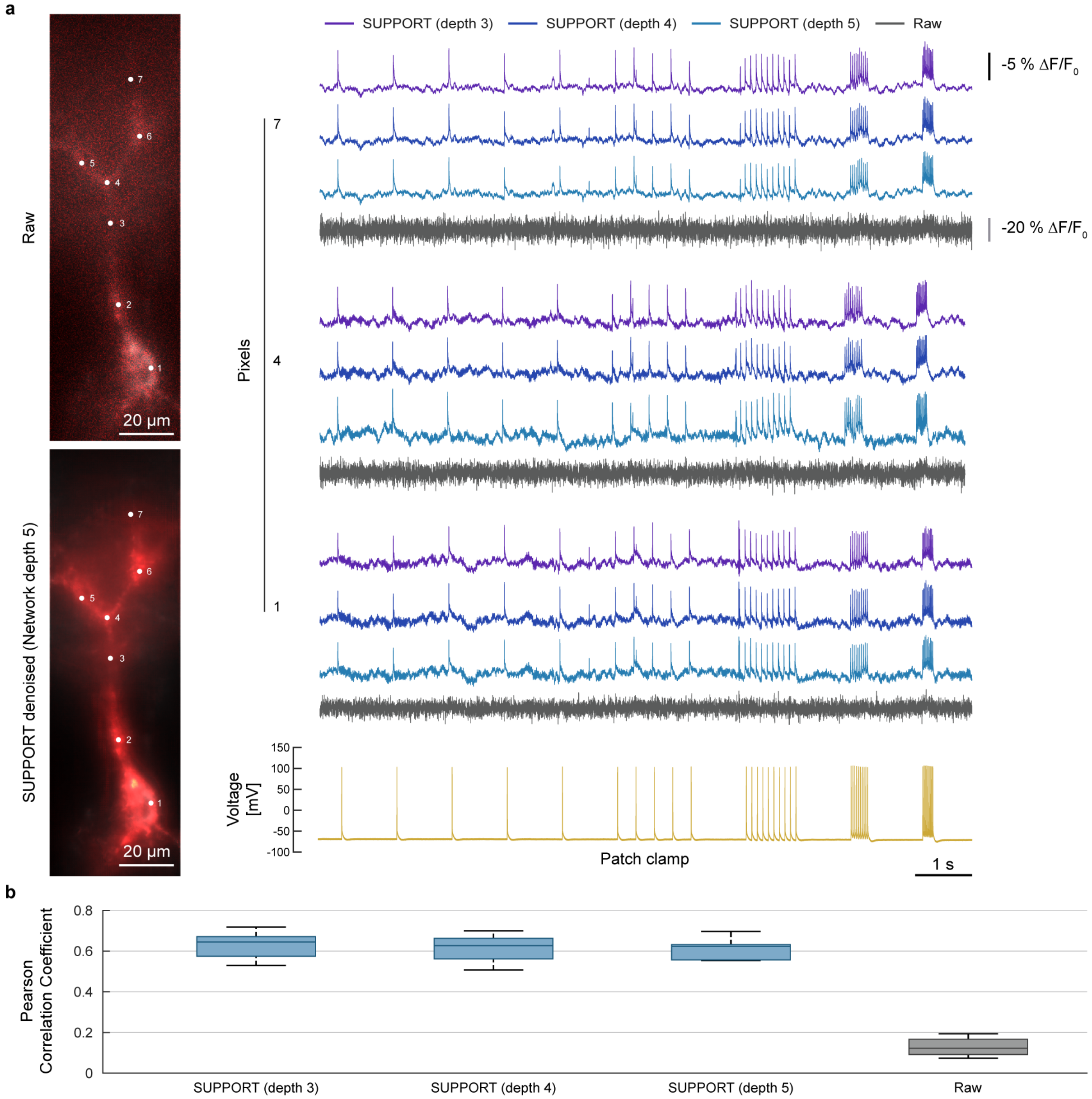

Supplementary Fig. 14: Denoising performance comparison of SUPPORT with various receptive field sizes in the XY-direction on single neuron voltage imaging data with dendritic branch. a, Left: A representative frame of raw and SUPPORT-denoised video after baseline correction. Voltron2-expressing mouse cortex L2/3 was used as a dataset (Supplementary Table 1). The baseline component with gray colormap and the activity component with hot colormap are overlaid. Single pixels to be analyzed along the dendritic branch are marked with white dots and numbers. Right: Traces extracted from the single pixels of denoised and raw video. Three different models of SUPPORT were used which exhibit different sizes of the receptive fields in the XY-direction. Bottom: Corresponding voltage traces from the electrophysiological recording. **b**, Box plot showing Pearson correlation coefficients between the traces extracted from the single pixels represented as a white dot in **a** and electrophysiological recording. N=7, which represents the number of pixels.

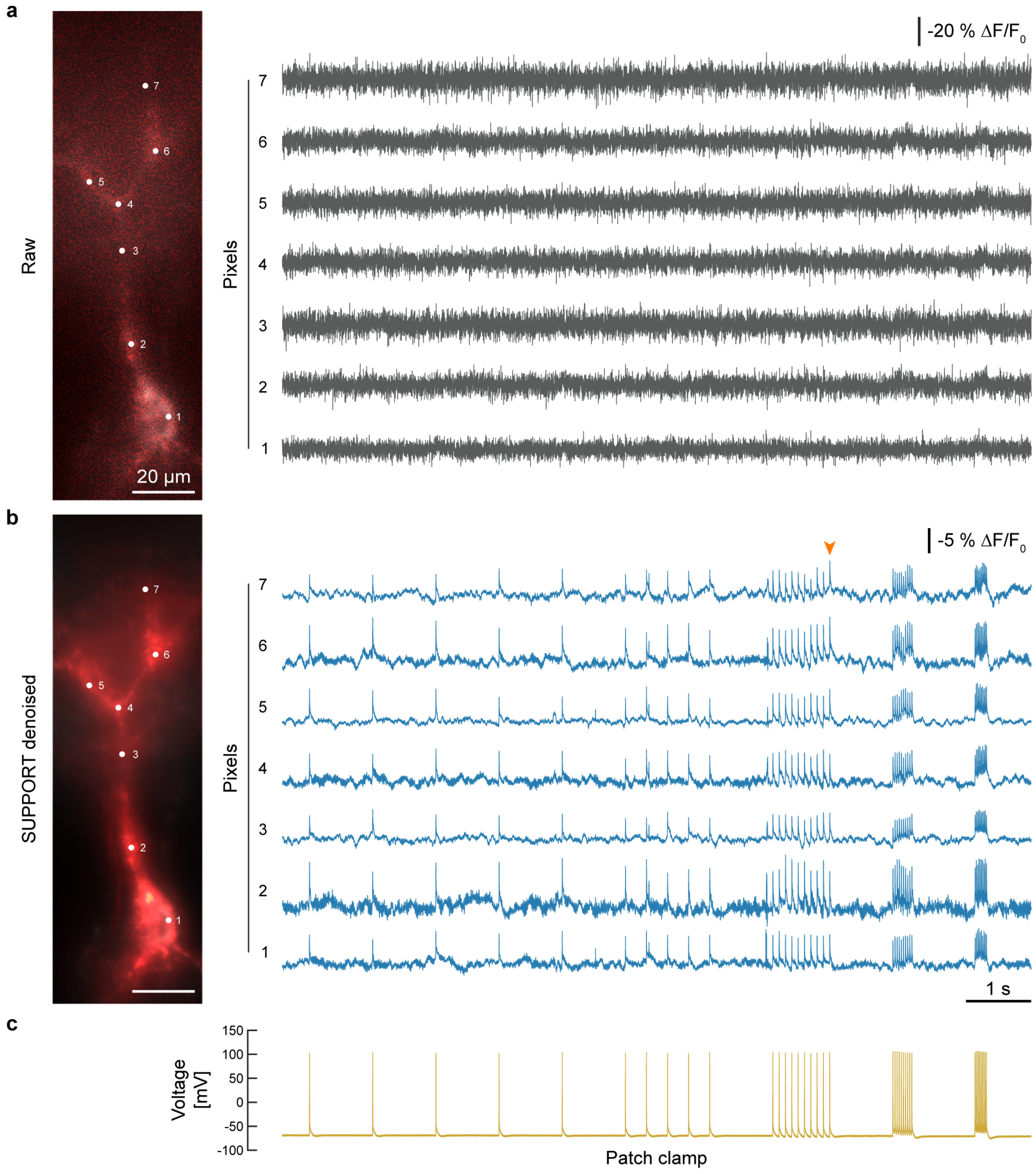

Supplementary Fig. 15: SUPPORT reveals the voltage signal along the dendritic branch. a, Left: A representative frame of raw video after baseline correction. The frame used is marked on **b** with an orange arrow. Voltron2-expressing mouse cortex L2/3 was used as a dataset (Supplementary Table 1). The baseline component with gray colormap and the activity component with hot colormap are overlaid. Single pixels to be analyzed along the dendritic branch are marked with white dots and numbers. Right: Traces extracted from the single pixels of raw video. **b**, Left: A representative frame of SUPPORT-denoised video after baseline correction. Right: Traces extracted from the single pixels of SUPPORT-denoised video. **c**,

Corresponding voltage traces from the electrophysiological recording.

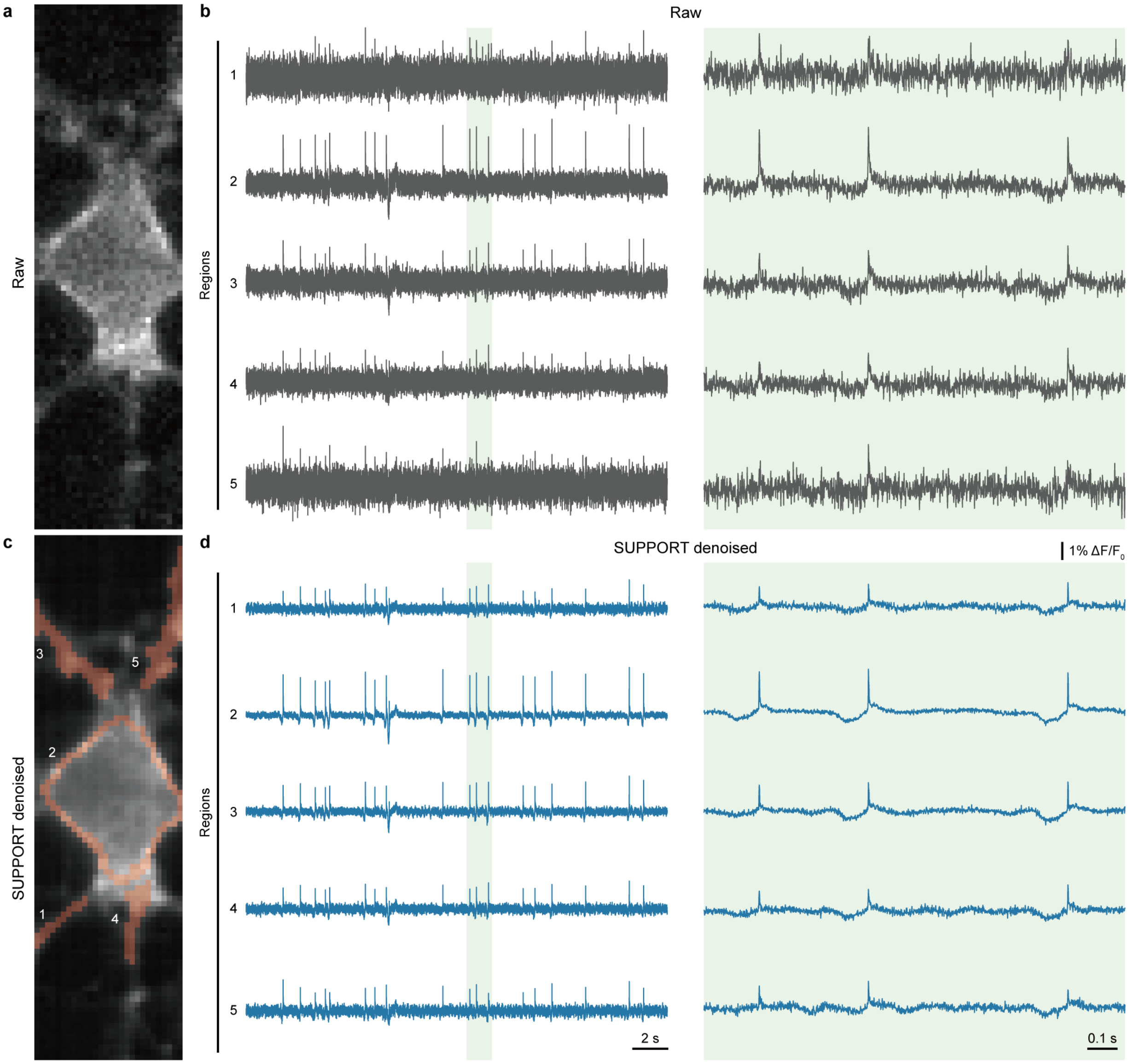

Supplementary Fig. 16: SUPPORT reveals the voltage signal in several regions. a, Representative frame of raw video. BeRST1-labeled mouse hippocampus cultured cells were used as a dataset (Supplementary Table 1). **b**, Traces extracted from the five regions of interest (ROIs) shown in **c** from the raw video. Temporally expanded traces from the green area on the left are shown on the right. **c**, Representative frame of SUPPORT-denoised video. ROIs to be analyzed are marked in orange. **d**, Left: Traces extracted from the five ROIs shown in **c** from the SUPPORT-denoised video. Right: Temporally expanded traces from the green area on the left.

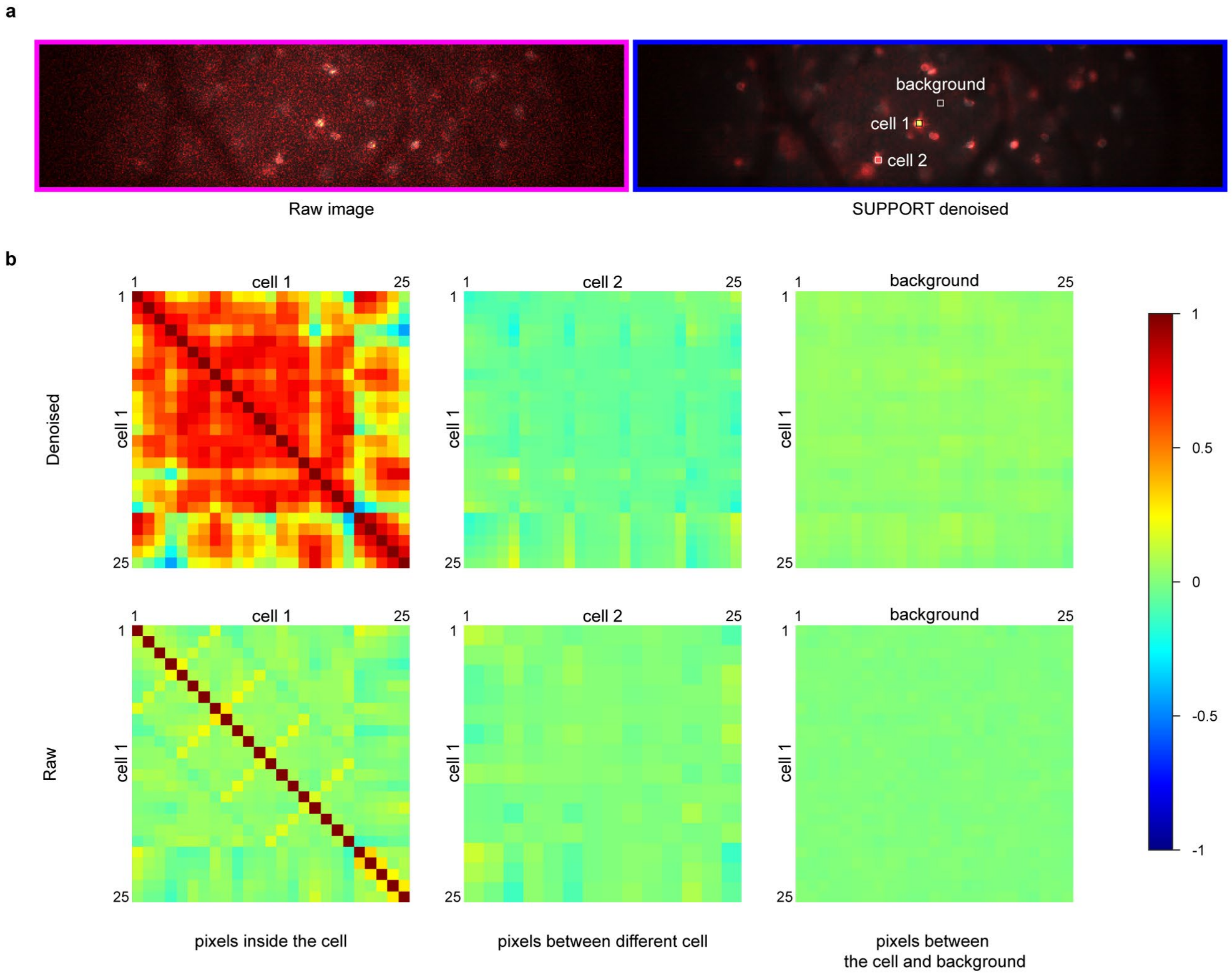

Supplementary Fig. 17: Pixelwise correlation coefficient map between different regions in voltage imaging data. a, Representative frames of raw video and SUPPORT-denoised video, for which the raw data was never shown to SUPPORT on training. Dataset of in vivo mouse cortex layer 1 expressing Voltron1 was used. Three regions which are cell1, cell2, and background were indicated with boxes. **b**, Pixel-level Pearson correlation coefficients. From left to right: Pearson correlation coefficients calculated between the same cell, between another cell, and between the background. Top: Pearson correlation coefficients calculated on SUPPORT-denoised video. Bottom: Pearson correlation coefficients calculated on the raw video.

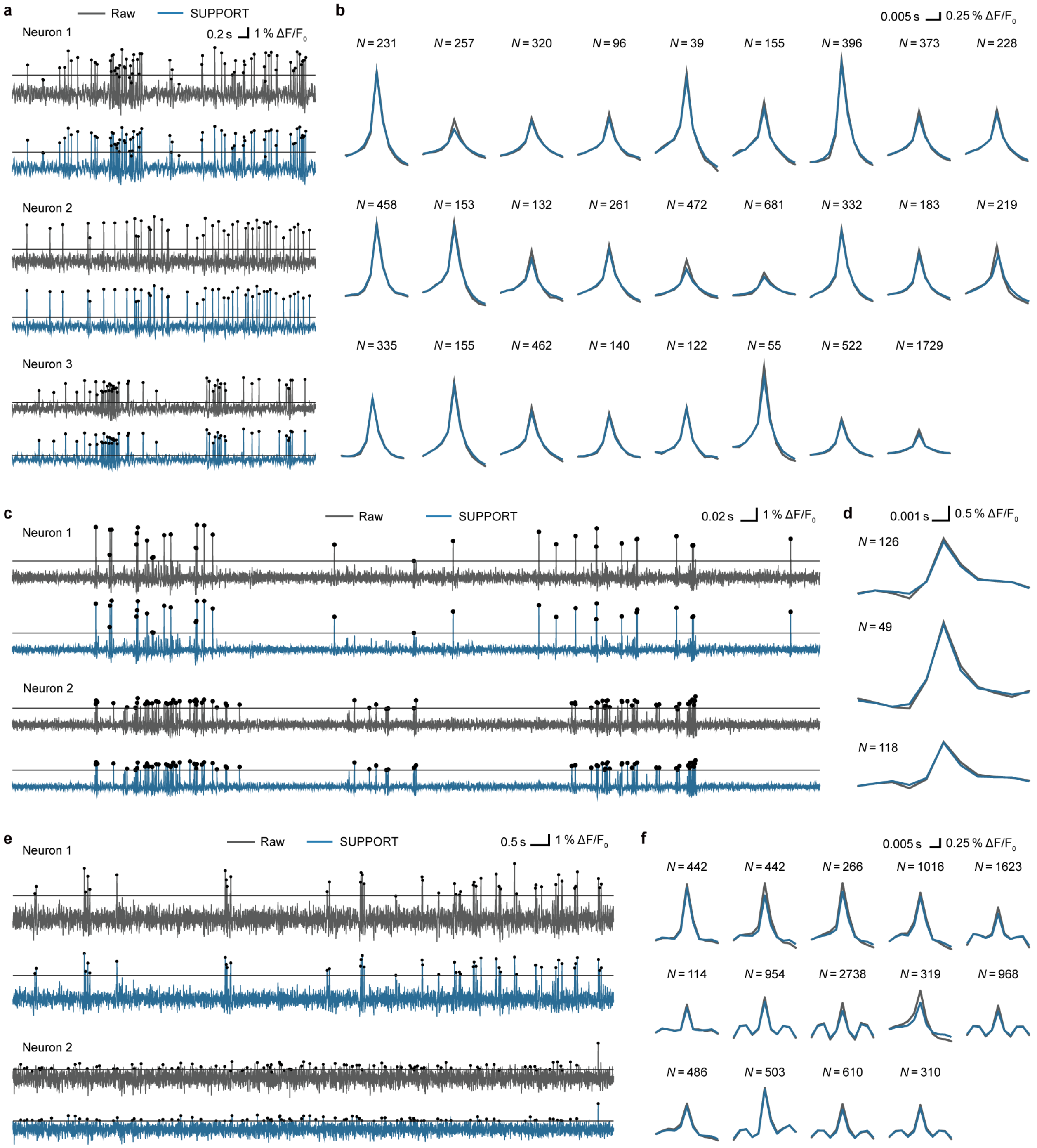

Supplementary Fig. 18: SUPPORT preserves temporal spike shapes in voltage imaging data. a, Traces extracted from representative 3 neurons from mouse dataset. Spikes are detected by thresholding the clean trace and finding local maximum locations. **b**, Average of spikes of all 26 neurons from raw and SUPPORT-denoised data. **c**, Traces extracted from representative 2 neurons from population voltage imaging data with paQuasAr3s. **d**, Average of spikes of all 3 neurons from raw and SUPPORT-denoised data. **e**, Traces extracted from representative 2 neurons from population voltage imaging data with Voltron1. **f**, Average of

spikes of 14 neurons from raw and SUPPORT-denoised data. Neurons with less than 100 spikes or dF/F0 lower than 2% were excluded.

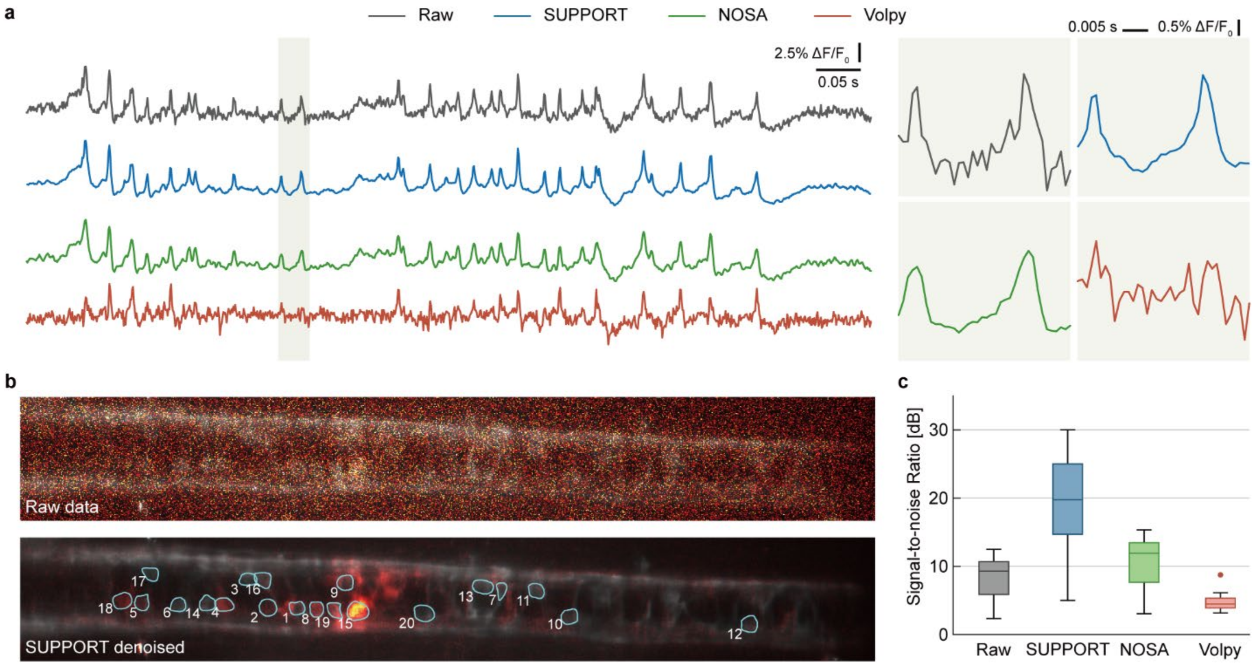

Supplementary Fig. 19: SUPPORT performance comparison with other voltage imaging data analysis algorithms on zebrafish dataset. a, Traces from raw, SUPPORT, NOSA, and Volpy denoised data. Enlarged view of the gray region in a is displayed on the right side. **b**, Images from the zebrafish dataset. Baseline and activity components are decomposed from raw data and SUPPORT-denoised data. The baseline component with a gray colormap and the activity component with a hot colormap are overlaid. Boundaries of 20 ROIs are drawn with cyan lines. **c**, Box-and-whisker plot showing signal-to-noise ratio of neuronal traces from raw, SUPPORT, NOSA, and Volpy denoised data. N=20, which represents the number of neurons.

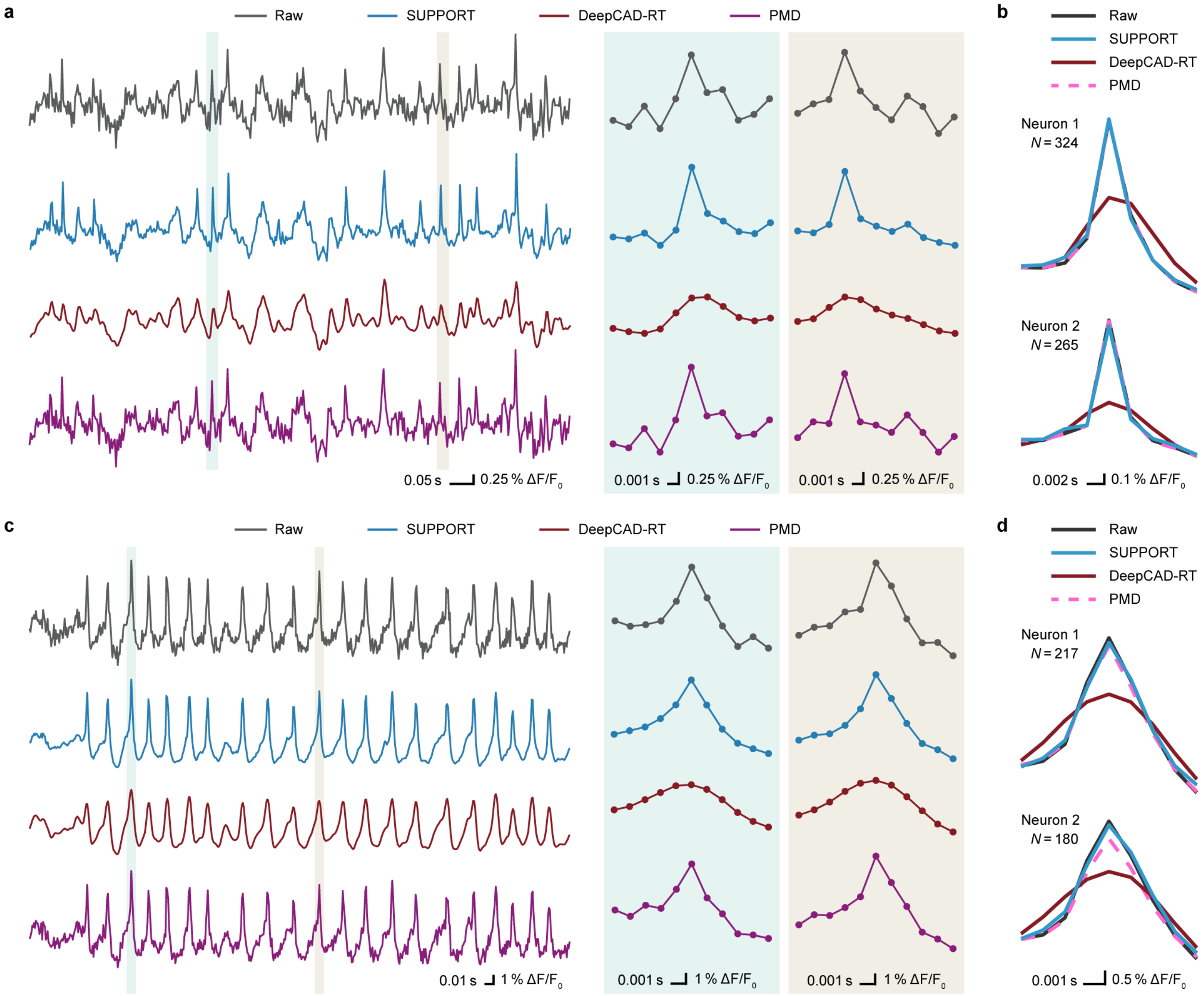

Supplementary Fig. 20: Spike shape analysis on experimental population voltage imaging data. a, Traces from raw and denoised data using SUPPORT, DeepCAD-RT, and PMD from mouse dataset. Traces for the smaller temporal region are plotted on the right. **b**, Average spike shapes of representative two neurons extracted from raw and denoised data. **c**, Traces from raw and denoised data from zebrafish dataset. **d**, Average spike shapes of representative two neurons extracted from raw and denoised data.

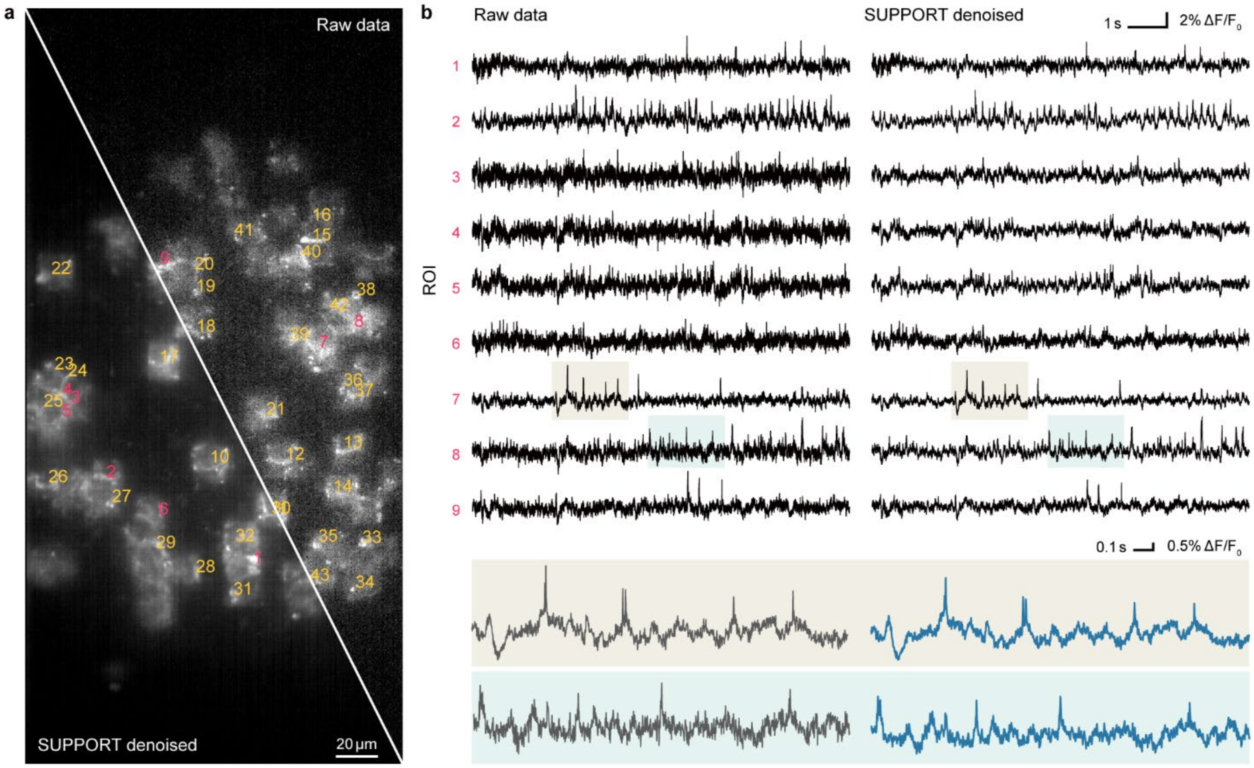

Supplementary Fig. 21: Applying SUPPORT to population voltage imaging data with SomArchon indicator. a, Cultured neurons expressing SomArchon indicator. **b**, Traces from 9 neurons before and after SUPPORT denoising. Traces for the smaller temporal regions are plotted below.

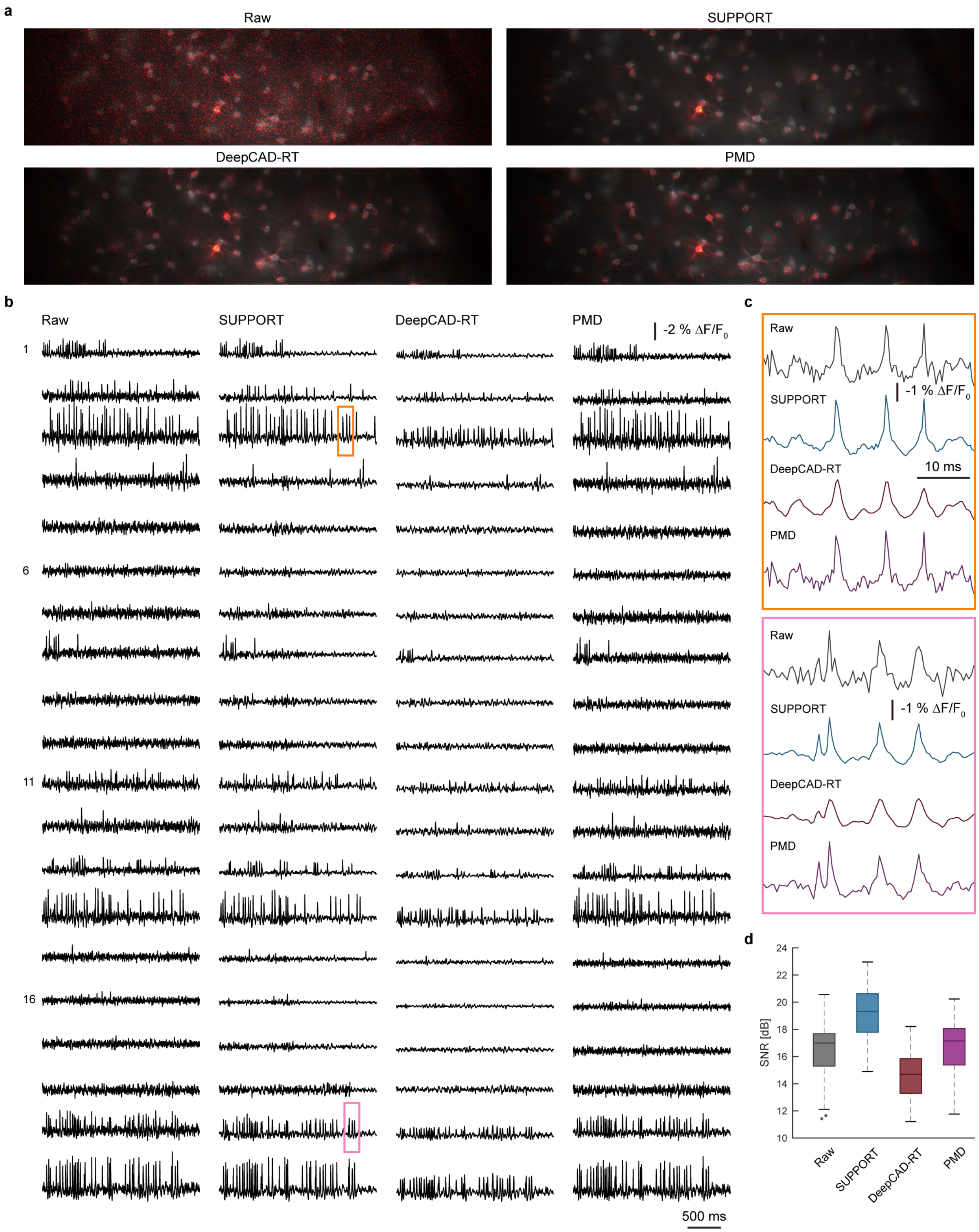

Supplementary Fig. 22: Denoising population mouse voltage imaging data. a, Images after baseline correction from mouse dataset expressing Voltron1 in cortex layer 1. **b**, Traces from 20 ROIs from raw,

SUPPORT, DeepCAD-RT, and PMD. **c**, Enlarged view of traces from colored box in **b** are plotted. **d**, Box-and-whisker plot showing the signal-to-noise ratio for the extracted traces. N=79, which represents the number of neurons.

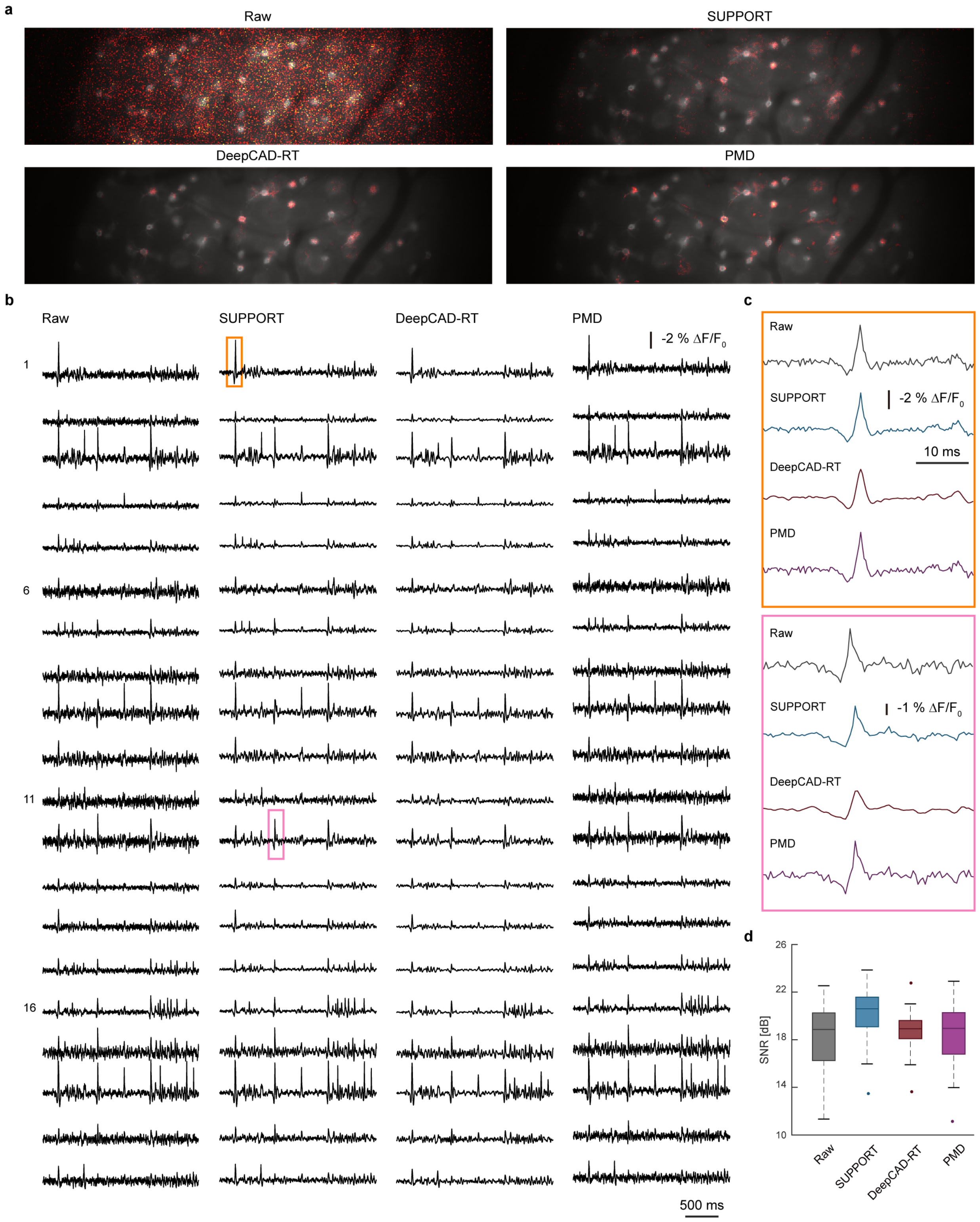

Supplementary Fig. 23: Denoising population mouse voltage imaging data. a, Images after baseline correction from mouse dataset expressing Voltron1 in cortex layer 1. **b**, Traces from 20 ROIs from raw,

SUPPORT, DeepCAD-RT, and PMD. **c**, Enlarged view of traces from colored box in **b** are plotted. **d**, Box-and-whisker plot showing the signal-to-noise ratio for the extracted traces. N=50, which represents the number of neurons.

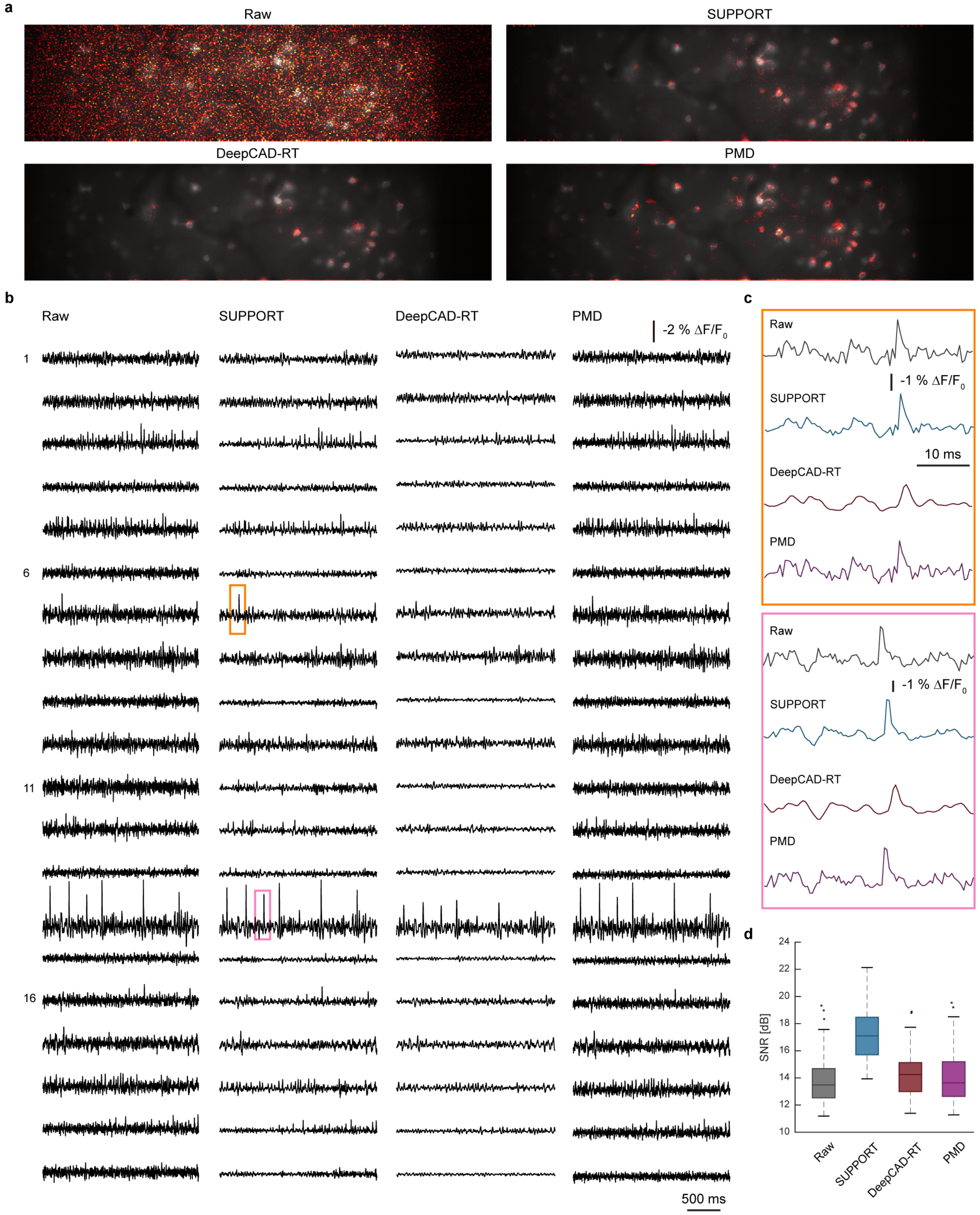

Supplementary Fig. 24: Denoising population mouse voltage imaging data. a, Images after baseline correction from mouse dataset expressing Voltron1 in cortex layer 1. **b**, Traces from 20 ROIs from raw,

SUPPORT, DeepCAD-RT, and PMD. **c**, Enlarged view of traces from colored box in **b** are plotted. **d**, Box-and-whisker plot showing the signal-to-noise ratio for the extracted traces. N=65, which represents the number of neurons.

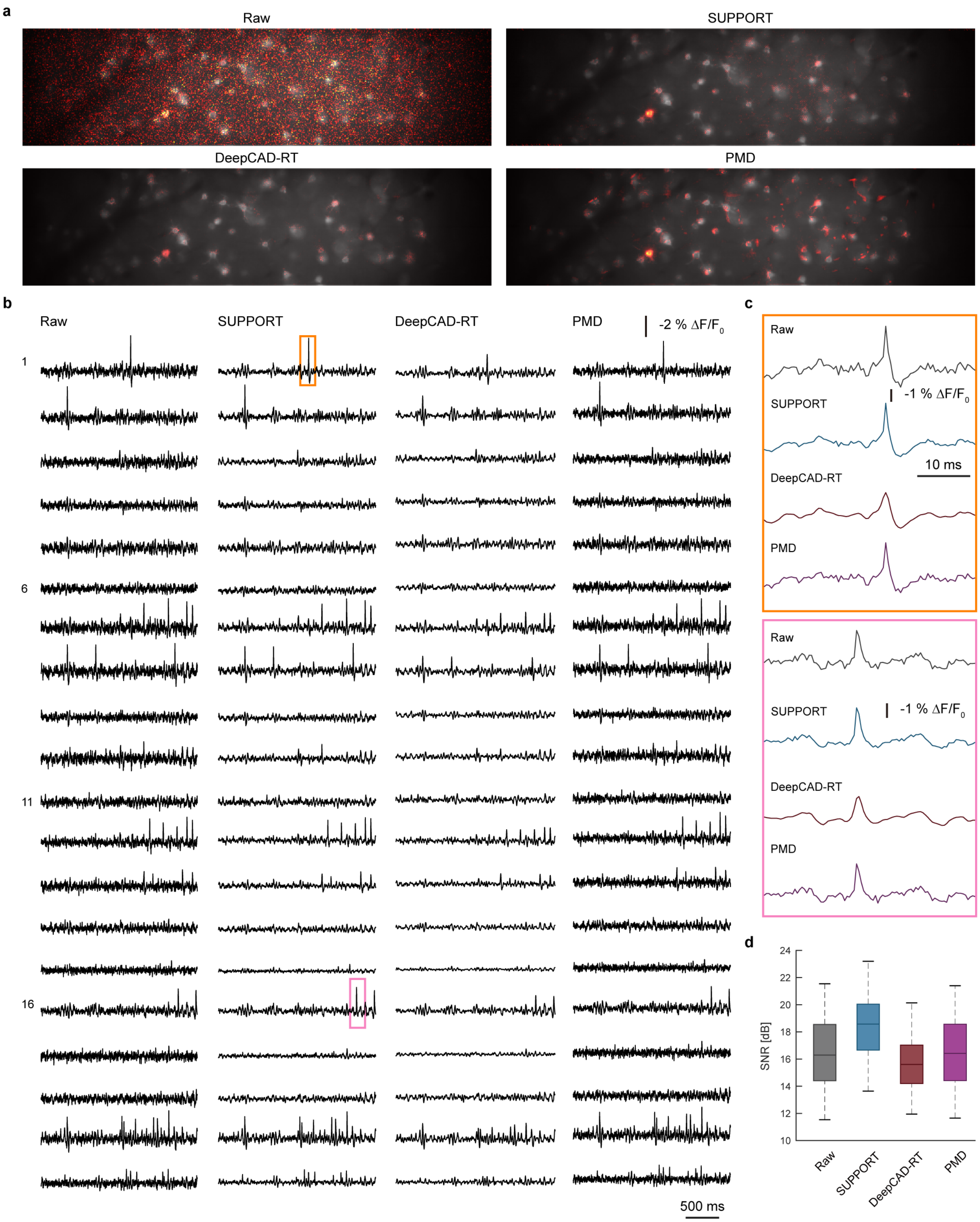

Supplementary Fig. 25: Denoising population mouse voltage imaging data. a, Images after baseline correction from mouse dataset expressing Voltron1 in cortex layer 1. **b**, Traces from 20 ROIs from raw,

SUPPORT, DeepCAD-RT, and PMD. **c**, Enlarged view of traces from colored box in **b** are plotted. **d**, Box-and-whisker plot showing the signal-to-noise ratio for the extracted traces. N=63, which represents the number of neurons.

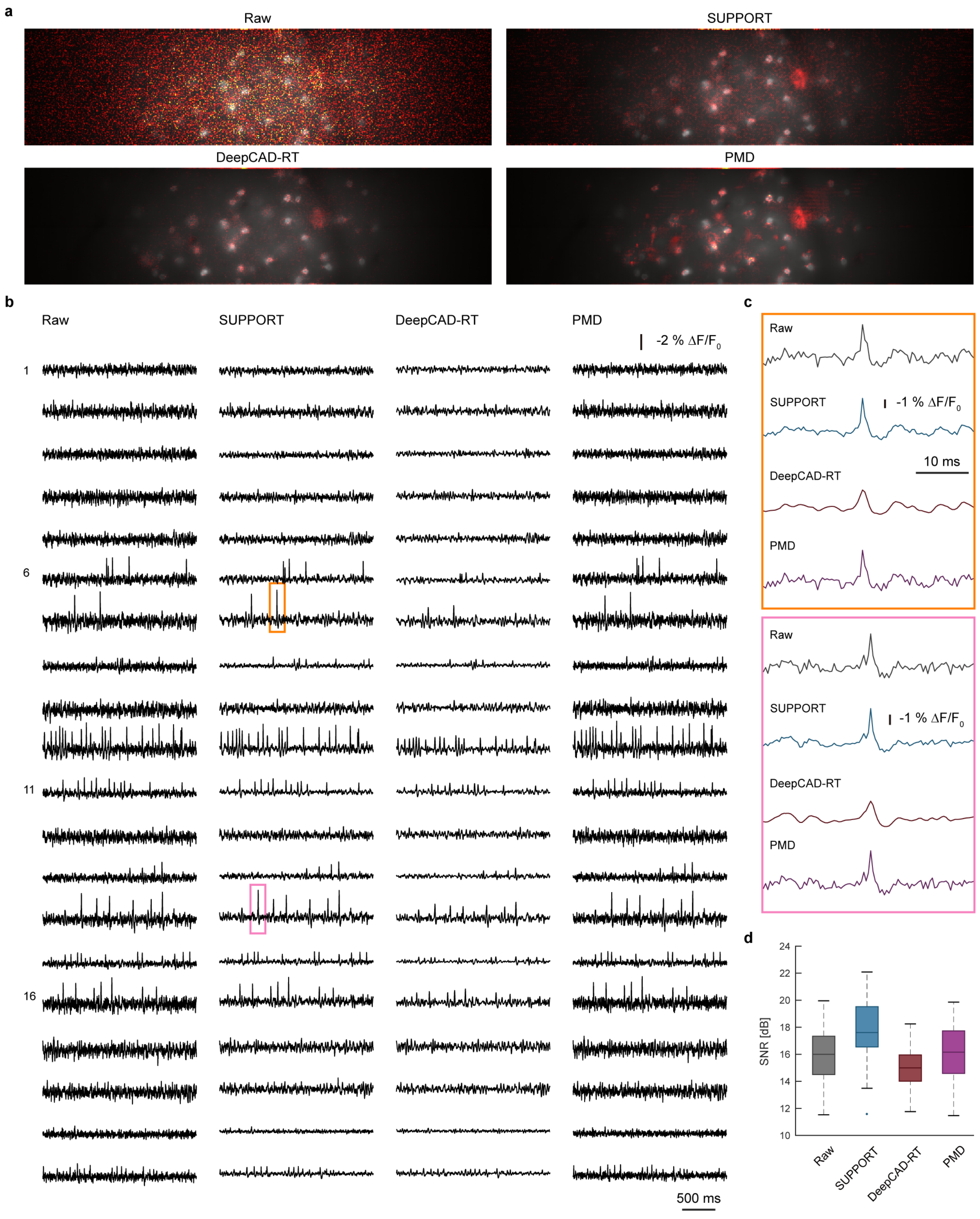

Supplementary Fig. 26: Denoising population mouse voltage imaging data. a, Images after baseline correction from mouse dataset expressing Voltron1 in cortex layer 1. **b**, Traces from 20 ROIs from raw,

SUPPORT, DeepCAD-RT, and PMD. **c**, Enlarged view of traces from colored box in **b** are plotted. **d**, Box-and-whisker plot showing the signal-to-noise ratio for the extracted traces. N=39, which represents the number of neurons.

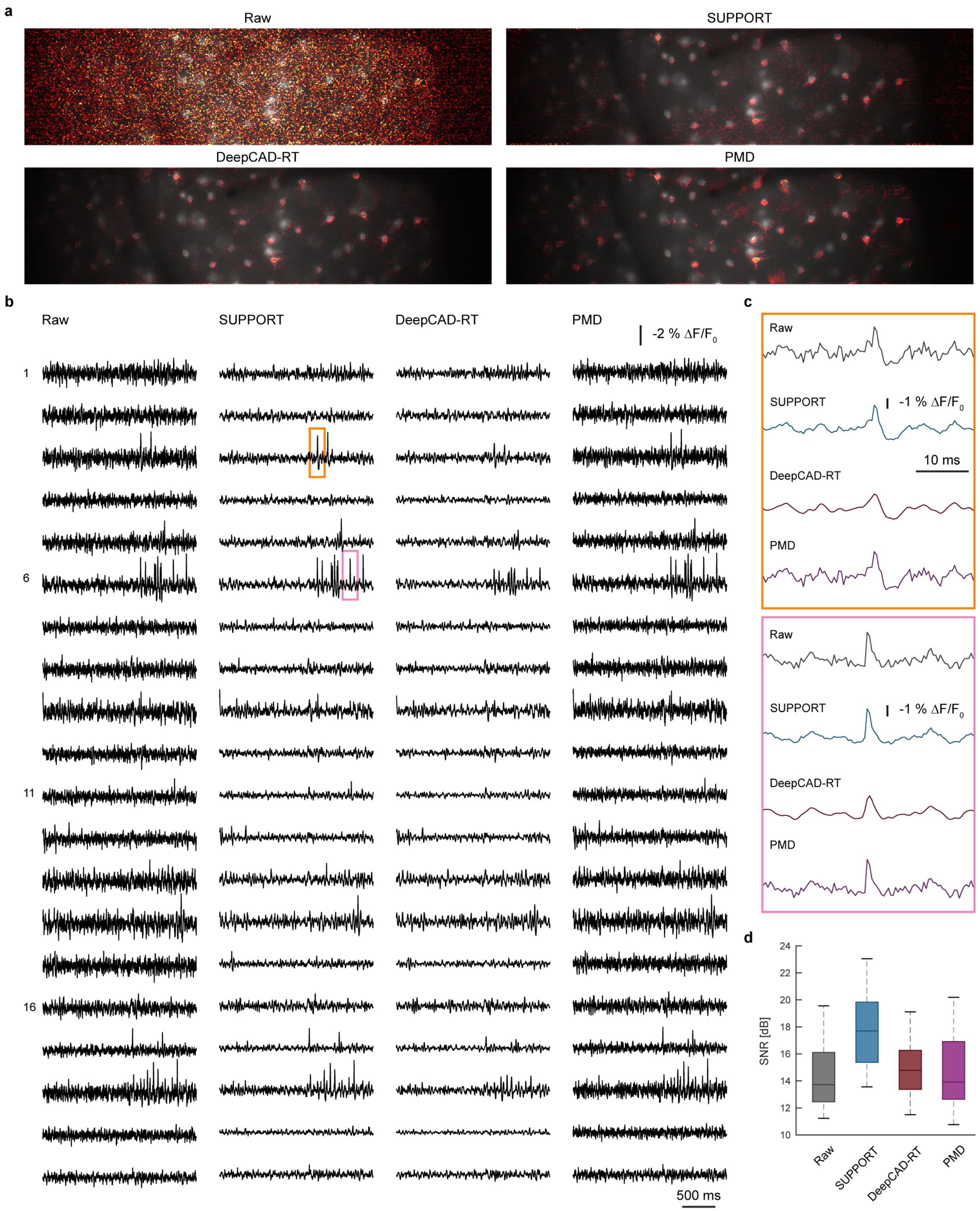

Supplementary Fig. 27: Denoising population mouse voltage imaging data. a, Images after baseline correction from mouse dataset expressing Voltron1 in cortex layer 1. **b**, Traces from 20 ROIs from raw,

SUPPORT, DeepCAD-RT, and PMD. **c**, Enlarged view of traces from colored box in **b** are plotted. **d**, Box-and-whisker plot showing the signal-to-noise ratio for the extracted traces. N=77, which represents the number of neurons.

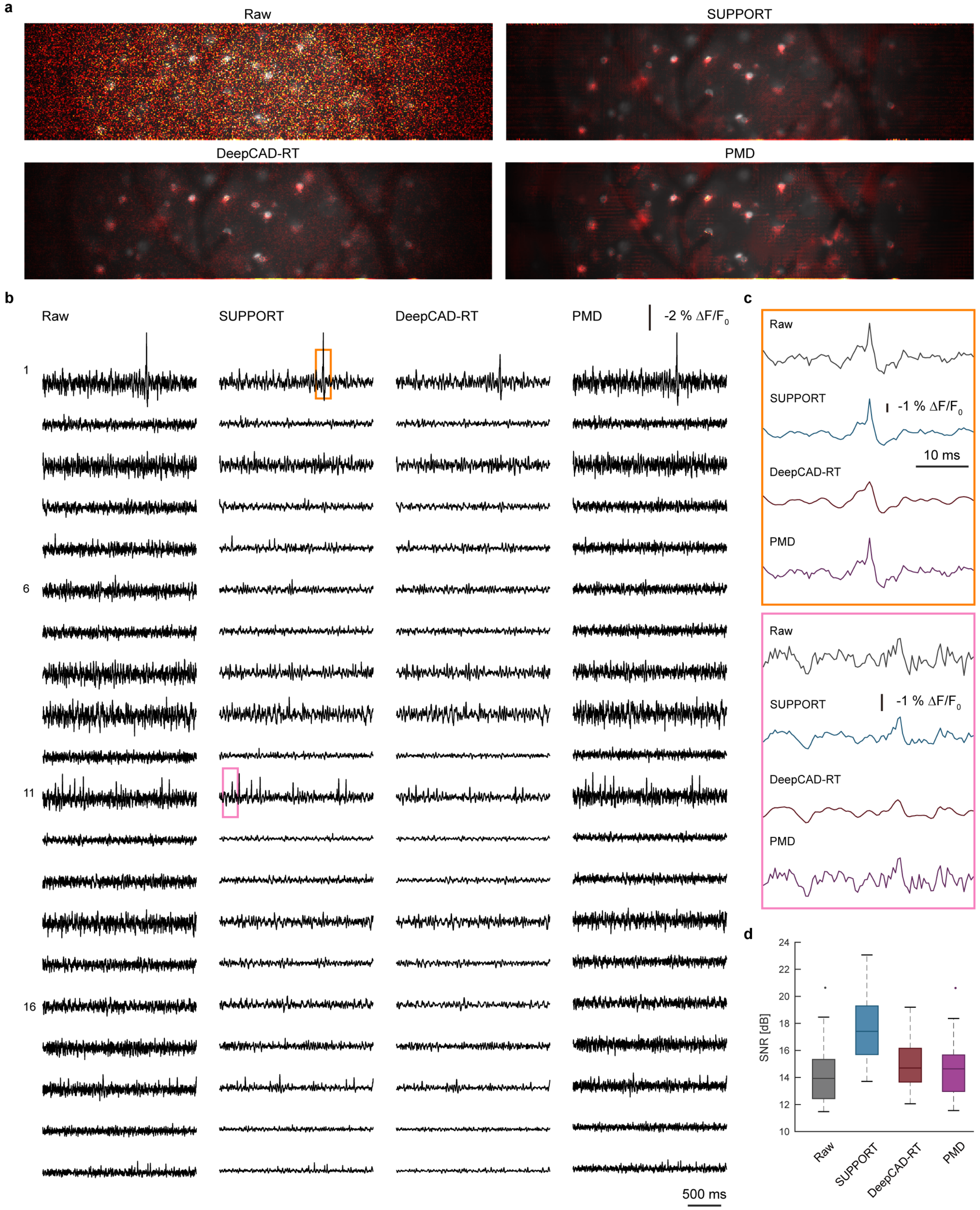

Supplementary Fig. 28: Denoising population mouse voltage imaging data. a, Images after baseline correction from mouse dataset expressing Voltron1 in cortex layer 1. **b**, Traces from 20 ROIs from raw,

SUPPORT, DeepCAD-RT, and PMD. **c**, Enlarged view of traces from colored box in **b** are plotted. **d**, Box-and-whisker plot showing the signal-to-noise ratio for the extracted traces. N=49, which represents the number of neurons.

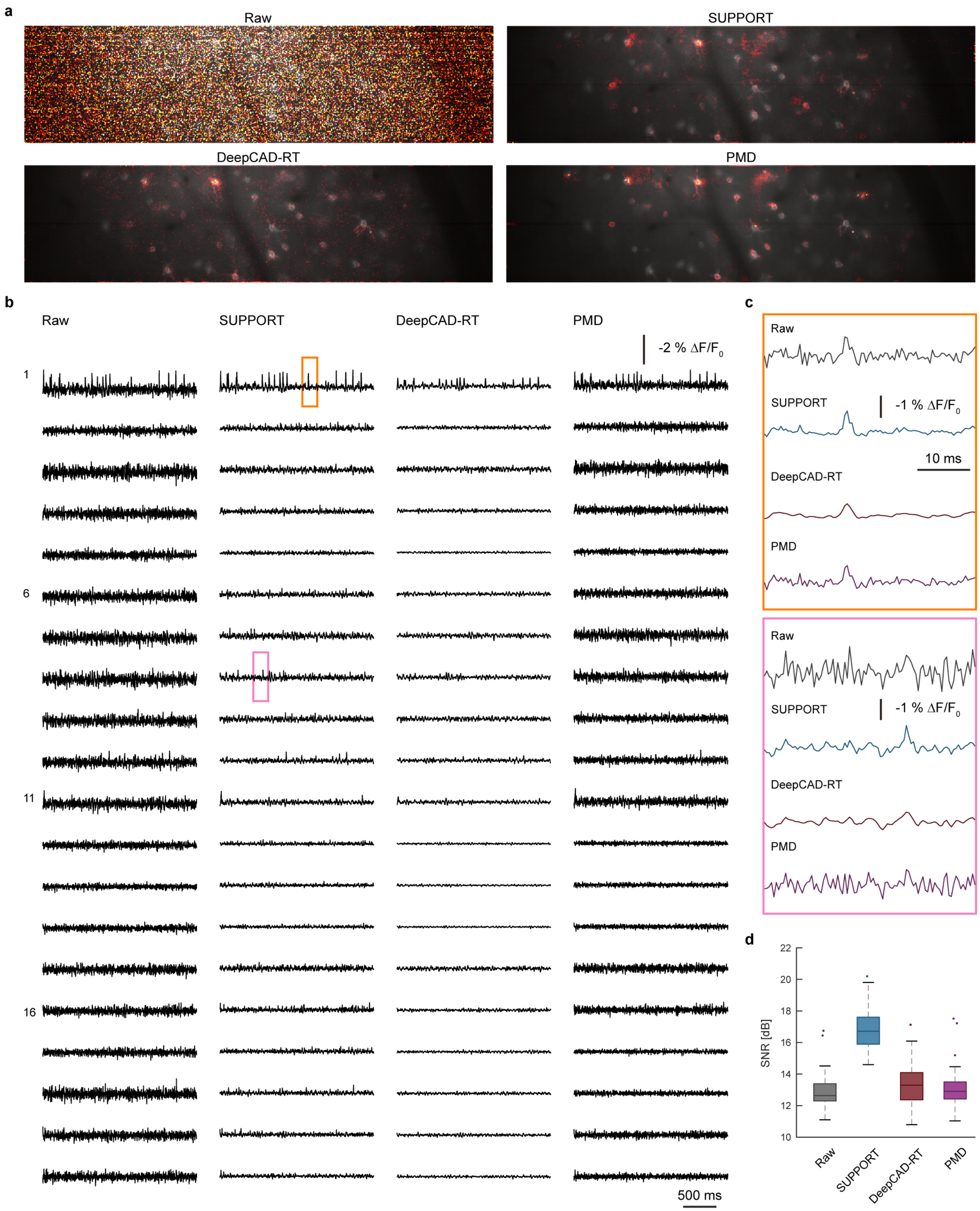

Supplementary Fig. 29: Denoising population mouse voltage imaging data. a, Images after baseline correction from mouse dataset expressing Voltron1 in cortex layer 1. **b**, Traces from 20 ROIs from raw,

SUPPORT, DeepCAD-RT, and PMD. **c**, Enlarged view of traces from colored box in **b** are plotted. **d**, Box-and-whisker plot showing the signal-to-noise ratio for the extracted traces. N=39, which represents the number of neurons.

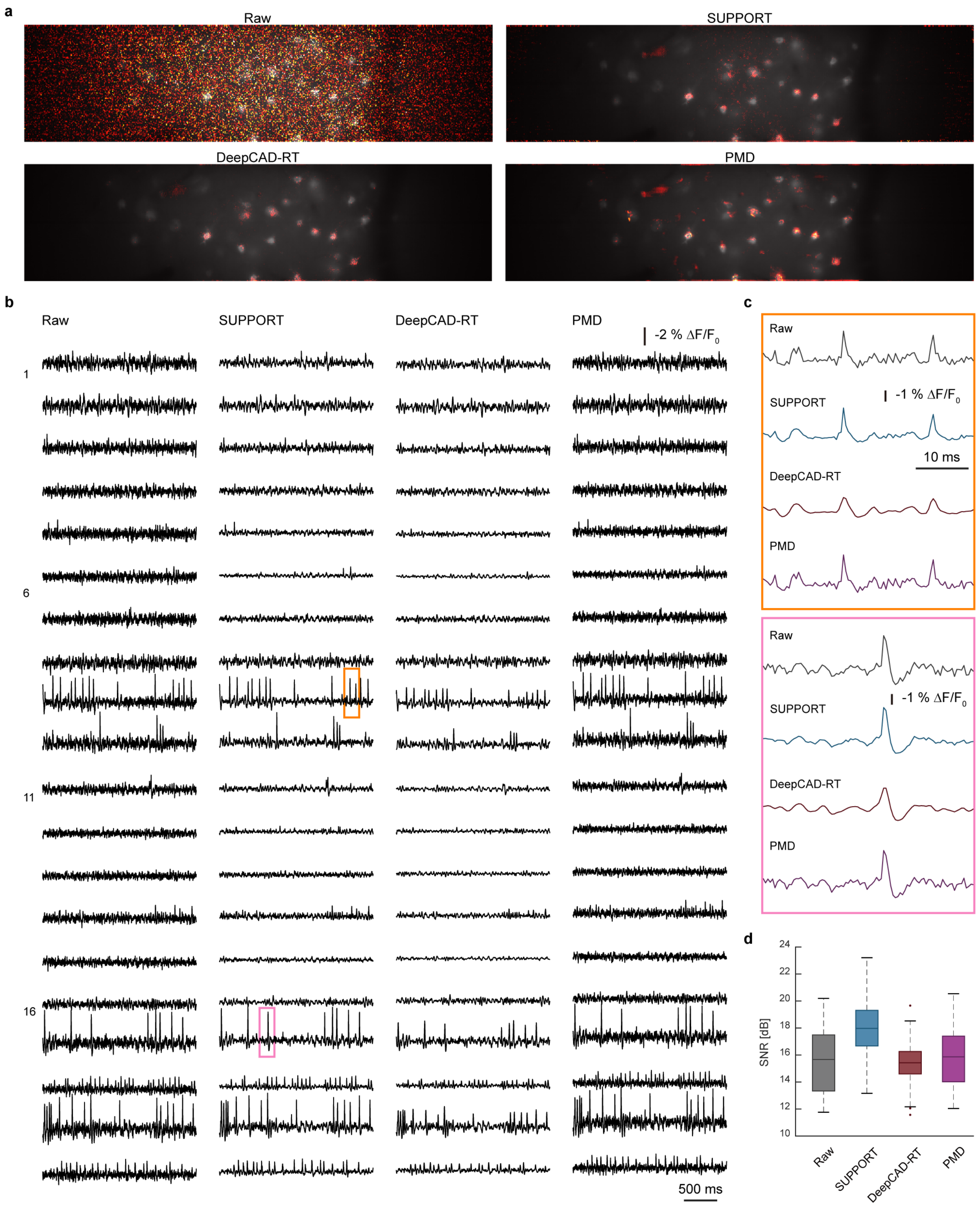

Supplementary Fig. 30: Denoising population mouse voltage imaging data. a, Images after baseline correction from mouse dataset expressing Voltron1 in cortex layer 1. **b**, Traces from 20 ROIs from raw,

SUPPORT, DeepCAD-RT, and PMD. **c**, Enlarged view of traces from colored box in **b** are plotted. **d**, Box-and-whisker plot showing the signal-to-noise ratio for the extracted traces. N=33, which represents the number of neurons.

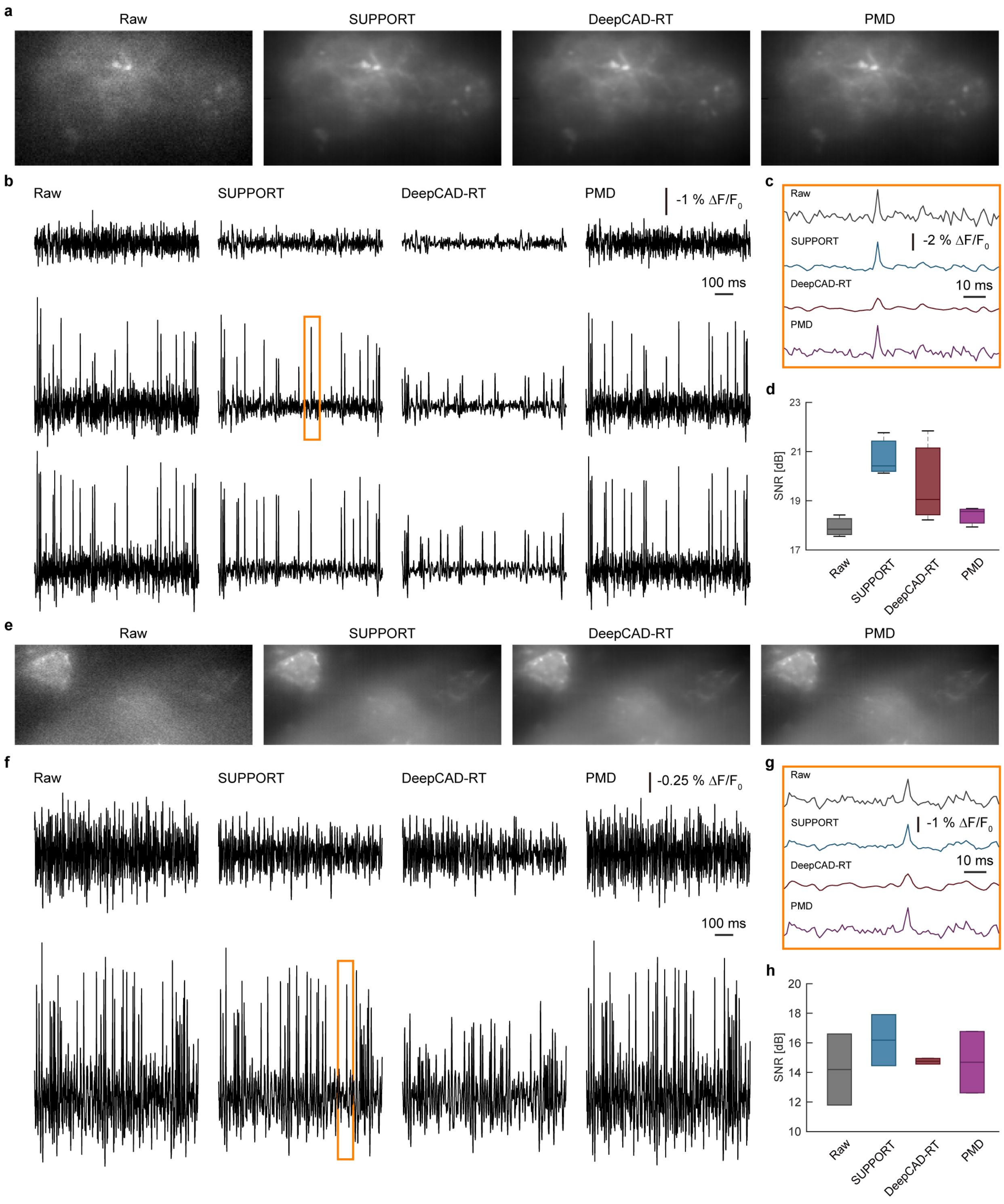

Supplementary Fig. 31: Denoising population mouse voltage imaging data. **a**, Images from mouse dataset expressing paQuasAr3s in hippocampus. **b**, Traces from 3 ROIs from raw, SUPPORT, DeepCAD-RT, and PMD. **c**, Enlarged view of traces from colored box in **b** are plotted. **d**, Box-and-whisker plot showing the signal-to-noise ratio for the extracted traces. N=3, which represents the number of neurons. **e**,

Images from mouse dataset expressing paQuasAr3s in hippocampus. **f**, Traces from 2 ROIs from raw, SUPPORT, DeepCAD-RT, and PMD. **g**, Enlarged view of traces from colored box in **f** are plotted. **h**, Box-and-whisker plot showing the signal-to-noise ratio for the extracted traces. N=2, which represents the number of neurons.

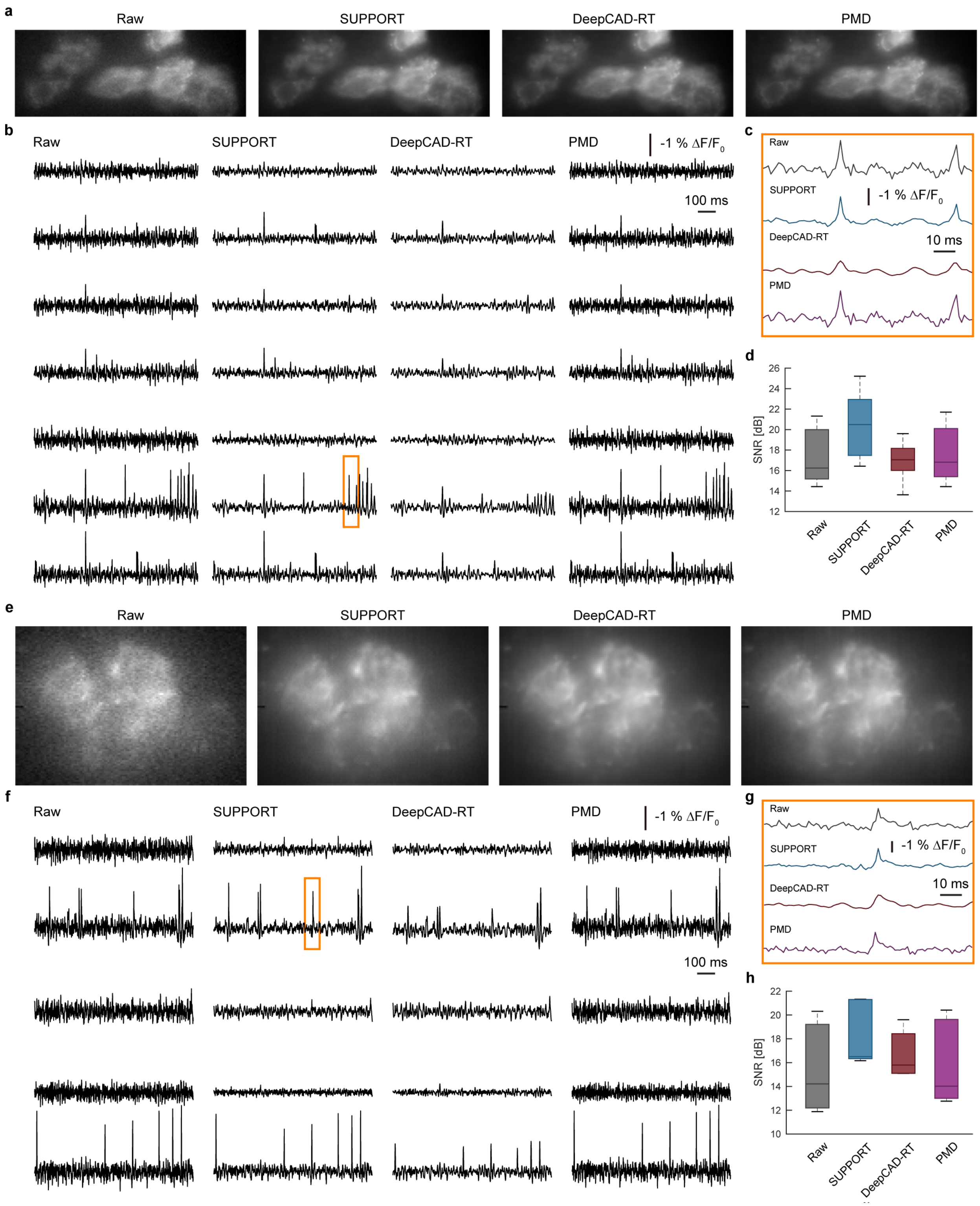

Supplementary Fig. 32: Denoising population mouse voltage imaging data. **a**, Images from mouse dataset expressing paQuasAr3s in hippocampus. **b**, Traces from 7 ROIs from raw, SUPPORT, DeepCAD-RT, and PMD. **c**, Enlarged view of traces from colored box in **b** are plotted. **d**, Box-and-whisker plot showing the signal-to-noise ratio for the extracted traces. N=7, which represents the number of neurons. **e**,

Images from mouse dataset expressing paQuasAr3s in hippocampus. **f**, Traces from 5 ROIs from raw, SUPPORT, DeepCAD-RT, and PMD. **g**, Enlarged view of traces from colored box in **f** are plotted. **h**, Box-and-whisker plot showing the signal-to-noise ratio for the extracted traces. N=5, which represents the number of neurons.

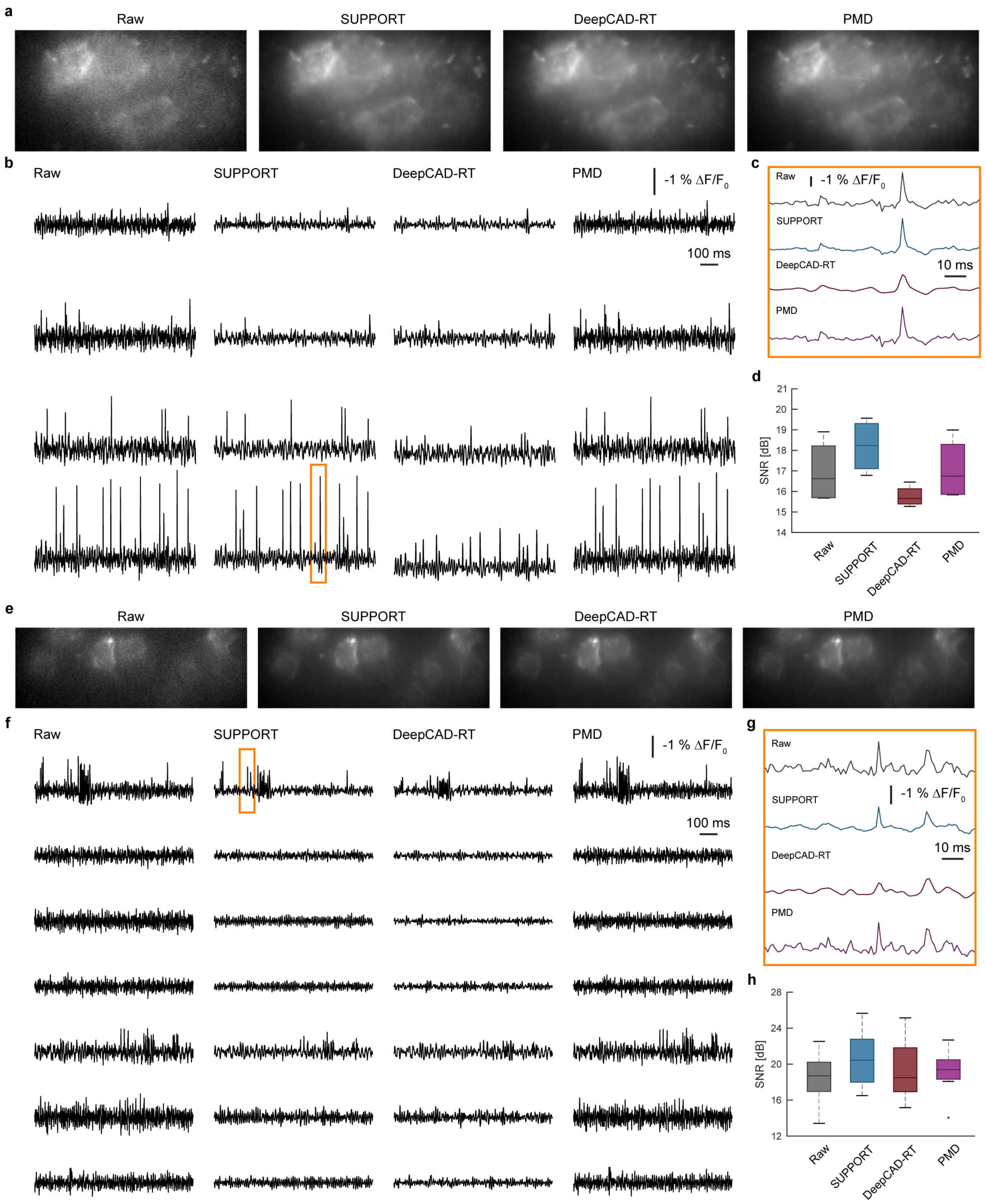

Supplementary Fig. 33: Denoising population mouse voltage imaging data. **a**, Images from mouse dataset expressing paQuasAr3s in hippocampus. **b**, Traces from 4 ROIs from raw, SUPPORT, DeepCAD-RT, and PMD. **c**, Enlarged view of traces from colored box in **b** are plotted. **d**, Box-and-whisker plot showing the signal-to-noise ratio for the extracted traces. N=4, which represents the number of neurons. **e**,

Images from mouse dataset expressing paQuasAr3s in hippocampus. **f**, Traces from 7 ROIs from raw, SUPPORT, DeepCAD-RT, and PMD. **g**, Enlarged view of traces from colored box in **f** are plotted. **h**, Box-and-whisker plot showing the signal-to-noise ratio for the extracted traces. N=7, which represents the number of neurons.

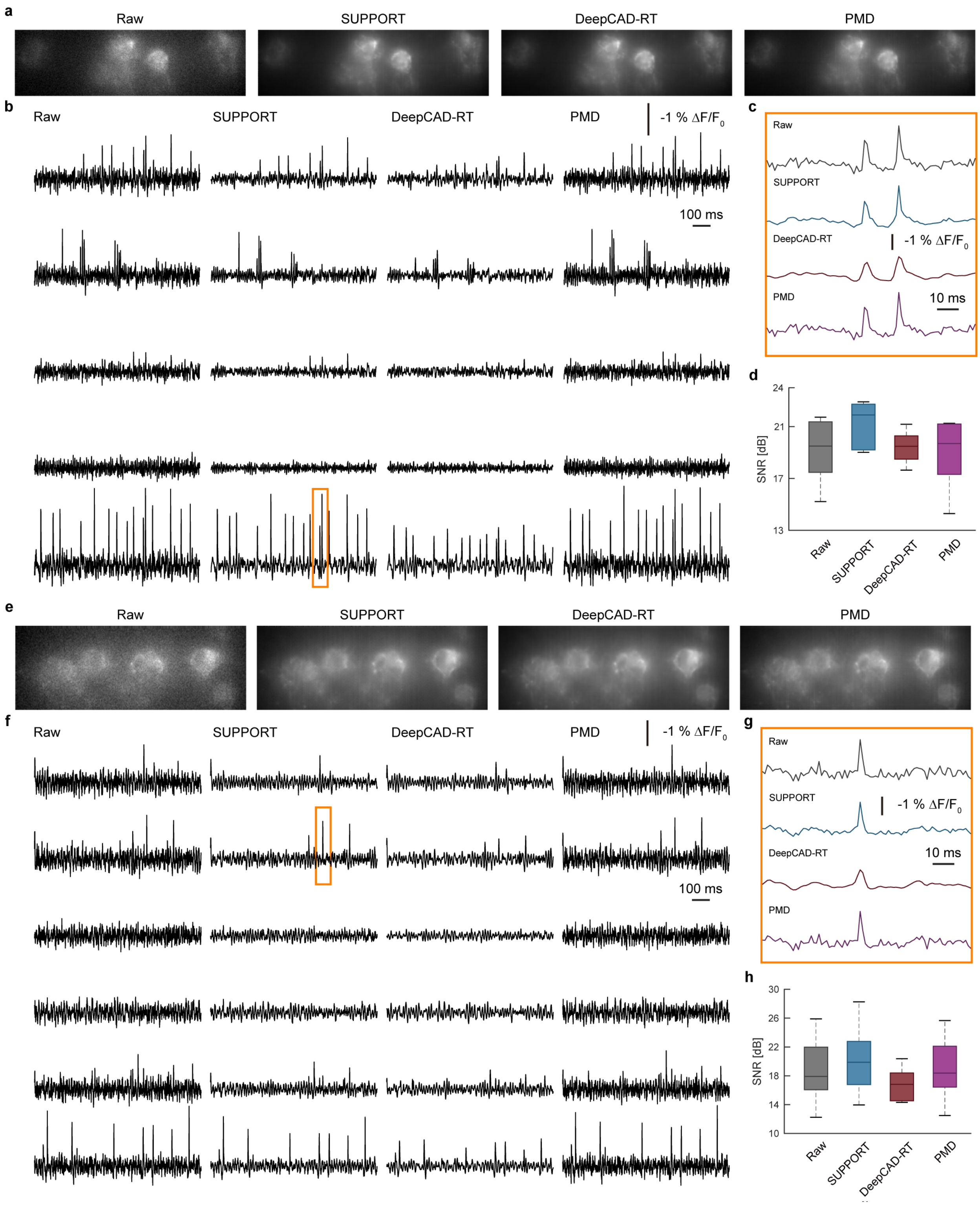

Supplementary Fig. 34: Denoising population mouse voltage imaging data. **a**, Images from mouse dataset expressing paQuasAr3s in hippocampus. **b**, Traces from 5 ROIs from raw, SUPPORT, DeepCAD-RT, and PMD. **c**, Enlarged view of traces from colored box in **b** are plotted. **d**, Box-and-whisker plot showing the signal-to-noise ratio for the extracted traces. N=5, which represents the number of neurons. **e**,

Images from mouse dataset expressing paQuasAr3s in hippocampus. **f**, Traces from 6 ROIs from raw, SUPPORT, DeepCAD-RT, and PMD. **g**, Enlarged view of traces from colored box in **f** are plotted. **h**, Box-and-whisker plot showing the signal-to-noise ratio for the extracted traces. N=5, which represents the number of neurons.

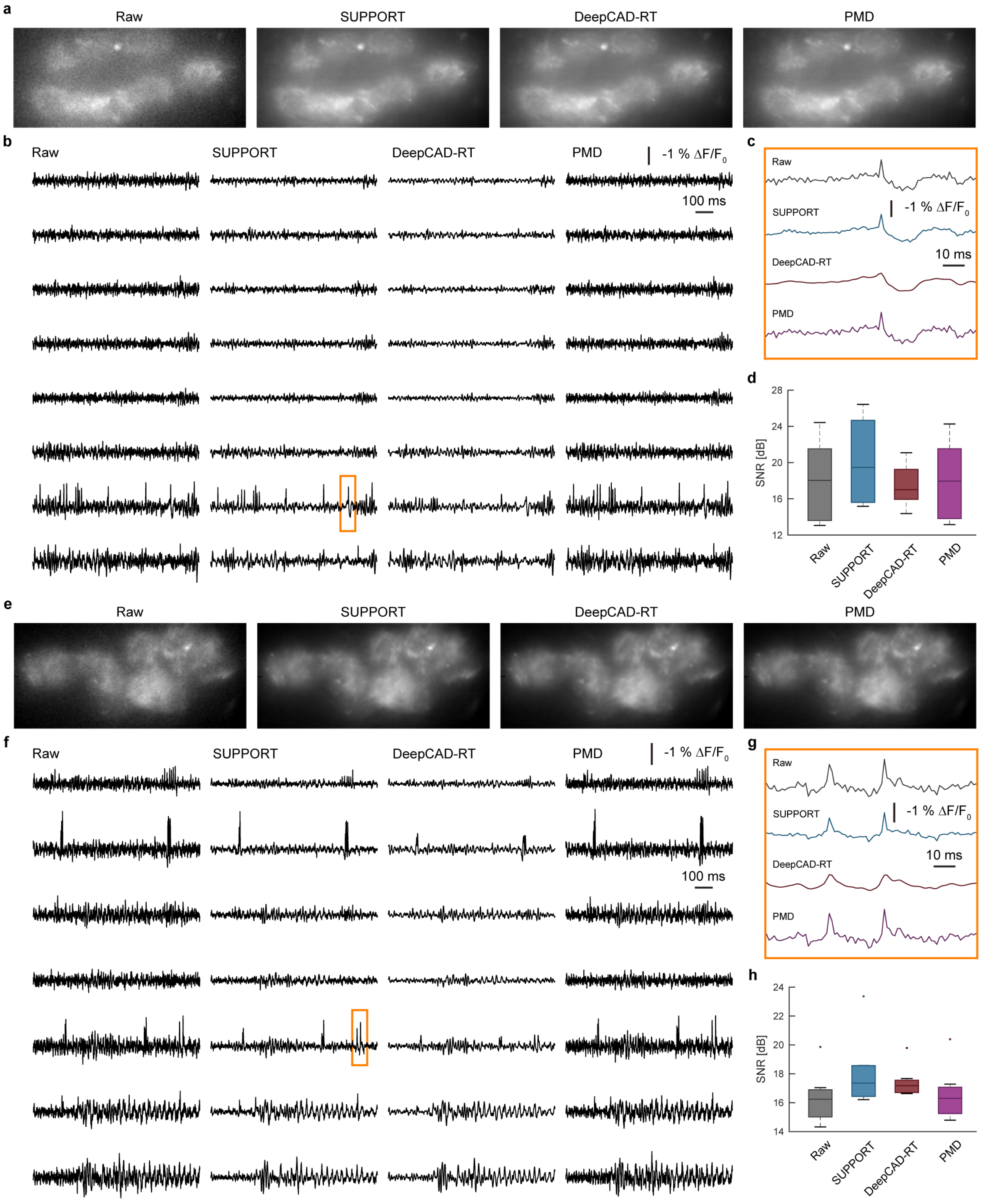

285

286 **Supplementary Fig. 35: Denoising population mouse voltage imaging data. a**, Images from mouse
287 dataset expressing paQuasAr3s in hippocampus. **b**, Traces from 8 ROIs from raw, SUPPORT, DeepCAD-
288 RT, and PMD. **c**, Enlarged view of traces from colored box in **b** are plotted. **d**, Box-and-whisker plot
289 showing the signal-to-noise ratio for the extracted traces. N=8, which represents the number of neurons. **e**,

Images from mouse dataset expressing paQuasAr3s in hippocampus. **f**, Traces from 7 ROIs from raw, SUPPORT, DeepCAD-RT, and PMD. **g**, Enlarged view of traces from colored box in **f** are plotted. **h**, Box-and-whisker plot showing the signal-to-noise ratio for the extracted traces. N=7, which represents the number of neurons.

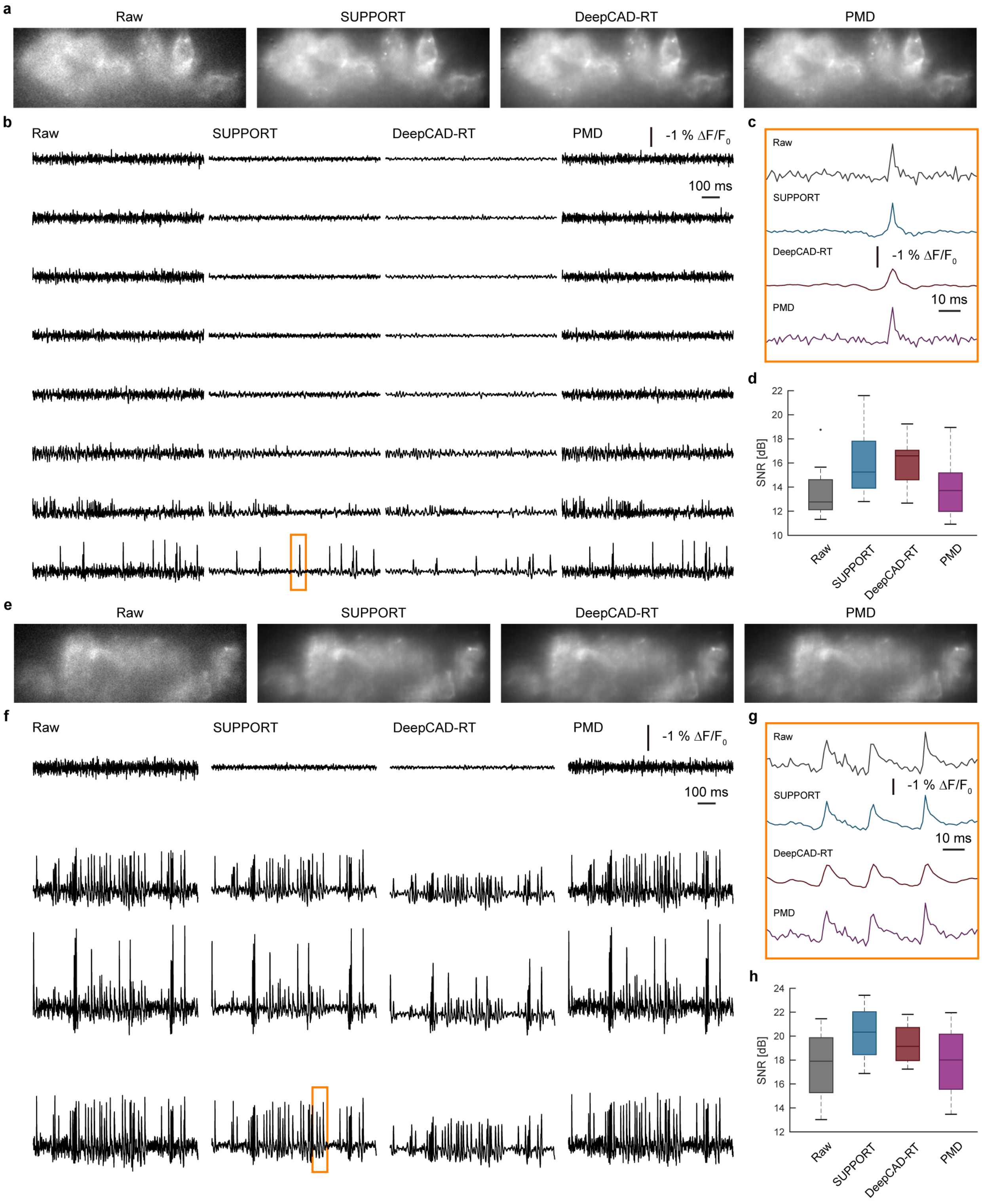

Supplementary Fig. 36: Denoising population mouse voltage imaging data. a, Images from mouse dataset expressing paQuasAr3s in hippocampus. **b**, Traces from 8 ROIs from raw, SUPPORT, DeepCAD-RT, and PMD. **c**, Enlarged view of traces from colored box in **b** are plotted. **d**, Box-and-whisker plot showing the signal-to-noise ratio for the extracted traces. N=8, which represents the number of neurons. **e**,

Images from mouse dataset expressing paQuasAr3s in hippocampus. **f**, Traces from 4 ROIs from raw, SUPPORT, DeepCAD-RT, and PMD. **g**, Enlarged view of traces from colored box in **f** are plotted. **h**, Box-and-whisker plot showing the signal-to-noise ratio for the extracted traces. N=4, which represents the number of neurons.

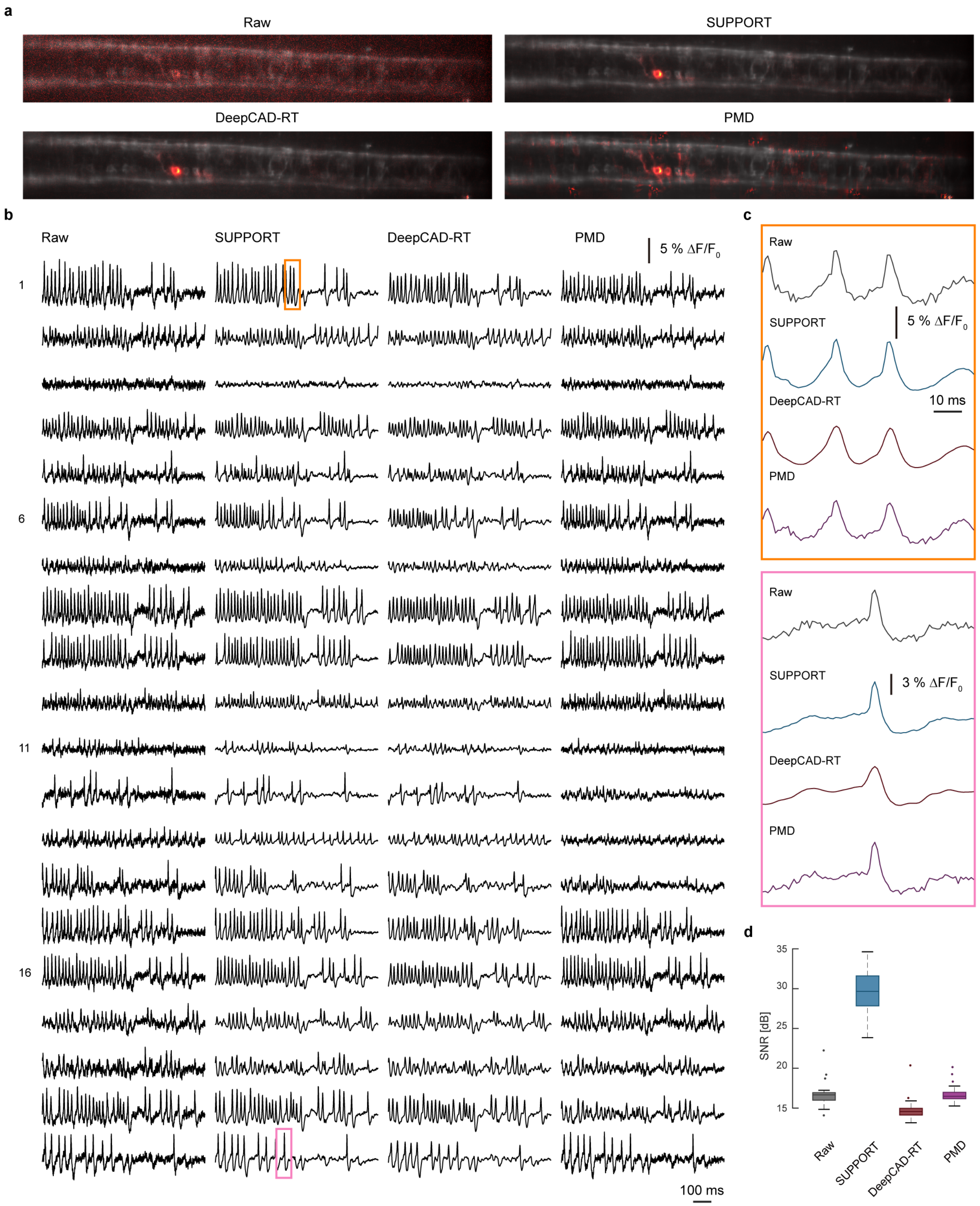

Supplementary Fig. 37: Denoising population zebrafish voltage imaging data. a, Images after baseline correction from zebrafish dataset expressing zArchon in spinal cord. **b**, Traces from 20 ROIs from raw, SUPPORT, DeepCAD-RT, and PMD. **c**, Enlarged view of traces from colored box in **b** are plotted. **d**, Box-and-whisker plot showing the signal-to-noise ratio for the extracted traces. N=20, which represents the

number of neurons.

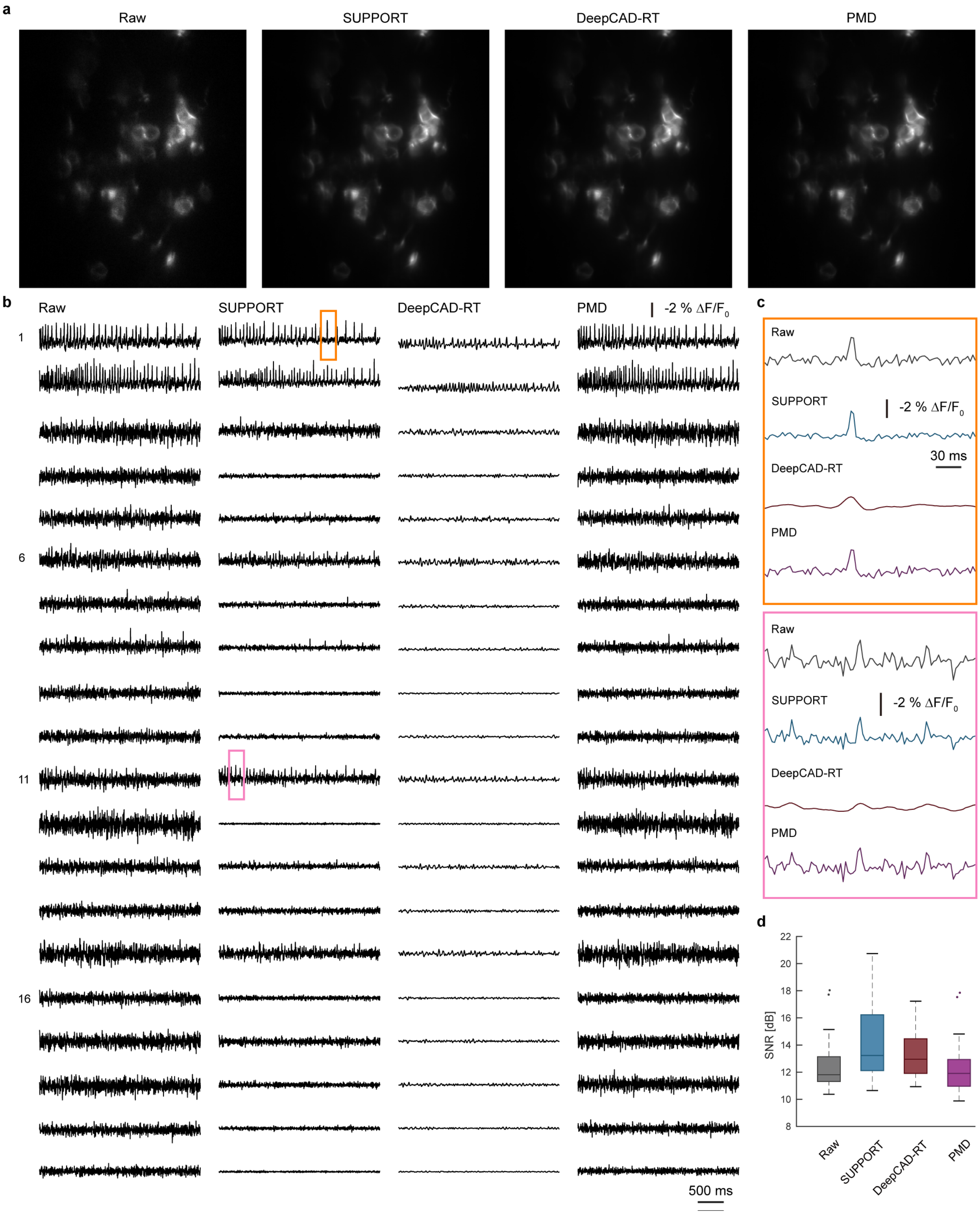

Supplementary Fig. 38: Denoising population zebrafish voltage imaging data. a, Images from zebrafish dataset expressing Voltron1 in tegmental region. **b**, Traces from 20 ROIs from raw, SUPPORT, DeepCAD-RT, and PMD. **c**, Enlarged view of traces from colored box in **b** are plotted. **d**, Box-and-whisker plot showing the signal-to-noise ratio for the extracted traces. N=31, which represents the number of neurons.

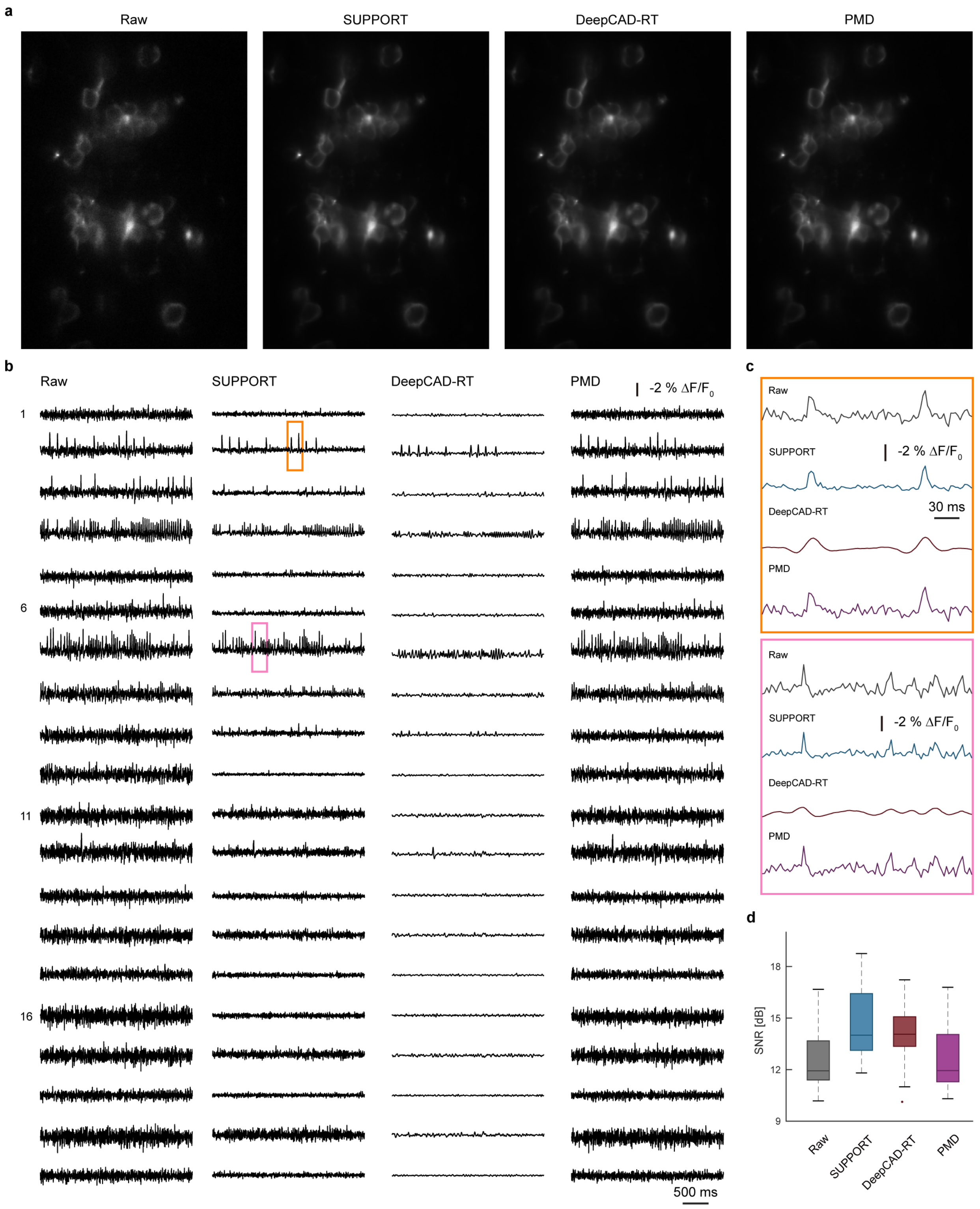

Supplementary Fig. 39: Denoising population zebrafish voltage imaging data. a, Images from zebrafish dataset expressing Voltron1 in tegmental region. **b**, Traces from 20 ROIs from raw, SUPPORT, DeepCAD-RT, and PMD. **c**, Enlarged view of traces from colored box in **b** are plotted. **d**, Box-and-whisker plot showing the signal-to-noise ratio for the extracted traces. N=28, which represents the number of neurons.

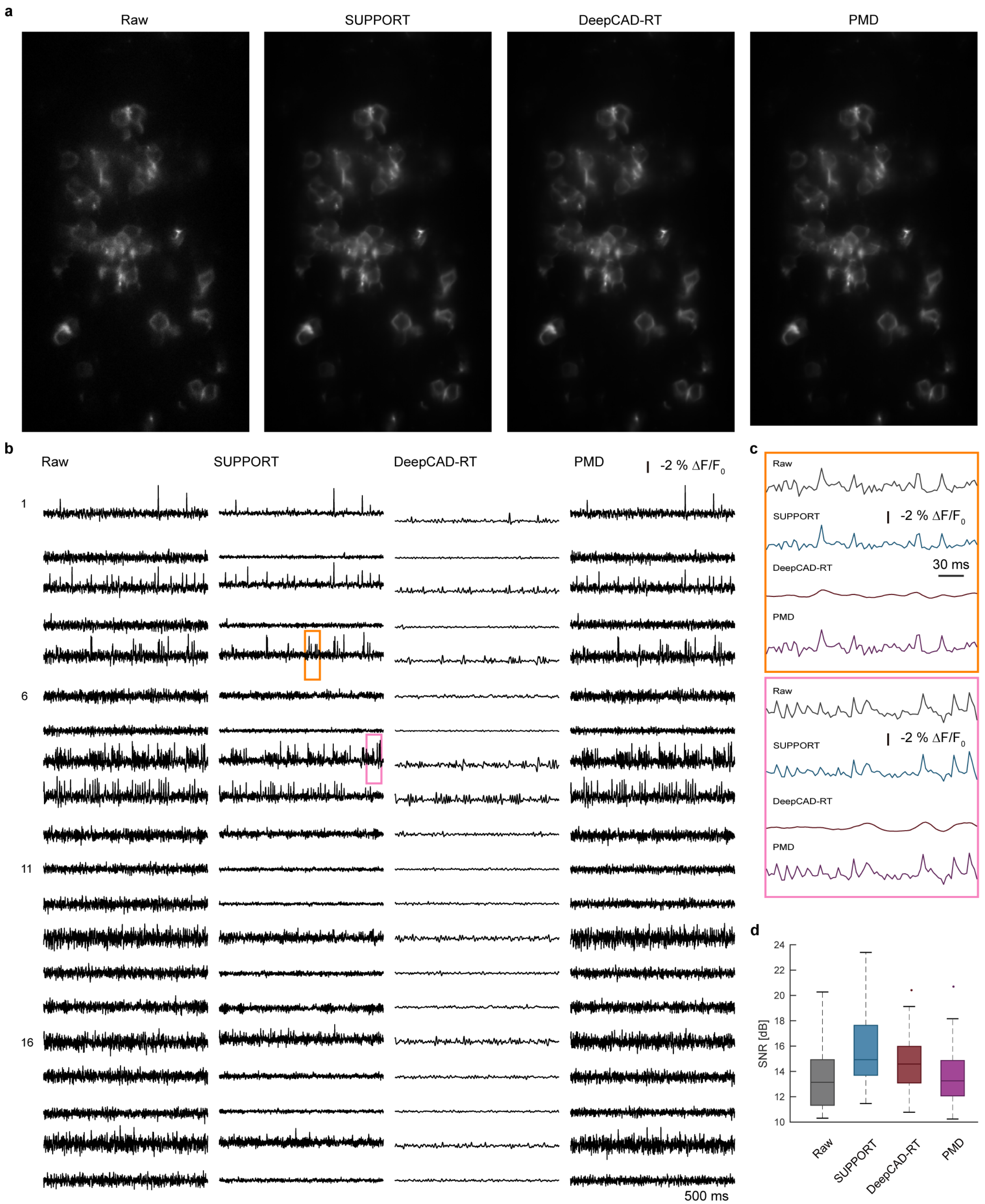

Supplementary Fig. 40: Denoising population zebrafish voltage imaging data. a, Images from zebrafish dataset expressing Voltron1 in tegmental region. **b**, Traces from 20 ROIs from raw, SUPPORT, DeepCAD-RT, and PMD. **c**, Enlarged view of traces from colored box in **b** are plotted. **d**, Box-and-whisker plot showing the signal-to-noise ratio for the extracted traces. N=41, which represents the number of neurons.

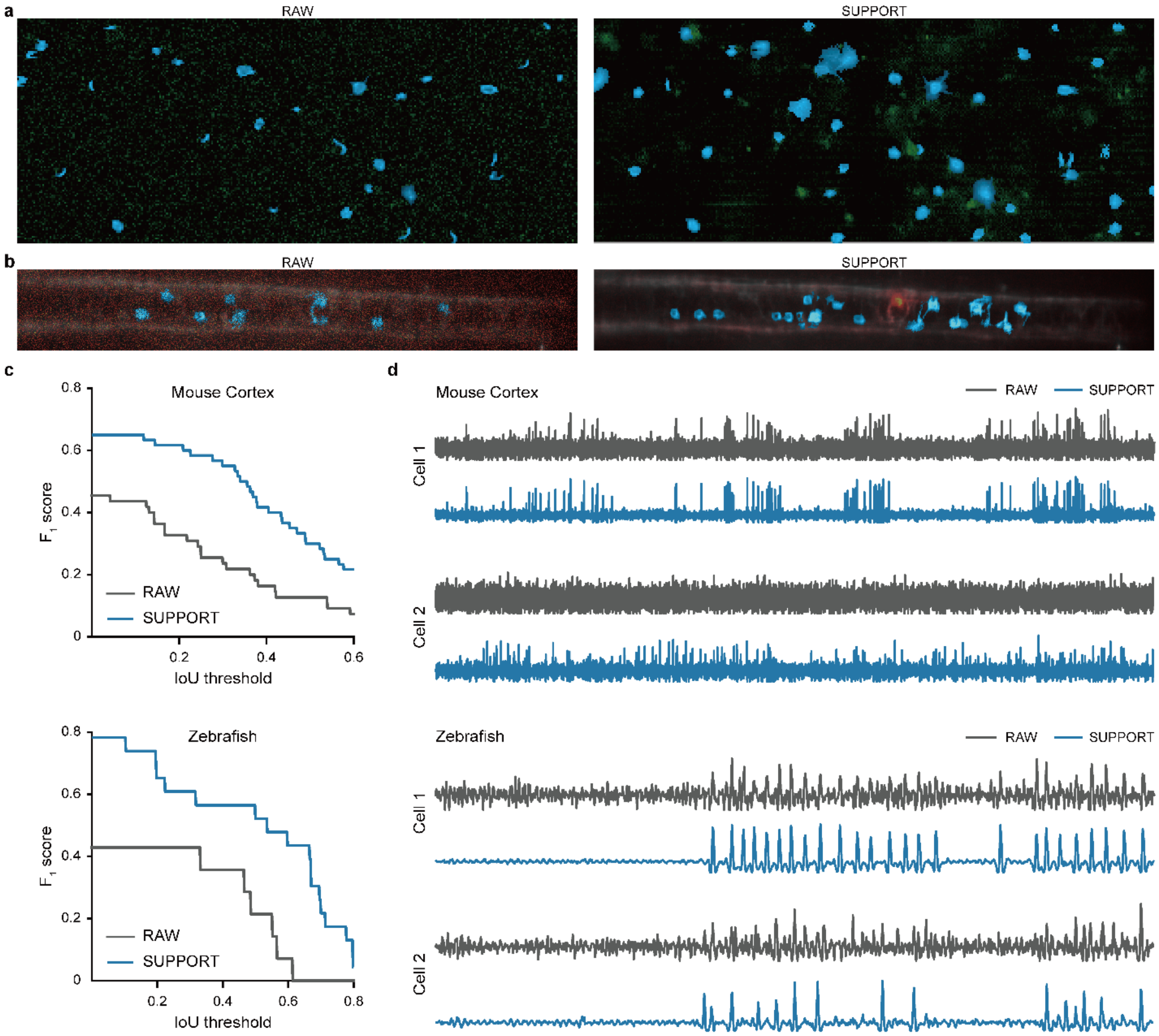

Supplementary Fig. 41: Neuronal source extraction using localNMF from population voltage imaging. **a**, Extracted neurons from raw and SUPPORT-denoised mouse videos are colored in blue and overlaid on the images of Fig. 4a. **b**, Extracted neurons from raw and SUPPORT-denoised zebrafish videos are colored in blue and overlaid on the images of Fig. 4d. **c**, F_1 scores across intersection-over-union thresholds. **d**, Extracted temporal signals from raw and SUPPORT-denoised video for two representative cells.

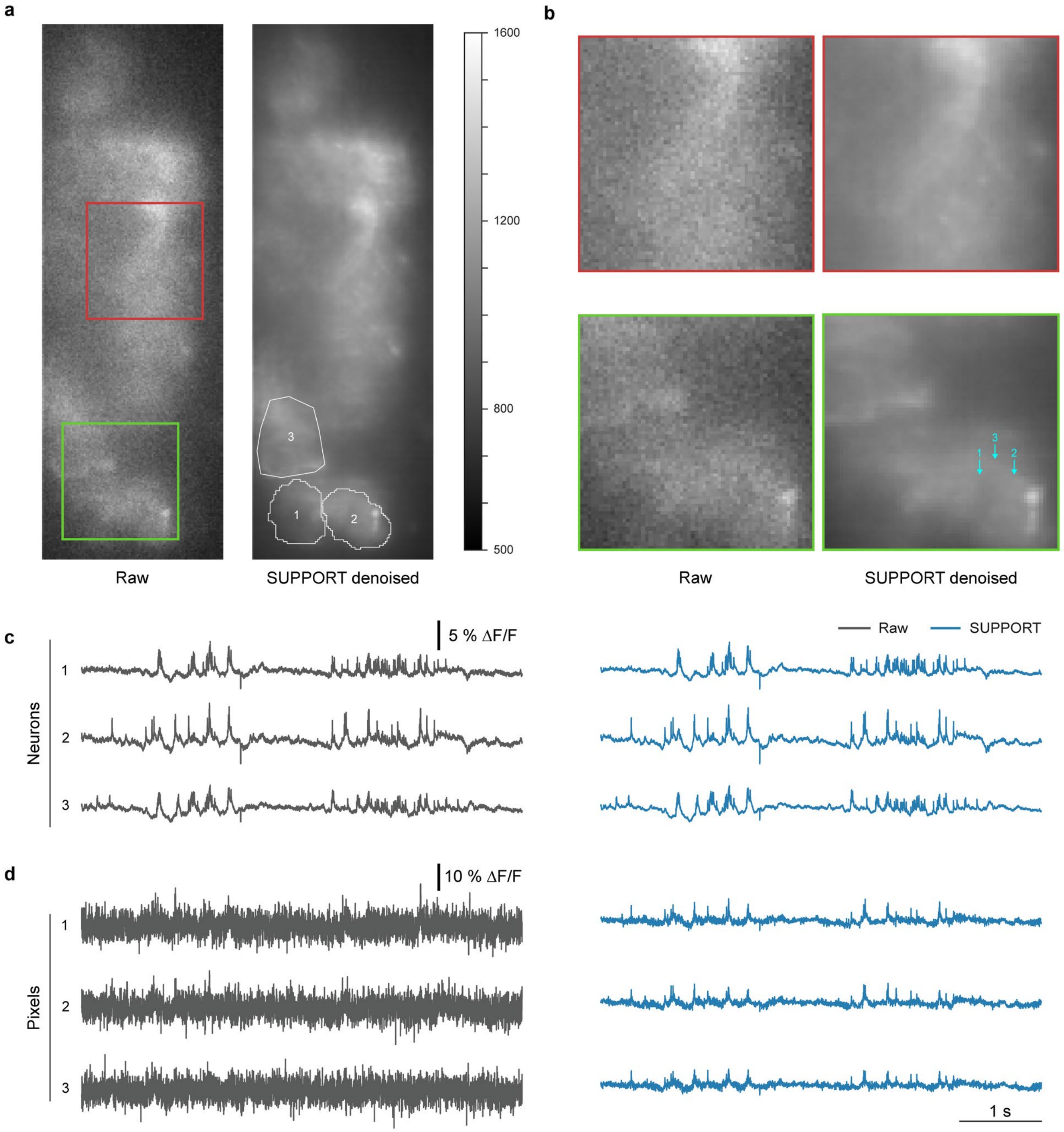

Supplementary Fig. 42: Applying SUPPORT to population voltage imaging data with paQuasAr3s indicator. a, Representative frames from raw video and SUPPORT-denoised video. paQuasAr3s-expressing mouse hippocampus CA1 was used as a dataset (Supplementary Table 1). Boundaries of three regions of interest (ROIs) are drawn with white lines. **b**, Magnified views of the boxed regions in **b**. **c**, Traces extracted from raw and SUPPORT-denoised videos for three ROIs in **a**. **d**, Single pixel traces from raw and SUPPORT-denoised video for three pixels indicated in **b**.

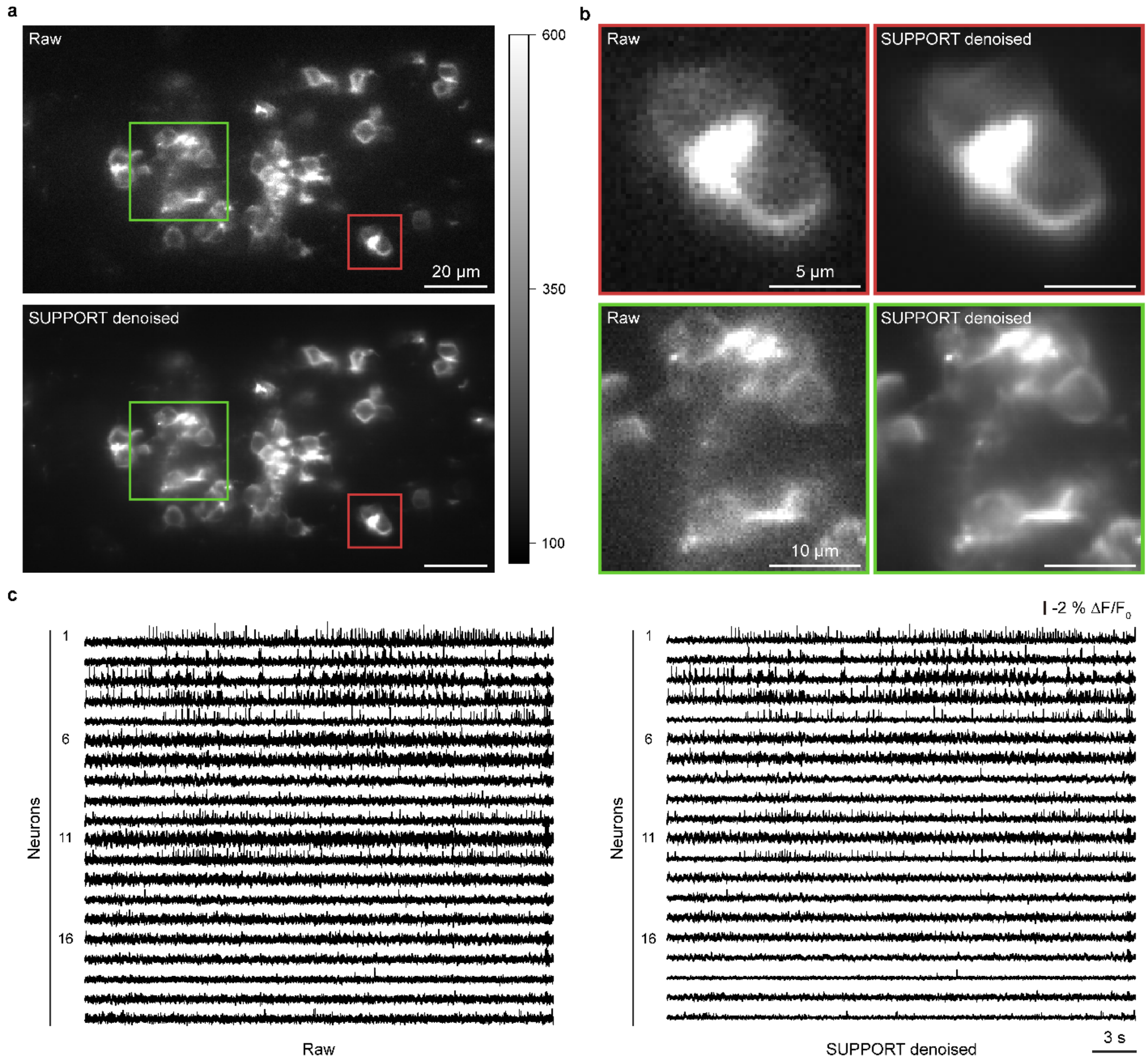

Supplementary Fig. 43: Applying SUPPORT to population voltage imaging data with Voltron1 indicator. **a**, Representative frames from raw video and SUPPORT-denoised video. Voltron1-expressing zebrafish tegmental area was used as a dataset (Supplementary Table 1). **b**, Magnified views of the boxed regions in **a**. **c**, Traces extracted from the raw and SUPPORT-denoised video for 20 neurons. Left: From the raw video. Right: From SUPPORT-denoised video.

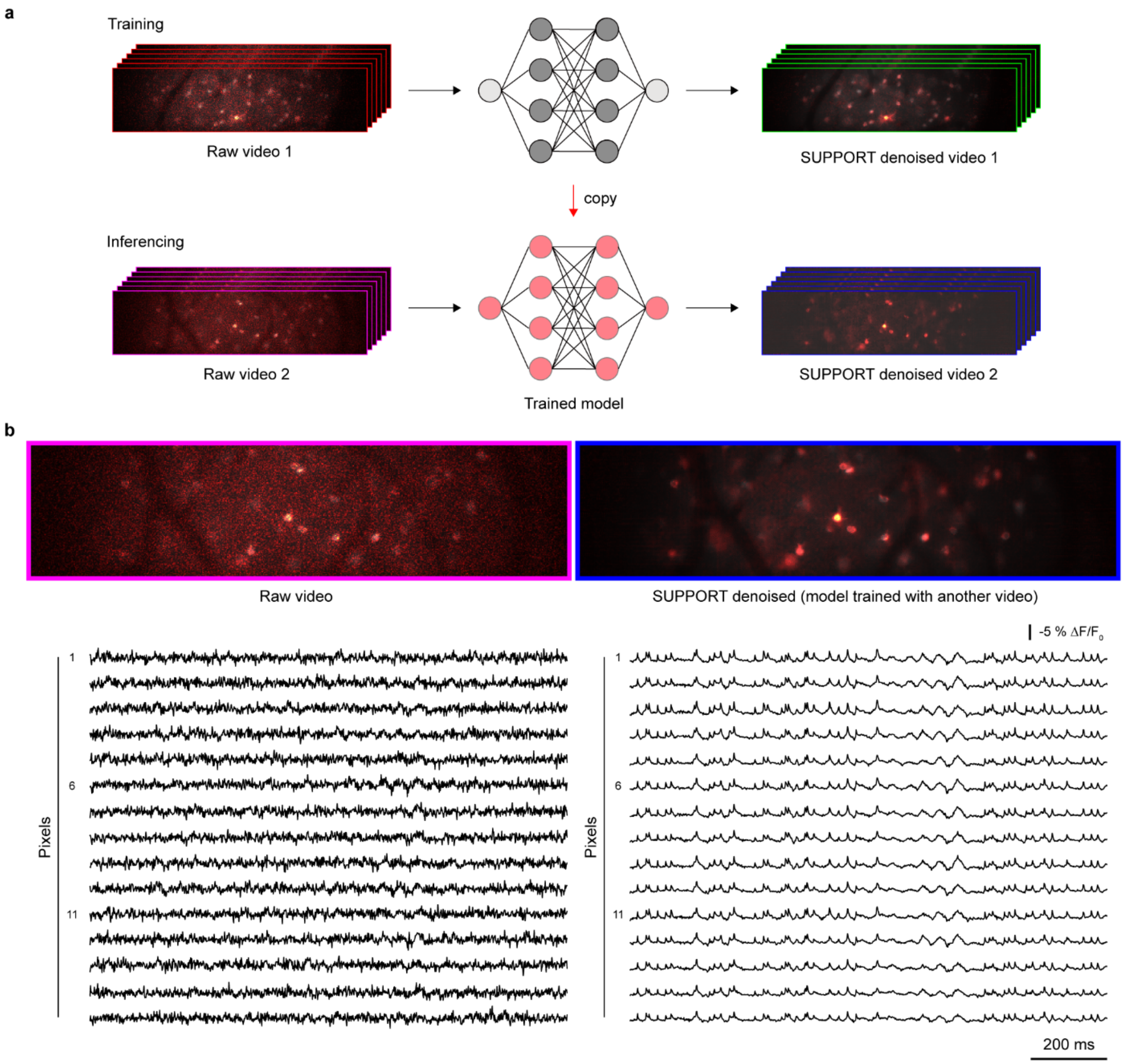

Supplementary Fig. 44: Network generalization for population voltage imaging data. a, Ability to generalize SUPPORT for denoising unseen dataset. Top: SUPPORT is first trained with raw video 1. Bottom: Trained SUPPORT network is used for denoising raw video 2, which is recorded from different fields of view and time. **b**, SUPPORT was able to denoise the unseen dataset without additional fine-tuning. Top: A representative frame of raw video and SUPPORT-denoised video, for which the raw data was never shown to SUPPORT on training. Bottom left: Single pixel traces inside one neuron from the raw video. Bottom right: Traces from SUPPORT-denoised video. This shows that the traces from SUPPORT-denoised video are highly correlated.

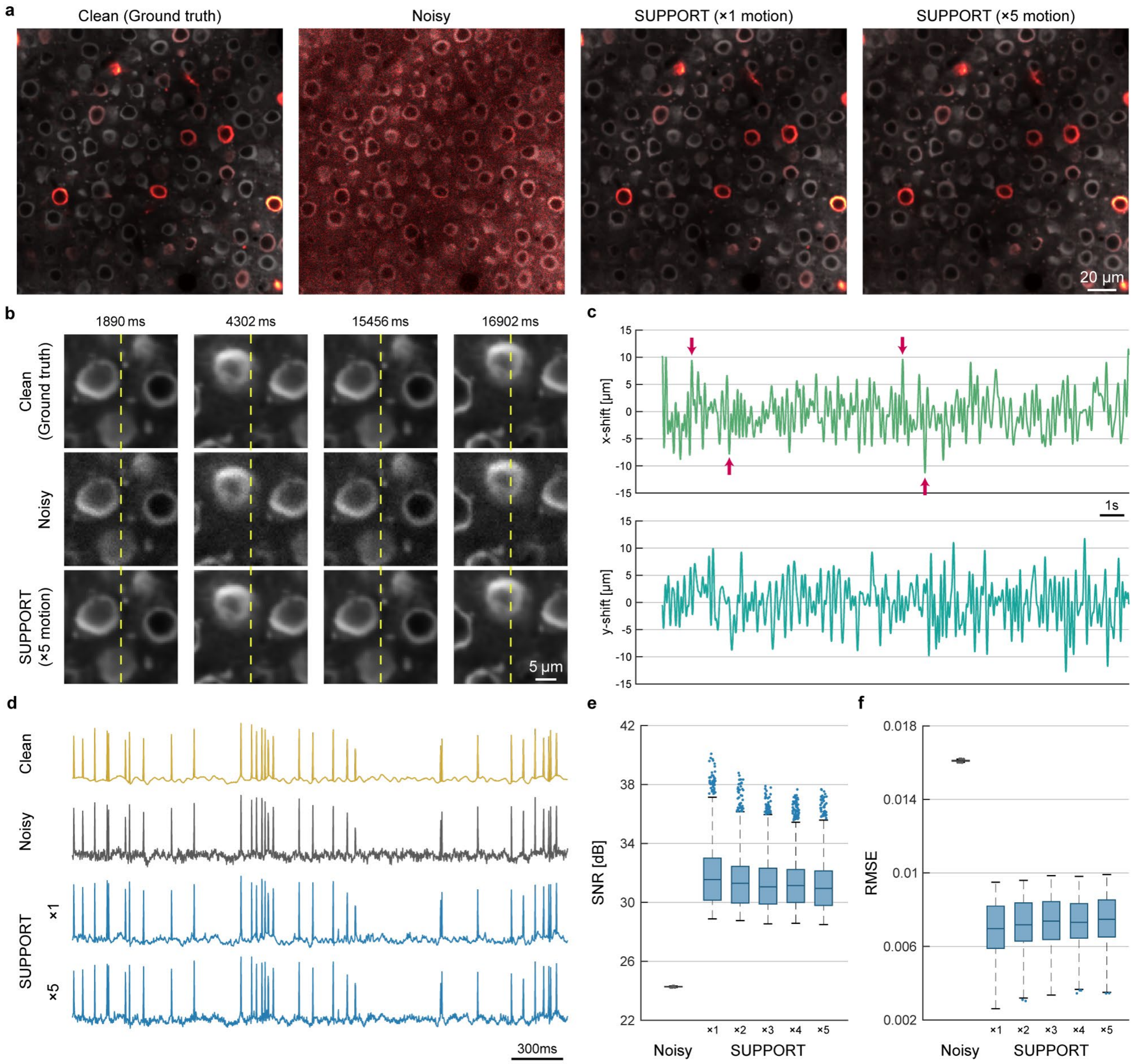

Supplementary Fig. 45: Denoising simulated voltage imaging data with motion in the x and y directions. **a**, Representative frames of clean video, noisy video, and SUPPORT-denoised videos with motion after baseline correction. ×5 indicates a five times higher motion compared to ×1. **b**, Spatially expanded view of representative frames at the timings indicated by red arrows in **c**. From left to right: frames at 1890ms, 4302ms, 15456ms, and 16902ms. From top to bottom: clean video, noisy video, and SUPPORT-denoised video with motion. **c**, Line plot showing x and y-direction motions in the micrometer scale. **d**, Traces extracted from a single cell of the clean video, noisy video, and SUPPORT-denoised video with motion. **e**, Box-and-whisker plot showing SNR before and after denoising data with motion. N=1440, which represents the number of planes along the t-axis. **f**, Box-and-whisker plot showing RMSE before and after denoising data with motion. N=1440, which represents the number of planes along the t-axis.

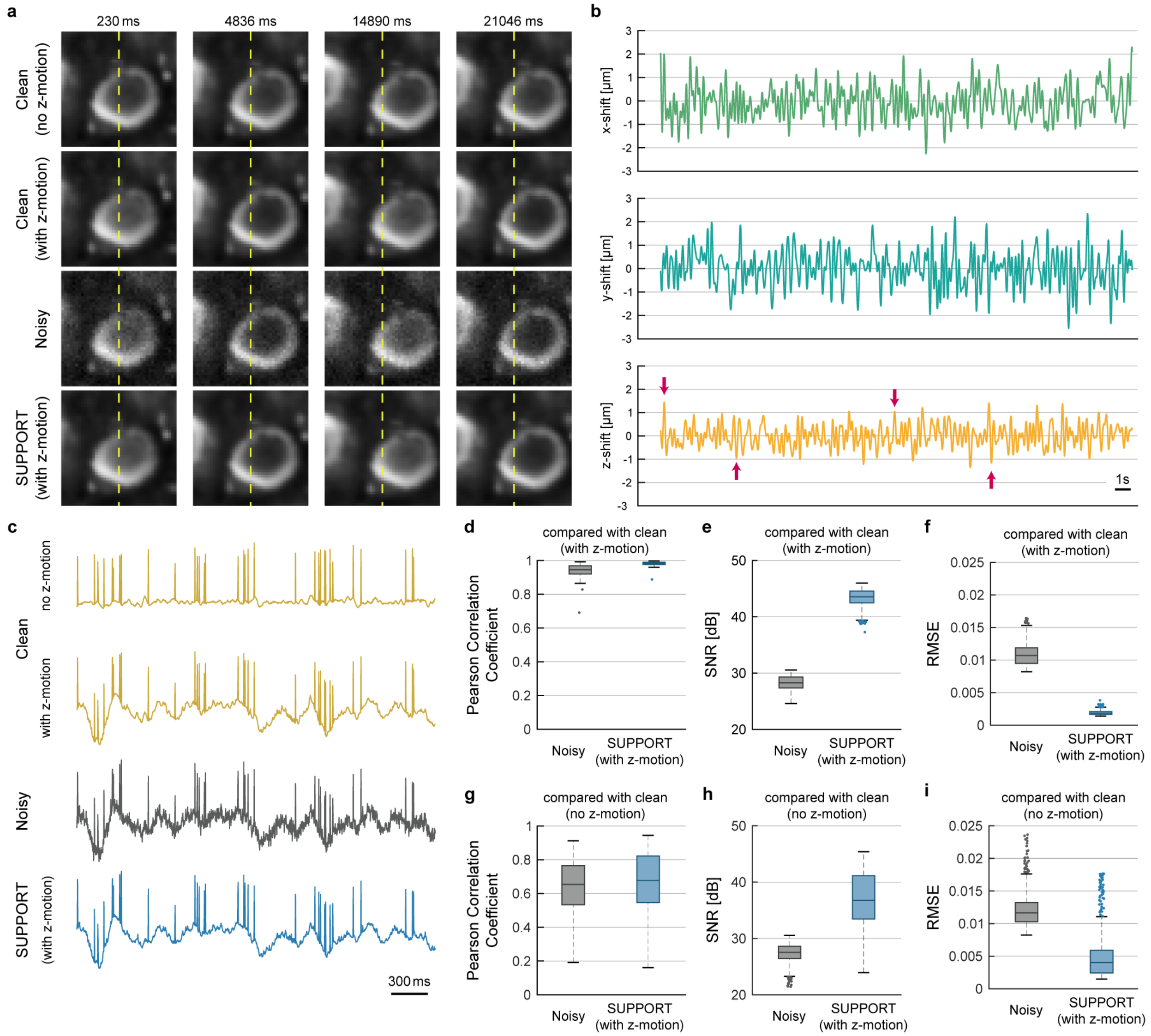

Supplementary Fig. 46: Denoising simulated voltage imaging data with motion in the x, y, and z directions. a, Representative frames indicated with red arrows in **b**. From left to right: frames at 230ms, 4836ms, 14890ms, and 21046ms. From top to bottom: clean video without and with z-motion, noisy video, and SUPPORT-denoised video with z-motion. **b**, line plot showing x, y, and z-direction motions in the micrometer scale. **c**, Traces extracted from a single cell of the clean video without and with z-motion, noisy video, and SUPPORT-denoised video with z-motion. **d**, Box-and-whisker plot showing Pearson correlation coefficient before and after denoising data with z-motion compared with clean data with z-motion. N=102, which represents the number of neurons. **e**, Box-and-whisker plot showing SNR before and after denoising data with z-motion compared with clean data with z-motion. N=1470, which represents the number of planes along the t-axis. **f**, Box-and-whisker plot showing RMSE before and after denoising data with z-motion compared with clean data with z-motion. N=1470, which represents the number of planes along the t-axis. **g**, Box-and-whisker plot showing Pearson correlation coefficient before and after denoising data with z-motion compared with clean data without z-motion. N=102, which represents the number of neurons. **h**, Box-and-whisker plot showing SNR before and after denoising data with z-motion compared with clean

data without z-motion. N=1470, which represents the number of planes along the t-axis. **i**, Box-and-whisker plot showing RMSE before and after denoising data with z-motion compared with clean data without z-motion. N=1470, which represents the number of planes along the t-axis.

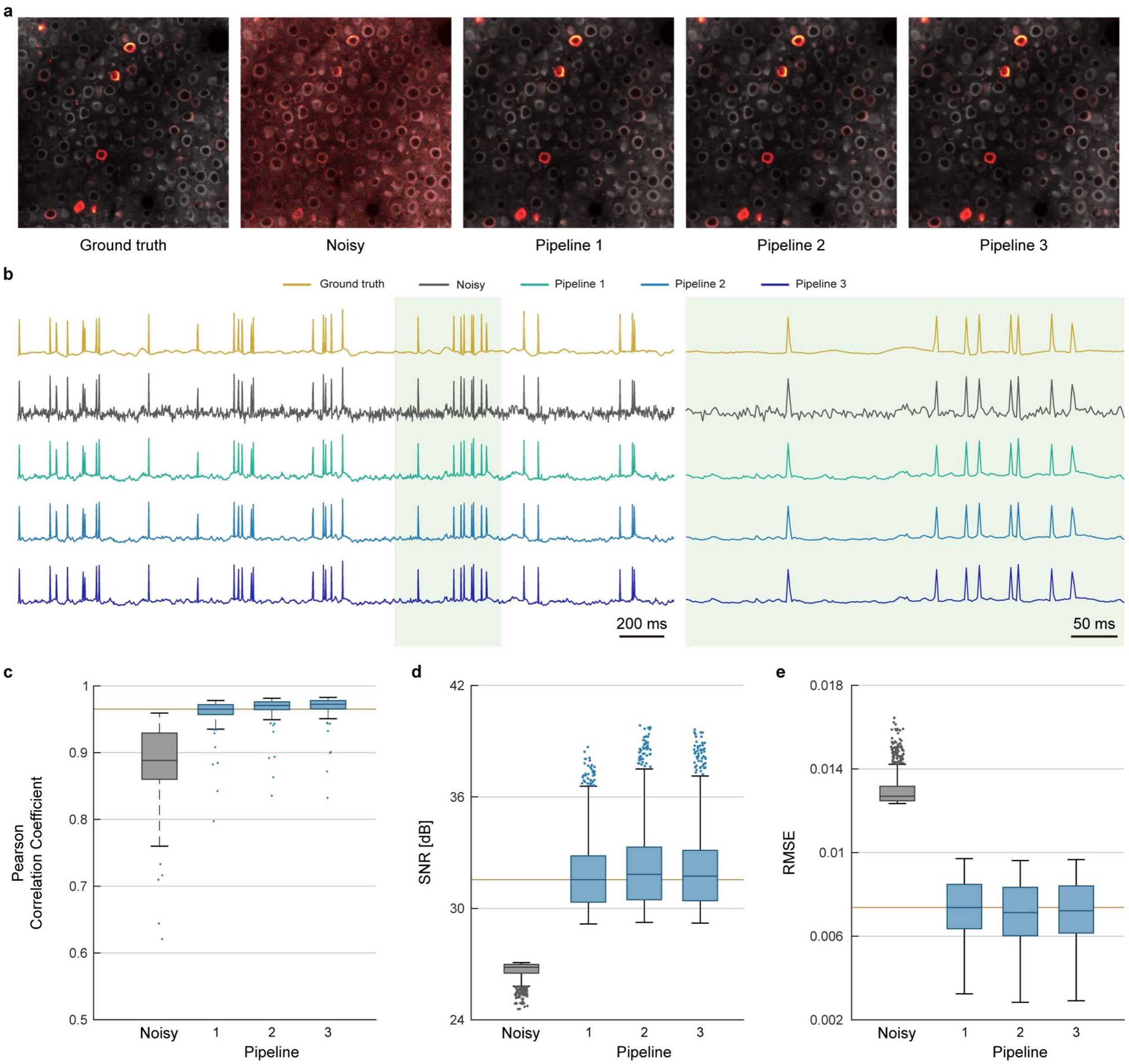

Supplementary Fig. 47: Performance comparison between SUPPORT pipelines using simulated data. **a**, Representative frames of clean video, noisy video, and SUPPORT-denoised videos acquired with different pipelines after baseline correction. Simulated data was used. Three different pipelines were used in which the difference was the order of operations between motion correction, denoising, and photobleaching correction. Pipeline 1: SUPPORT denoising followed by motion correction followed by a photobleaching correction. Pipeline 2: motion correction followed by SUPPORT denoising followed by a photobleaching correction. Pipeline 3: motion correction followed by a photobleaching correction followed by SUPPORT denoising. **b**, Traces extracted from a single cell of the clean video, noisy video, and SUPPORT-denoised videos. Temporally expanded traces from the green area on the left are shown on the right. **c**, Box-and-whisker plot showing the Pearson correlation coefficients of extracted traces from the ROIs before and after denoising data with different pipelines. N=102, which represents the number of neurons. **d**, Box-and-whisker plot showing the frame-wise SNR before and after denoising data with different pipelines. N=1500, which represents the number of planes along the t-axis. **e**, Box-and-whisker plot showing the frame-wise RMSE before and after denoising data with different pipelines. N=1470, which

represents the number of planes along the t-axis.

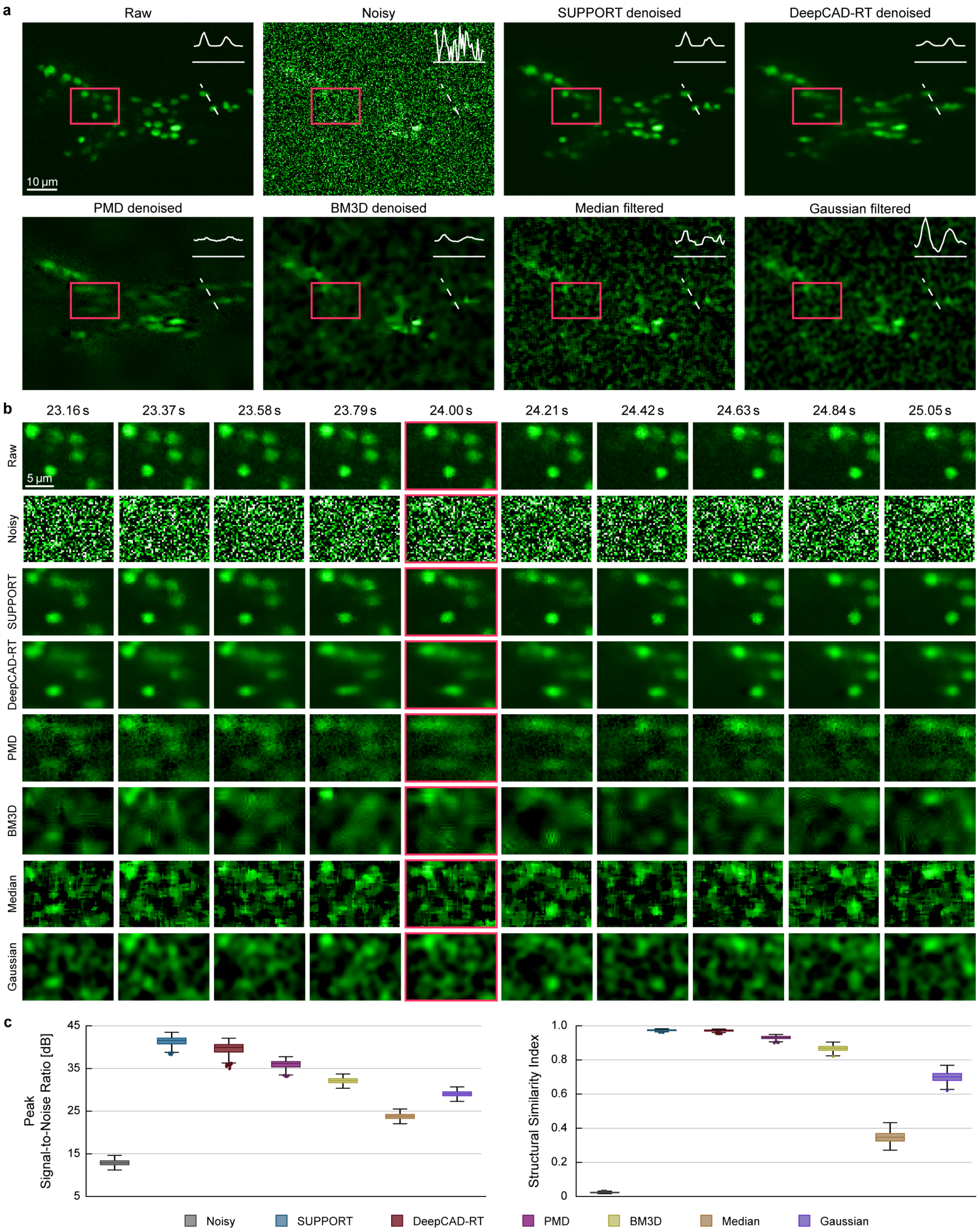

Supplementary Fig. 48: Denoising performance compared with other 2D denoising methods on freely

moving *C.elegans* imaging data. a, Raw, noisy, and denoised images of freely moving *C. elegans* expressing NLS-mCherry. Inset shows the intensity profile along the dashed line. **b**, Magnified views of the red boxed region in **a** at consecutive neighboring time points. From top to bottom: raw, noisy, SUPPORT-denoised, DeepCAD-RT-denoised, PMD-denoised, BM3D-denoised, median filtered, and gaussian filtered. **c**, Peak signal-to-noise ratio (PSNR) and Structural similarity index between raw and denoised data. N=531, which represents the number of planes along the t-axis.

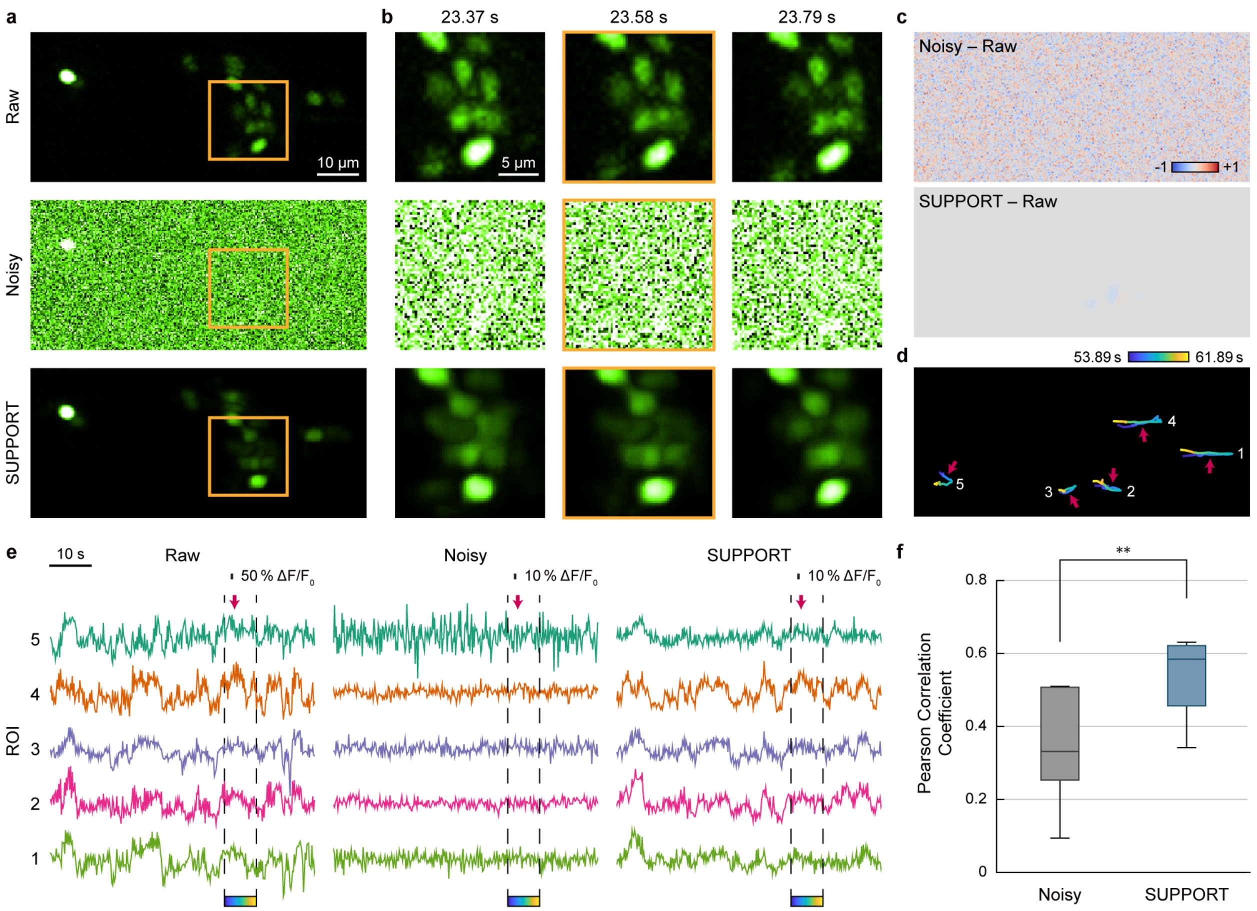

Supplementary Fig. 49: Denoising freely moving *C.elegans* calcium imaging data. a, Calcium imaging data of freely moving *C. elegans* expressing NLS-YC2. From top to bottom: Raw, Noisy, and SUPPORT-denoised data. **b**, Magnified views of the boxed regions in a at consecutive neighboring time points. **c**, Pixel-wise difference acquired by subtracting raw data from noisy and denoised data. **d**, Location of centroid over time is color-coded for five neuronal ROIs. **e**, Traces for 5 ROIs from raw, Noisy, and SUPPORT-denoised data. Area between two dashed lines corresponds to the temporal area visualized in **d**. Location indicated by red arrows corresponds to the red arrows in **d**. **f**, Box-and-whisker plot showing Pearson correlation coefficients between traces from Noisy and SUPPORT-denoised data and traces from raw data. Two-sided paired-sample t-test is used. N=5, which represents the number of neurons (**: p-value<0.01).

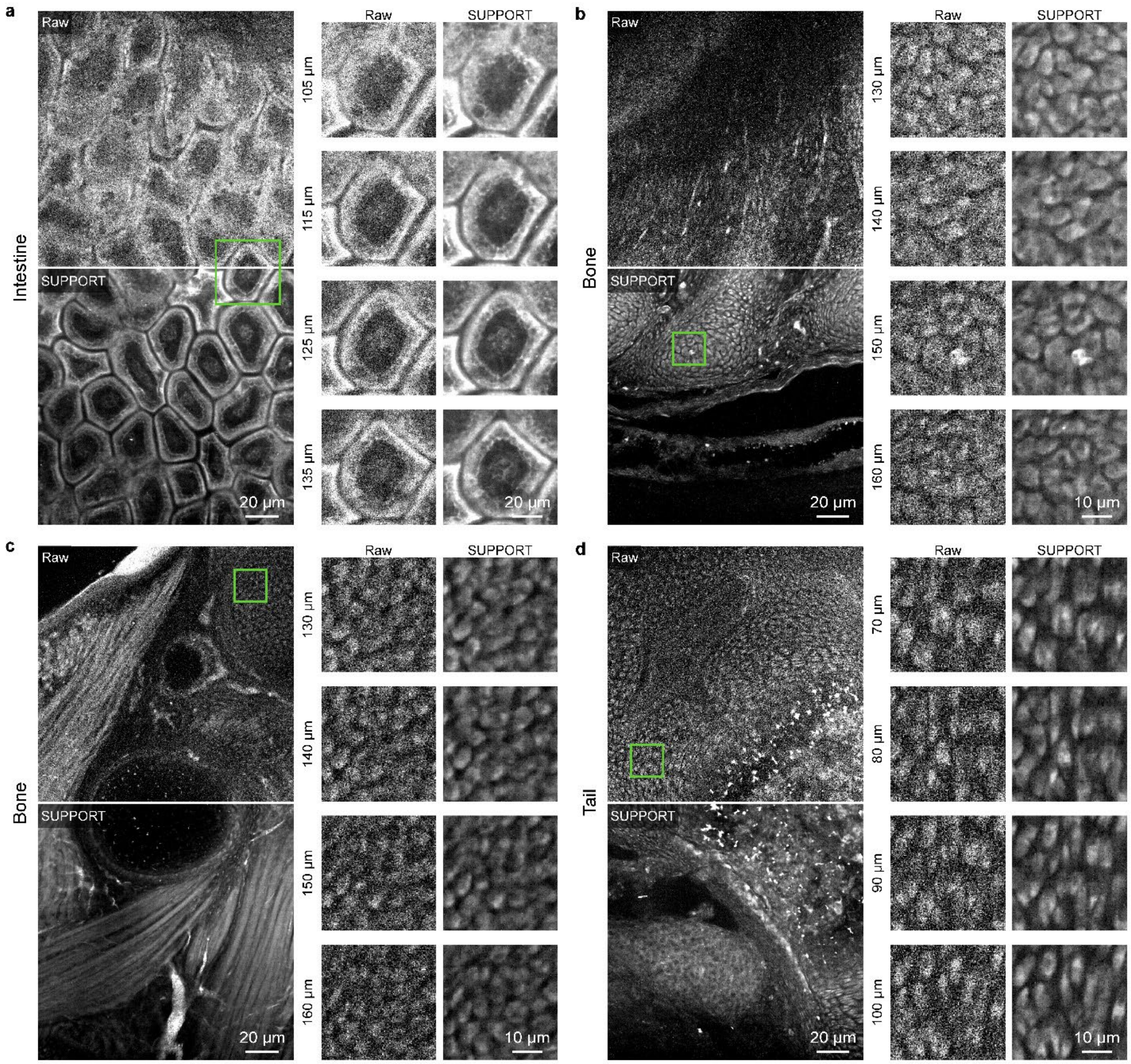

Supplementary Fig. 50: Applying SUPPORT to volumetric structural images of mouse embryos acquired with expansion microscopy. a–d, Left: A frame at depth 125 µm from raw data (top) and SUPPORT-denoised data (bottom). Right: Expanded view of green box on the left at multiple depths. **a,** Intestine. **b-c**, Bone. **d**, Tail. Biological data shown are representative from n=5 stacks.

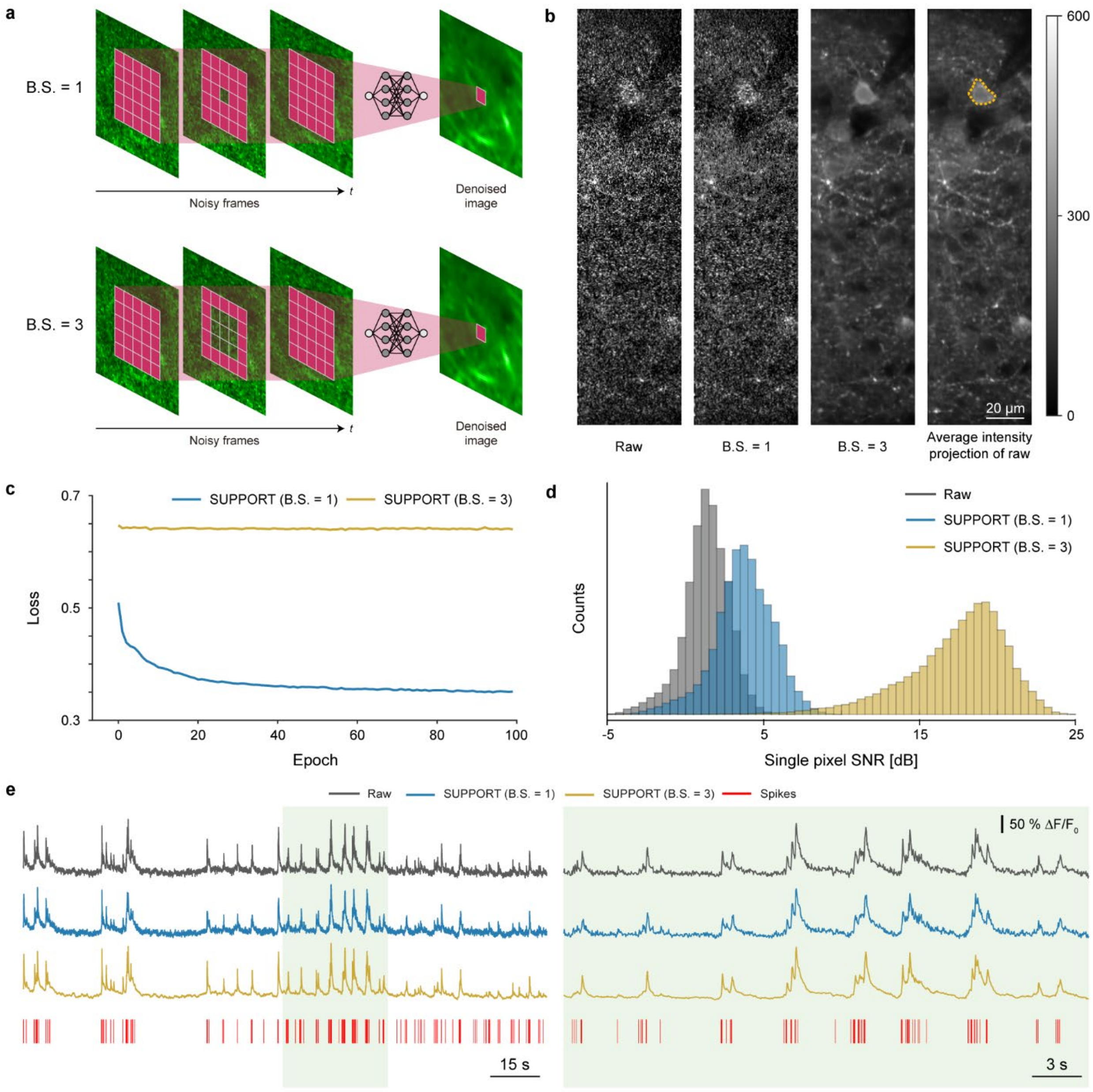

Supplementary Fig. 51: Increasing the size of the blind spot to denoise the data with structured noise. **a**, Receptive fields with different sizes of the blind spot. Top: Blind spot size of 1. Bottom: Blind spot size of 3. The center frame of noisy frames is the current frame to be denoised. **b**, Representative frames of raw and SUPPORT-denoised data with structural noise. Raw data were recorded from a jGCaMP8f-expressing mouse cortex L2/3 (Supplementary Table 1). From left to right: Raw data, SUPPORT-denoised data with blind spot sizes of 1 and 3, and the average intensity projection of the raw data. The boundary of the region of interest (ROI) is drawn with a yellow dotted line. **c**, Training loss curve of SUPPORT with blind spot sizes of 1 and 3. With a blind spot size of 1, SUPPORT learned to predict the structured noise, and training loss was significantly lower than for a blind spot size of 3. **d**, Histogram of single pixel signal-to-noise ratio (SNR) from raw data, SUPPORT-denoised data with blind spot sizes of 1 and 3. While a blind spot size of 3 significantly increased the SNR, a size of 1 only slightly increased the SNR. **e**, Traces extracted from the raw data, SUPPORT-denoised data with blind spot sizes of 1 and 3 for the ROI in **b**, and spikes (action

potentials) detected from simultaneous electrophysiological recording. Temporally expanded traces from the green area of the left are shown on the right.

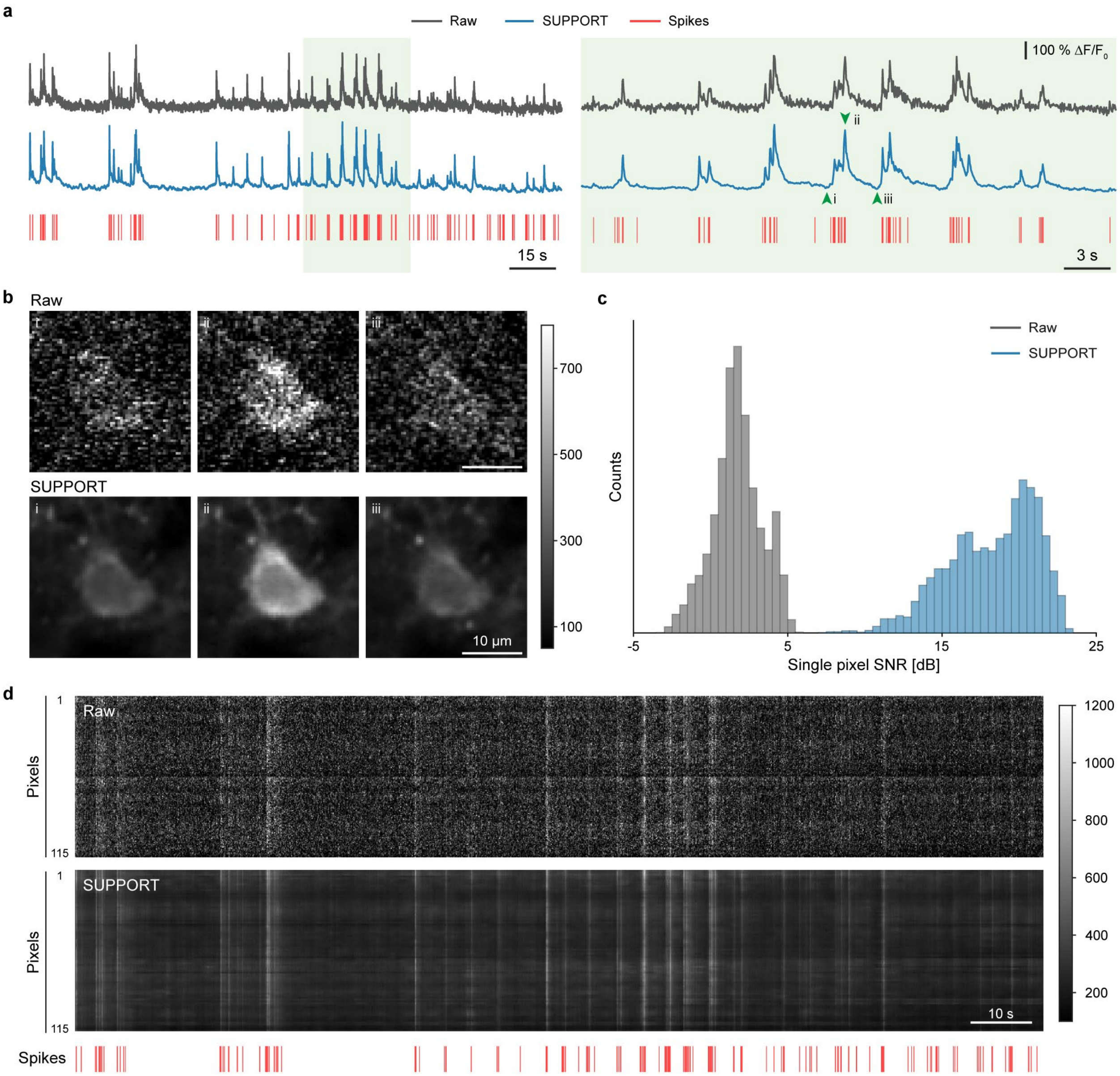

Supplementary Fig. 52: Applying SUPPORT to simultaneous calcium imaging and electrophysiological recording data with jGCaMP8f indicator. a, Traces extracted from the cytoplasmic area of raw and SUPPORT-denoised video. Raw data were recorded from a jGCaMP8f-expressing mouse cortex L2/3 (Supplementary Table 1). Spikes (action potentials) detected from simultaneous electrophysiological recordings are drawn at the bottom. Temporally expanded traces from the green area of the left are shown on the right. **b**, Representative frames of the raw and SUPPORT-denoised video marked with green arrows on **a**. Top: From the raw video. Bottom: From the SUPPORT-denoised video. **c**, Histogram of single pixel signal-to-noise ratio (SNR) from the raw video and SUPPORT-denoised video. **d**, Traces for pixels in the cell from raw and denoised data. Each row corresponds to a signal of each pixel. Spikes detected from simultaneous electrophysiological recordings are plotted underneath.

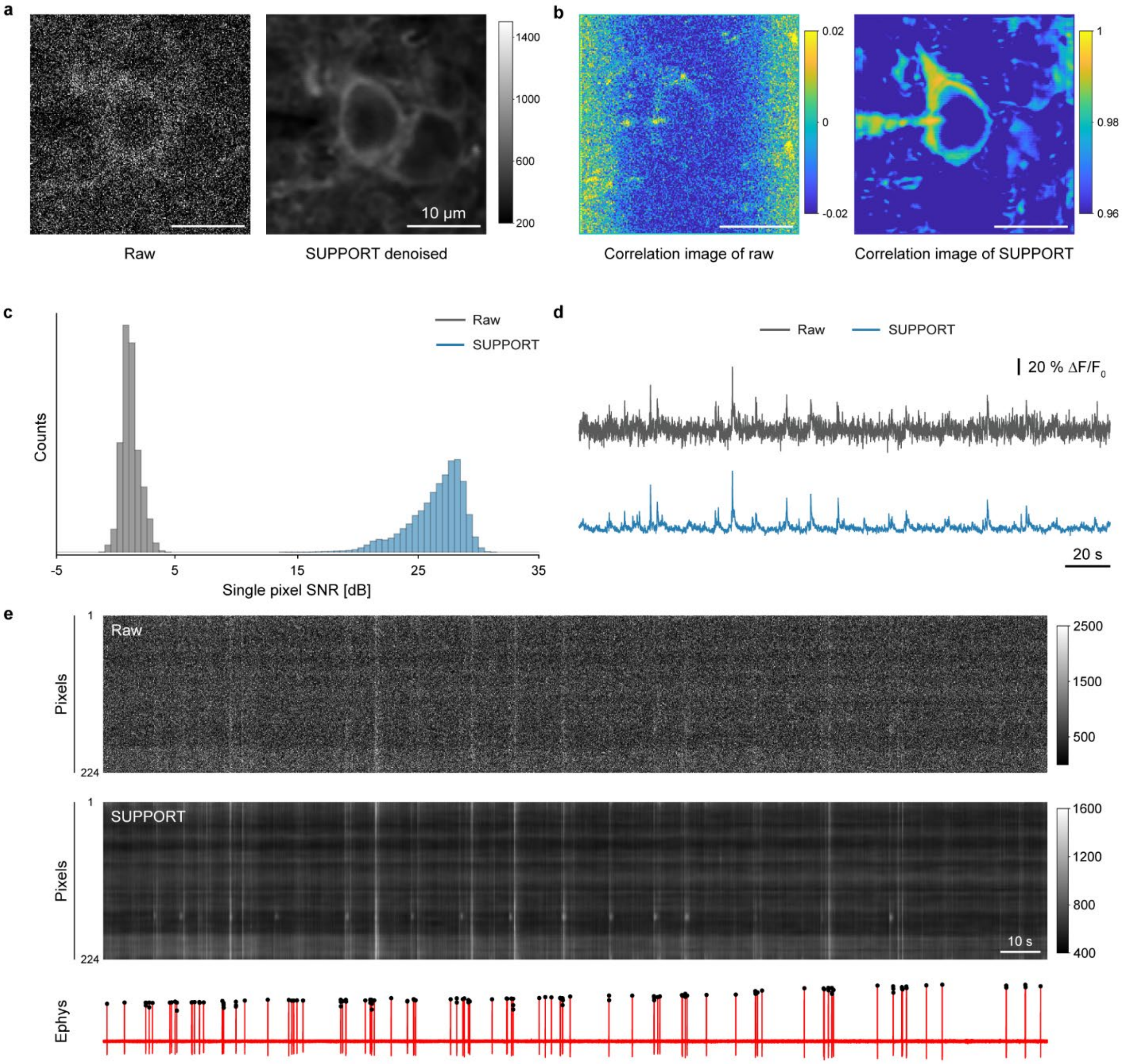

Supplementary Fig. 53: Applying SUPPORT to simultaneous calcium imaging and electrophysiological recording data with GCaMP6f indicator. **a**, Representative frames of raw video and SUPPORT-denoised video. Raw data were recorded from a GCaMP6f-expressing mouse cortex V1 (Supplementary Table 1). **b**, Correlation images of raw video and SUPPORT-denoised video. The correlation image for each pixel is the average of the correlation coefficients between the signal of that pixel and the signals of neighboring pixels. **c**, Histogram of single pixel signal-to-noise ratio (SNR) from raw and SUPPORT-denoised videos. **d**, Traces extracted from the ROI of raw and SUPPORT-denoised videos. The ROI contains 100 pixels inside the cytoplasm. **e**, Traces for pixels in the cell from raw and SUPPORT-denoised video. Each row corresponds to a signal of each pixel. Simultaneous electrophysiological recording is plotted underneath, with black dots for detected spikes.

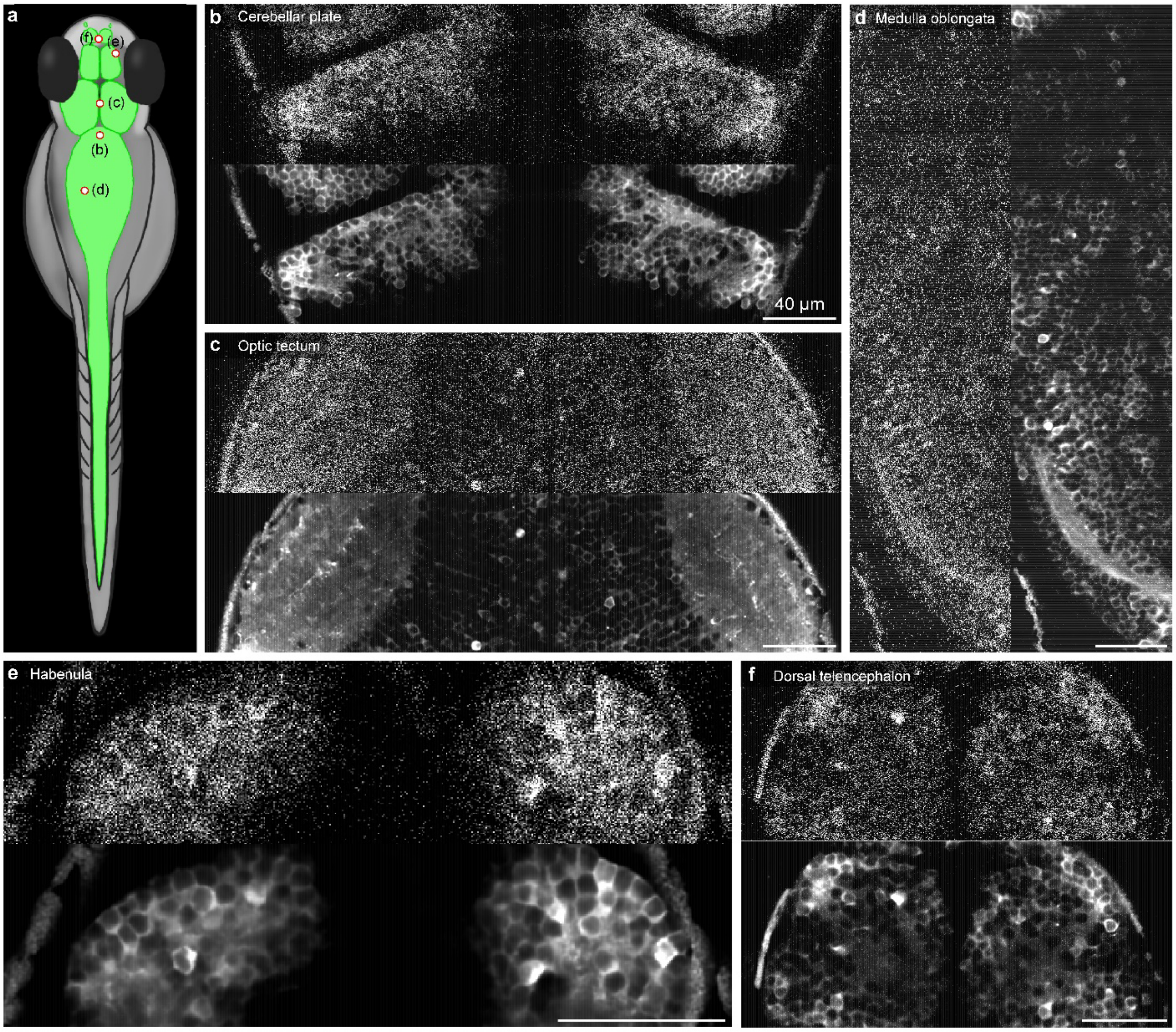

Supplementary Fig. 54: SUPPORT denoises calcium imaging in larval zebrafish. a, Larval zebrafish expressing GCaMP7a calcium indicator under control of huc promoter. **b–f**, Top left: Representative frames of raw video. Bottom right: Representative frame of SUPPORT-denoised video. **b**, Cerebellar plate. **c**, Optic tectum. **d**, Medulla oblongata. **e**, Habenula. **f**, Dorsal telencephalon.

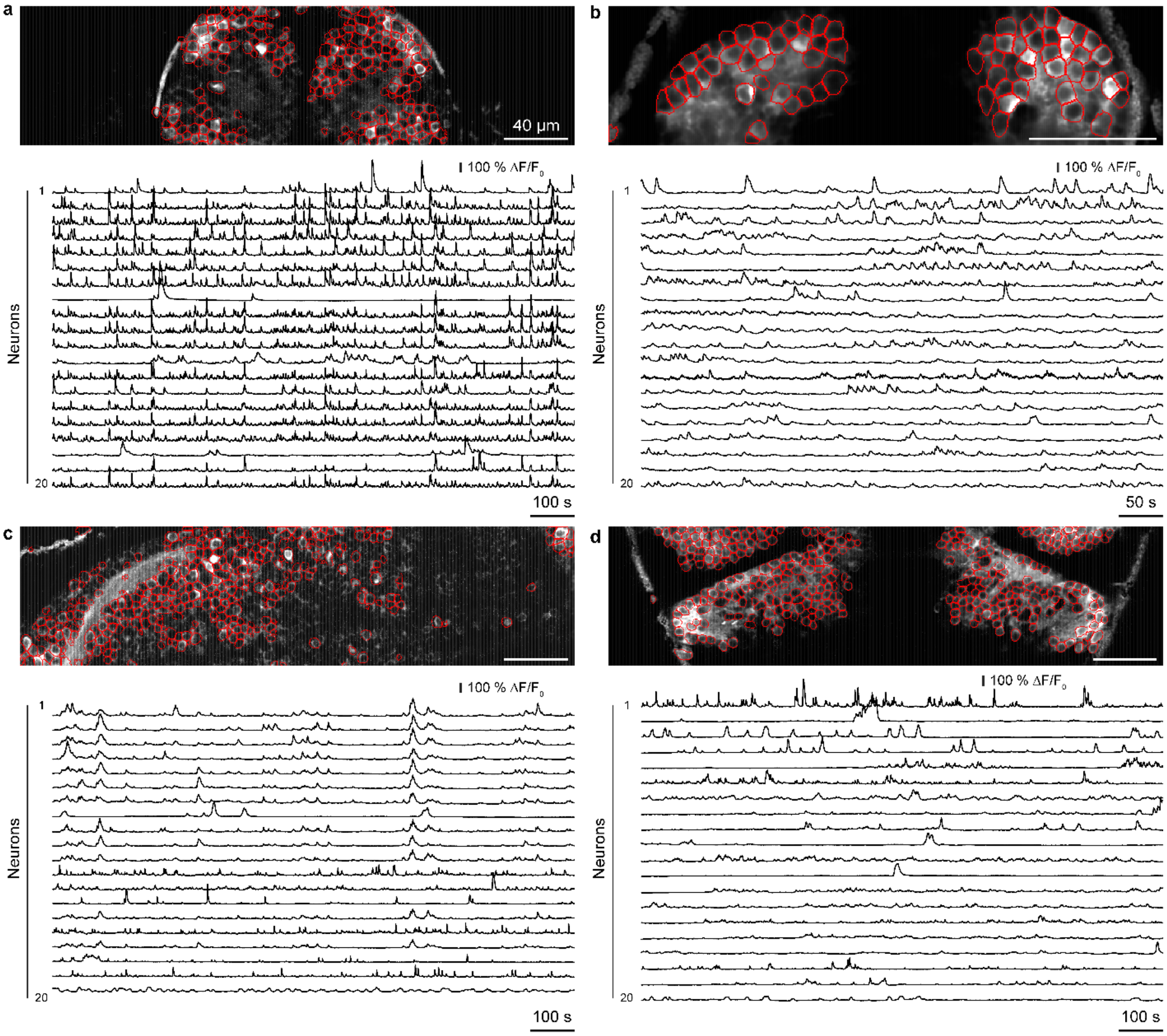

Supplementary Fig. 55: SUPPORT denoising enables single frame cell detection. a–d, Top: Neurons extracted from a single frame of SUPPORT-denoised video. Bottom: Representative calcium traces of 20 neurons. **a**, Dorsal telencephalon. **b**, Habenula. **c**, Medulla oblongata. **d**, Cerebellar plate.

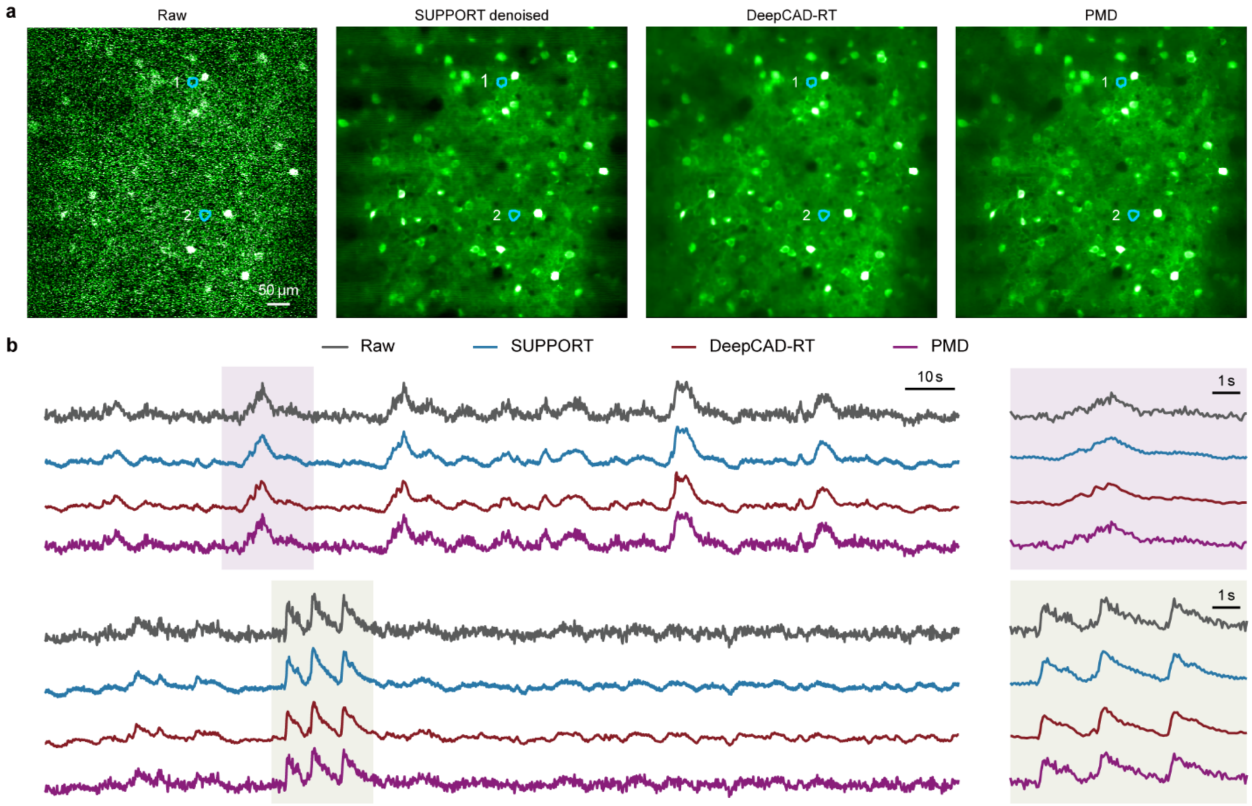

Supplementary Fig. 56: Applying SUPPORT to Neurofinder calcium imaging dataset. a, Representative frame of raw, SUPPORT, DeepCAD-RT, and PMD denoised. **b**, Two neuronal traces from raw and denoised data. Traces for the smaller temporal region are plotted on the right.

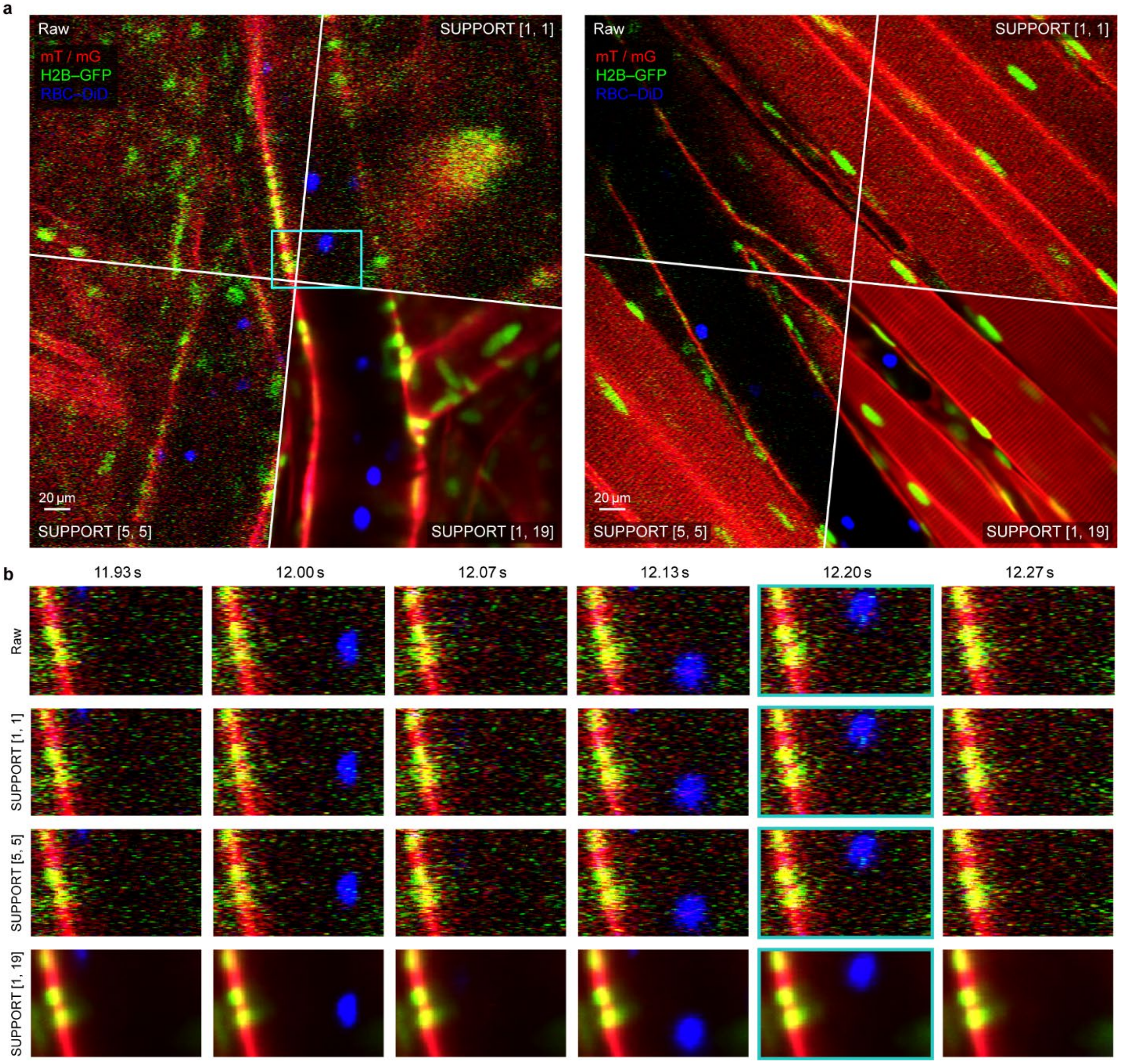

Supplementary Fig. 57: Applying SUPPORT to multi-color in vivo imaging of mouse organs. a, Confocal image of ear skin and muscle of H2B-GFP (green) and mTmG (red) mice with red blood cells fluorescently labelled by far-red fluorophore DiD (blue). Left: Ear skin, Right: muscle. Comparisons of raw and SUPPORT-denoised data with blind spot sizes of 1×1, 5×5, and 1×19 are displayed. **b**, Expanded view of cyan box in **a** with consecutive frames are displayed. Biological data shown are representative from n=5 experiments.

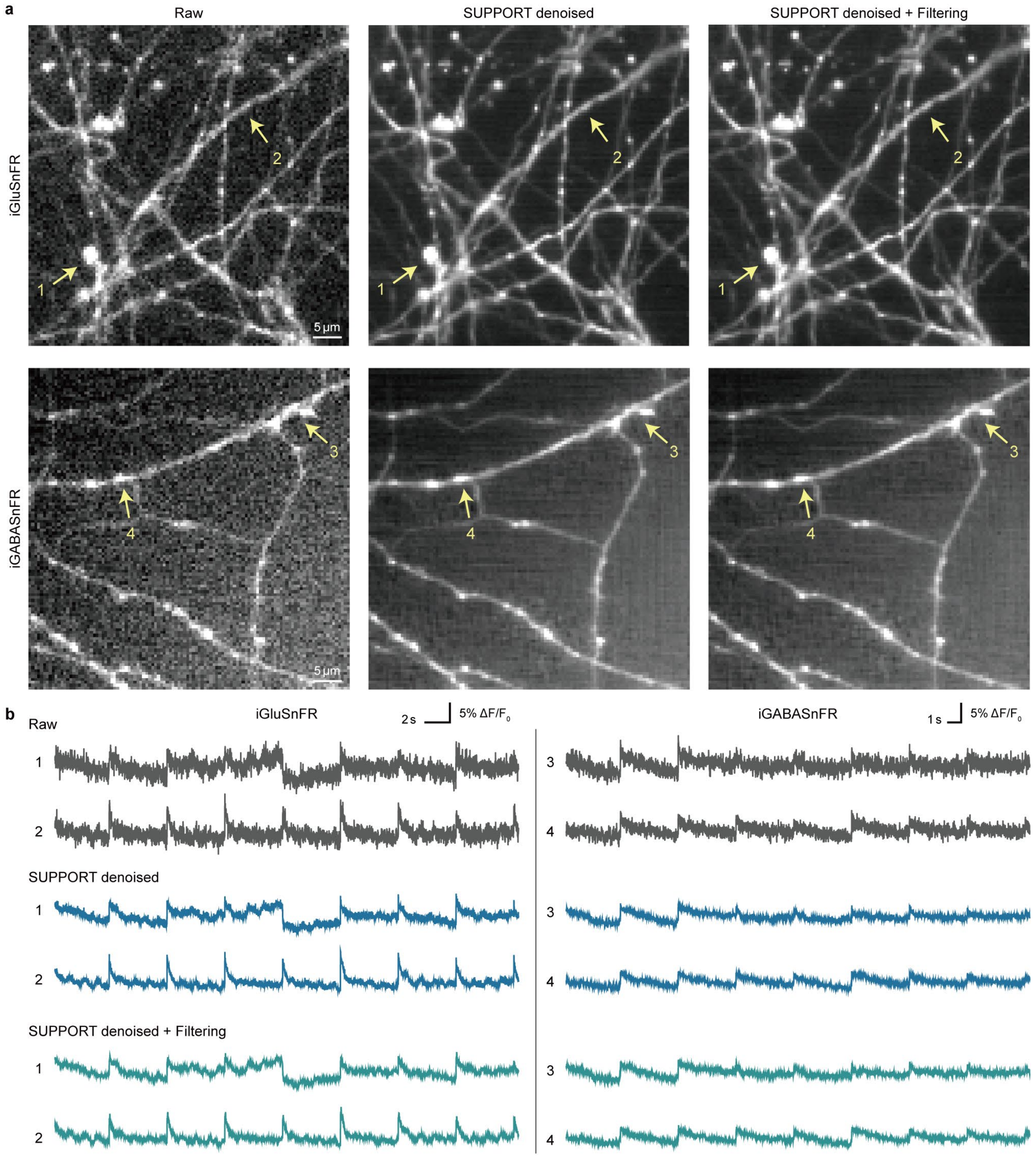

Supplementary Fig. 58: Applying SUPPORT to functional imaging data of cultured cells of rat hippocampal neurons expressing iGluSnFR and iGABASnFR. a, Primary cultures of rat hippocampal neurons expressing SF.iGluSnFR A184V and iGABASnFR F102G along with SynapsinI-mCherry marker. Representative frames of raw, SUPPORT-denoised, and SUPPORT-denoised followed by temporally filtered data. Four regions of interest (ROIs) are indicated by yellow arrows. **b**, Traces from raw, SUPPORT-denoised, and SUPPORT-denoised followed by filtered data extracted from four ROIs in **a**.

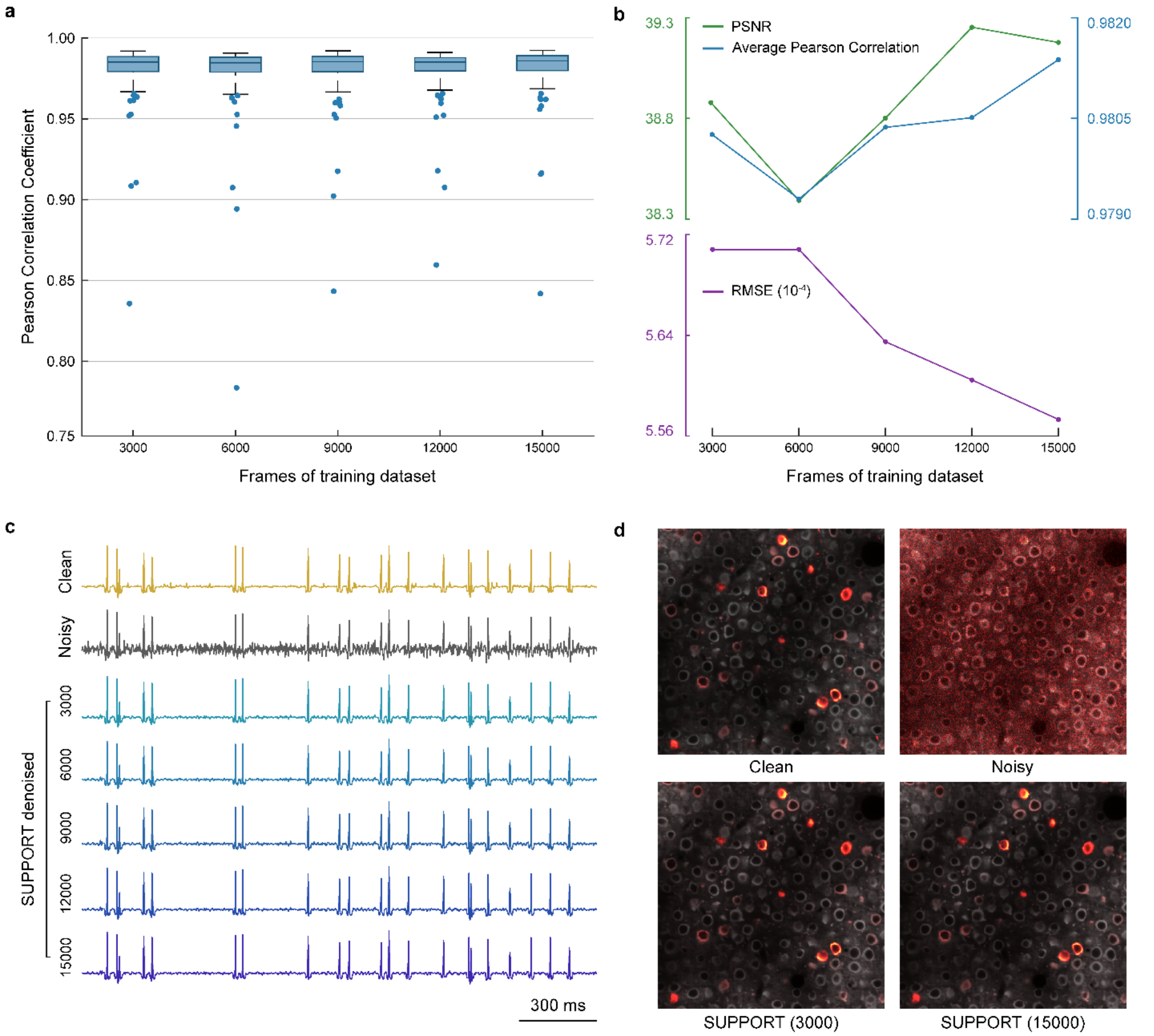

Supplementary Fig. 59: Denoising performance as a function of the size of training data. a, Pearson correlation coefficients between trace extracted from clean (ground truth) video and SUPPORT-denoised videos. Synthetic data with spike width of 3 ms were used for comparison. SUPPORT was trained with five different sizes of training datasets to evaluate the correlation between denoising performance and dataset size. N=116, which represents the number of neurons. **b**, PSNR, average Pearson correlation coefficients, and RMSE between clean video and SUPPORT-denoised videos. All three metrics show a trend of performance increases with larger training datasets. **c**, Traces extracted from a single cell of the clean video, SUPPORT-denoised videos, and the noisy video. While performance improves with larger datasets, traces show that SUPPORT was able to denoise with 3,000 frames of training data. **d**, Representative frames of clean video, noisy video, and SUPPORT-denoised videos after baseline correction. SUPPORT trained with 3,000 frames and 15,000 frames were used. Representative frames of clean and SUPPORT-denoised videos.

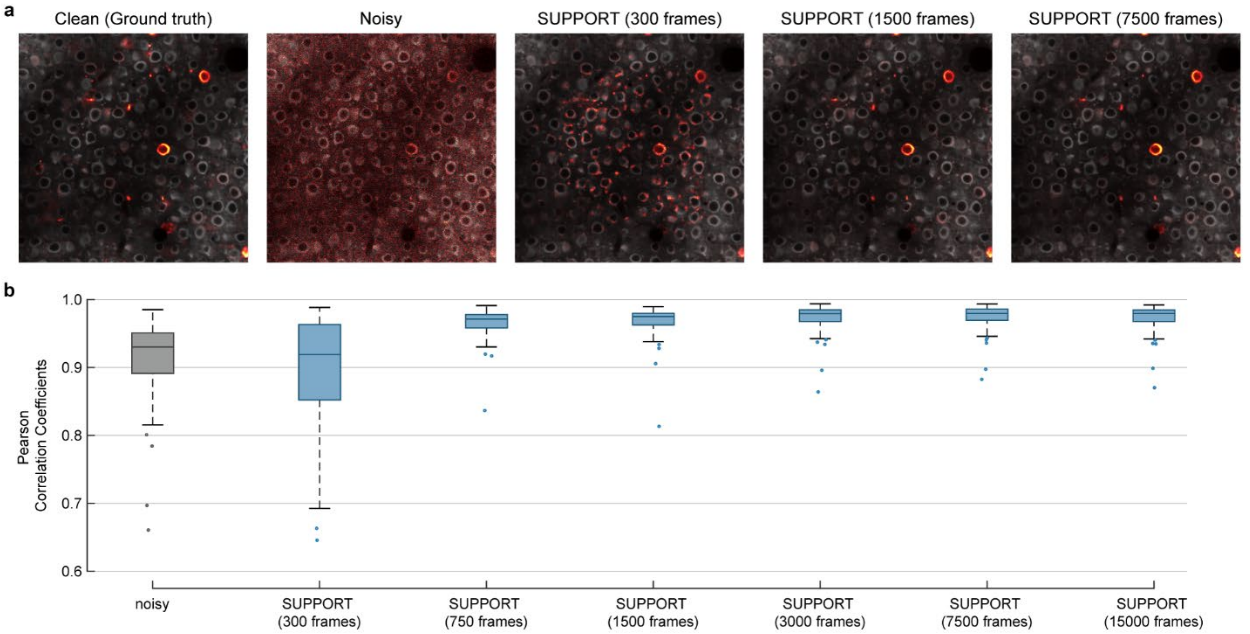

Supplementary Fig. 60: Extended denoising performance analysis as a function of the size of training data. a, Representative frames of clean video, noisy video, and SUPPORT-denoised videos after baseline correction. SUPPORT trained with 300, 1500, and 7500 frames were used. **b**, Box-and-whisker plot showing Pearson correlation coefficients before and after denoising data trained with different sizes of training data. N=116, which represents the number of neurons.

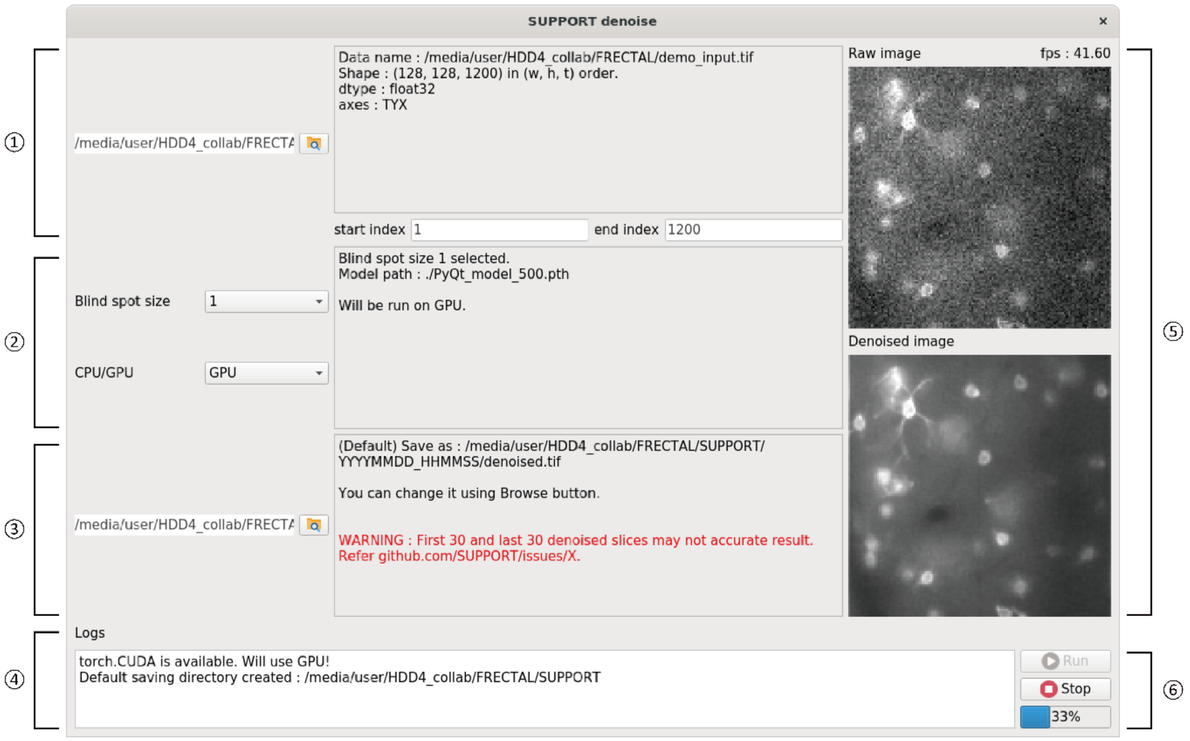

① (Input) Image path and information
② (Model) Blind spot size and CPU/GPU setting
③ (Save) Saving directory for denoised image and log files

④ Logs
⑤ Raw and denoised image in real time
⑥ Run / Stop button and progress bar

Supplementary Fig. 61: Python GUI: SUPPORT software with a graphical user interface. The user can choose the size of the blind spot and CPU/GPU option. Once the Run button is pressed, the software loads and denoises using a selected model, shows the denoised frame in real time, and saves denoised frames.

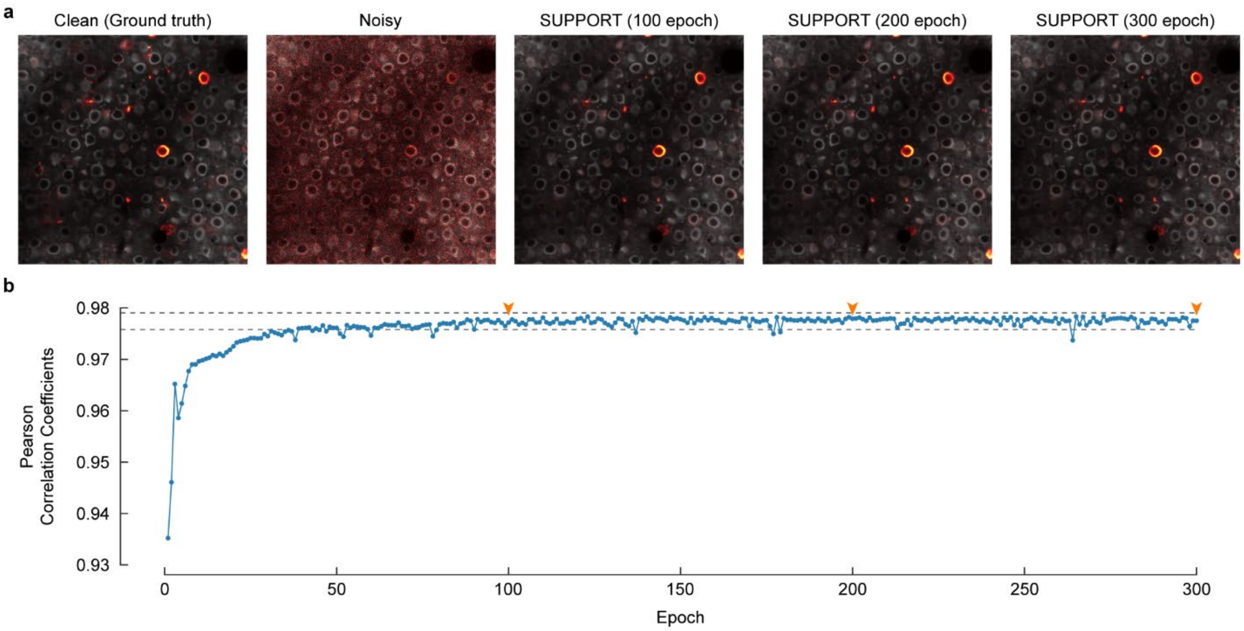

Supplementary Fig. 62: Denoising performance as a function of the iteration of training. a, Representative frames of clean video, noisy video, and SUPPORT-denoised videos after baseline correction. SUPPORT trained for 100, 200, and 300 epochs were used. **b**, Line plot showing Pearson correlation coefficients as a function of training iterations.

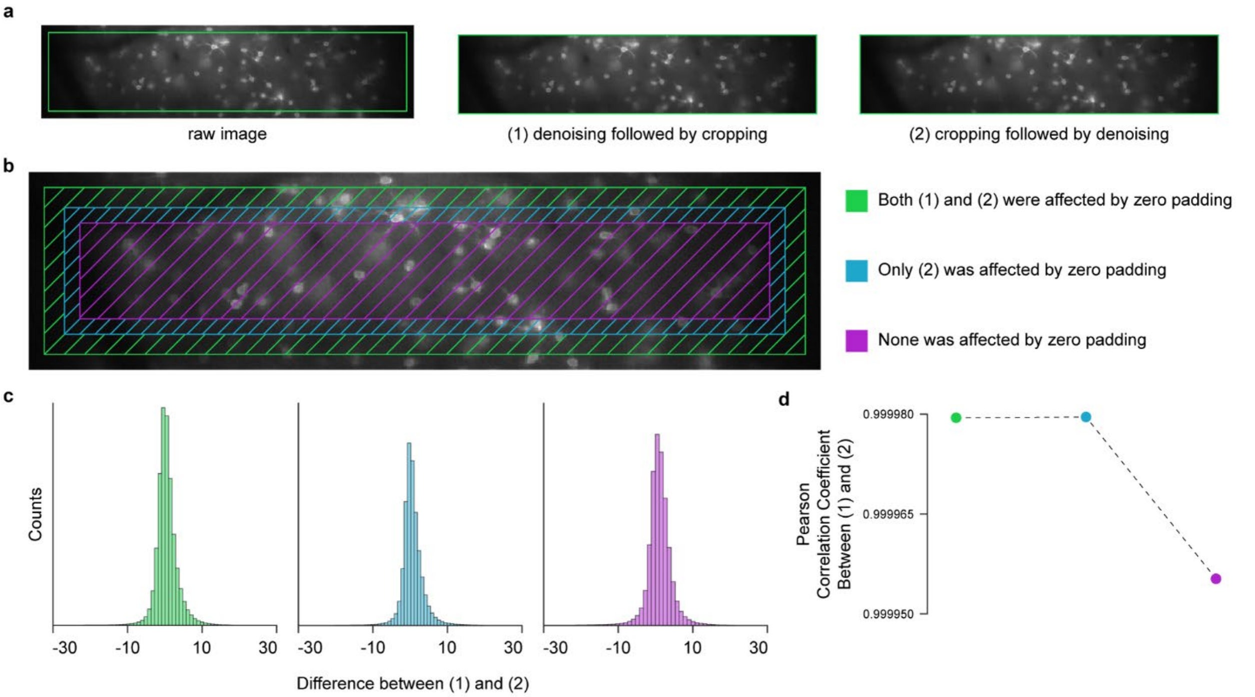

Supplementary Fig. 63: Analysis of the effect of padding at the edge of the images on SUPPORT denoising. **a**, Representative frames of raw video, (1) SUPPORT-denoised video followed by cropping, and (2) cropped video followed by SUPPORT-denoising. Dataset of in vivo mouse cortex layer 1 expressing Voltron1 was used. **b**, Regions, where both denoised videos are affected by zero padding, were colored green, and only one affected was colored blue, none were affected were colored purple. **c**, Histogram of pixel value differences between the two videos in each region described in **b**. **d**, Line plot showing Pearson correlation coefficient between the two videos for each region.

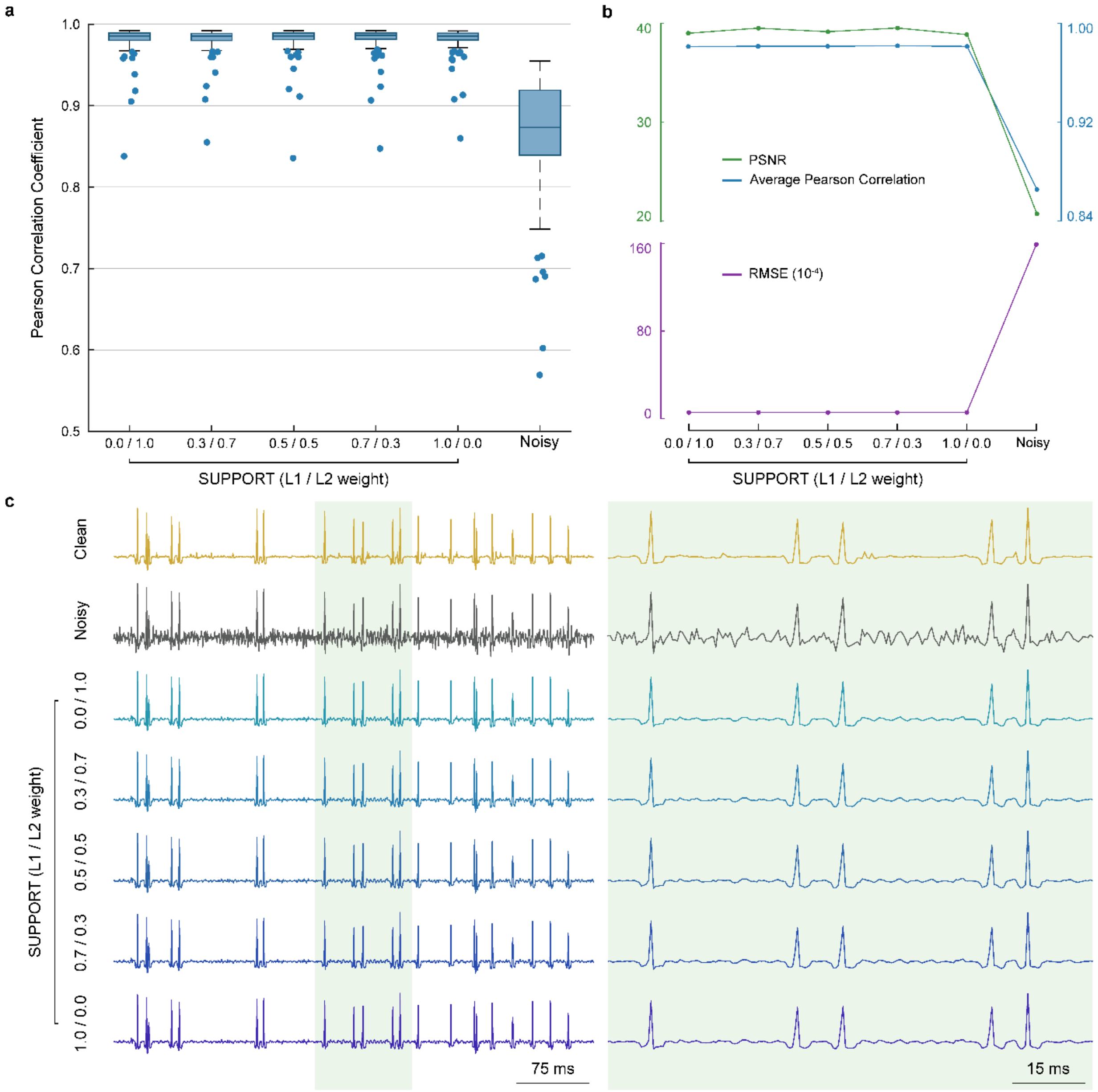

Supplementary Fig. 64: L1 and L2 loss comparison on simulation data. a, Pearson correlation coefficients between traces from clean (ground truth) video and SUPPORT-denoised video or noisy video. SUPPORT-denoised videos were acquired with five different training loss settings. N=116, which represents the number of neurons. **b**, PSNR, average Pearson correlation coefficients, and RMSE between clean video and SUPPORT-denoised videos or noisy video. All three metrics indicate that the weight of L1 and L2 loss for training SUPPORT did not significantly change denoising performance. **c**, Traces extracted from a single cell of the clean video, SUPPORT-denoised videos, and noisy video. Temporally expanded traces from the green area of the left are shown on the right. This again shows that the weight of L1 and L2 loss does not significantly change SUPPORT's output.

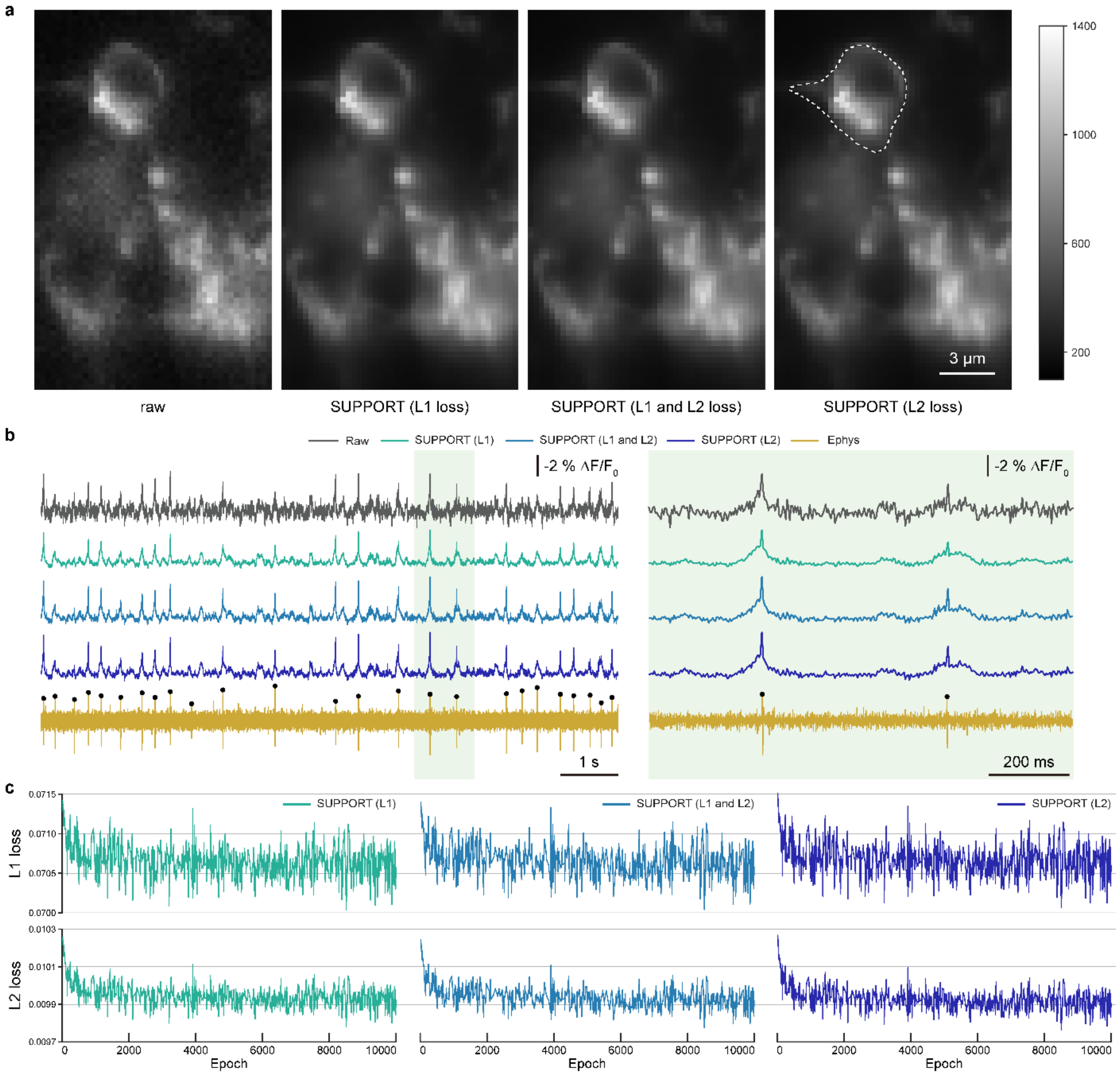

Supplementary Fig. 65: L1 and L2 loss comparison on voltage imaging data with simultaneous electrophysiological recording. a, From left to right: Representative frames from the raw data, SUPPORT trained using L1 loss function, both L1 and L2 loss functions, and L2 loss function. For the training setting that used both L1 and L2 loss, equal weights of L1 and L2 were used. The boundary of the region of interest (ROI) is drawn with a white dotted line. **b**, Traces extracted from the raw data, SUPPORT trained using L1 loss function, both L1 and L2 loss function, and L2 loss function for the ROI in **a**, and electrophysiological recording. Detected spikes are marked with black dots. Temporally expanded traces from the green area of the left are shown on the right. Using additional L2 loss showed better reconstruction of the spikes after SUPPORT denoising. **c**, L1 loss and L2 loss were tracked during the training procedure for SUPPORT using L1 loss function, both L1 and L2 loss functions, and L2 loss function.

SUPPLEMENTARY VIDEOS

Supplementary Video 1. Synthetic voltage imaging

Supplementary Video 2. Single-neuron simultaneous electrophysiology and voltage imaging of larval zebrafish

Supplementary Video 3. Synthetic voltage imaging data with propagating action potential

Supplementary Video 4. Population voltage imaging of larval zebrafish spinal cord

Supplementary Video 5. Volumetric time-lapse imaging of *C. elegans*

Supplementary Video 6. Volumetric structural imaging of mouse embryos recorded with expansion microscopy

Supplementary Video 7. Calcium imaging of larval zebrafish

Supplementary Video 8. Voltage imaging of cardiac cells

Supplementary Video 9. Multi-color in vivo imaging of various mouse organs

SUPPLEMENTARY TABLES

Region	Cell type	Recording rate (Hz)	Reporter	Imaging modality	Reference
**Voltage Imaging**					
Mouse cortex L1 (Fig. 1, 5, Supplementary Fig. 17, Supplementary Fig. 18, Supplementary Fig. 20, Supplementary Fig. 22, Supplementary Fig. 23, Supplementary Fig. 24, Supplementary Fig. 25, Supplementary Fig. 26, Supplementary Fig. 27, Supplementary Fig. 28, Supplementary Fig. 29, Supplementary Fig. 30, Supplementary Fig. 41, Supplementary Fig. 44, Supplementary Fig. 63)	interneurons (*Ndnf+*)	400	Voltron1	wide-field fluorescence	reporter and data: Abdelfattah, A. S. et al.[2]
Zebrafish dorsal part of the cerebellum (Fig. 3, Supplementary Fig. 9, Supplementary Fig. 10, Supplementary Fig. 65, Supplementary Video 2)	excitatory (*vGlut2a*)	300	Voltron1 + cell-attached extracellular recording	light sheet	reporter and data: Abdelfattah, A. S. et al.[2]
Zebrafish spinal cord (Fig. 5, Supplementary Fig. 19, Supplementary Fig. 20, Supplementary Fig. 37, Supplementary Fig. 41, Supplementary Video 3)	excitatory (*vGlut2a*)	1000	zArchon1	light sheet	reporter: Piatkevich, K. D. et al.[3]<br>data: Xie, M. E. et al.[4]

Mouse cortex L2/3 (Fig. 4, 6, Supplementary Fig. 12)	pyramidal cells	1000	QuasAr6a + patch clamp	one-photon epifluorescence microscopy with targeted illumination	reporter: Tian, H. et al.[5]<br>data: this work
Mouse cortex L2/3 (Supplementary Fig. 14, Supplementary Fig. 15)	pyramidal cells	1000	Voltron2 + patch clamp	one-photon epifluorescence microscopy with targeted illumination	reporter: Abdelfattah, A. S. et al.[6]<br>data: this work
Cultured rat hippocampal neuron (Supplementary Fig. 16)	primary neurons	1000	BeRST1	wide-field fluorescence	reporter: Huang, Y. L., Walker, A. S. & Miller, E. W.[7]<br>data: this work
Mouse hippocampus CA1 (Supplementary Fig. 18, Supplementary Fig. 31, Supplementary Fig. 32, Supplementary Fig. 33, Supplementary Fig. 34, Supplementary Fig. 35, Supplementary Fig. 36, Supplementary Fig. 42)	interneurons	1000	paQuasAr3-s	micromirror-based, soma-targeted, structured illumination	reporter and data: Adam, Y. et al.[8]
Zebrafish tegmental area (Supplementary Fig. 18, Supplementary Fig. 38, Supplementary Fig. 39, Supplementary Fig. 40, Supplementary Fig. 43)	excitatory (*vGlut2a*)	300	Voltron1	light sheet	reporter and data: Abdelfattah, A. S. et al.[2]
Mouse cortex L1 (Fig. 4)	Interneurons (Ndnf+)	400	Voltron1 + patch clamp	wide-field fluorescence	reporter: Abdelfattah, A. S. et al.[2]<br>data: Rozsa, Marton et al.[9]
Mouse culture (Supplementary Fig. 21)	primary neurons	500	SomArchon	DMD-targeted illumination	reporter: Piatkevich, K.D. et al.[10]<br>data: Xiao, S. et al.[11]

Mouse hippocampus (Fig. 6)	N/A	800	SomArchon	wide-field fluorescence	reporter and data: Piatkevich, K.D. et al.[10]
**Structural Imaging**					
C. elegans (Extended data Fig. 1, Supplementary Fig. 48, Supplementary Video 4)	pan-neuronal (*H20*)	4.75	mCherry	confocal	reporter: Shaner, N. C. et al.[10]<br>data: Toyoshima, Y. et al.[12]
Penicillium (Fig. 6)	entire	Static	N/A	confocal	data: this work
Mouse embryos; intestine, bone, tail (Extended data Fig. 2, Supplementary Fig. 50)	entire	Static	Alexa Fluor 488 NHS-ester	confocal	data: Sim, J. et al.[13]
**Calcium Imaging**					
Mouse cortex L2/3 (Supplementary Fig. 51, Supplementary Fig. 52)	pyramidal cells	122	jGCaMP8f + loose-seal cell attached electrophysiological recording	two-photon	reporter: Zhang, Y. et al.[14]<br>data: Rozsa, M. et al.[15]
Mouse cortex V1 (Supplementary Fig. 53)	excitatory (*syn*)	60	GCaMP6f + loose-seal cell attached electrophysiological recording	two-photon	reporter: Chen, T. W. et al.[16]<br>data: GENIE project[17]
Zebrafish (Supplementary Fig. 54, Supplementary Fig. 55, Supplementary Video 5)	pan-neuronal (*huc*)	1, 2, 4	GCaMP7a	confocal	reporter: Muto, A., Ohkura, M., Abe, G., Nakai, J. & Kawakami, K.[18]<br>data: this work
Mouse cortex V1 (Supplementary Fig. 56)	N/A	7.5	GCaMP6s	two-photon	reporter: Chen, T. W. et al.[16]<br>data: Neurofinder[19]
C. elegans (Supplementary Fig. 49)	pan-neuronal (*H20*)	4.75	YC2.6	confocal	reporter: Shaner, N. C. et al.[20]<br>data: Toyoshima, Y. et al.[12]

Others					
Mouse ear skin, kidney, muscle (Supplementary Fig. 57)	N/A	15	H2B-GFP, mTmG	confocal	reporter: Kanda, T., Sullivan, K.F., & Wahl, G.M.[21], Muzumdar, M.D., Tasic, B., Miyamichi, K., Li, L., & Luo, L.[22] data: Hwang, Y. et al.[49,50,51,52,53,54,55,56,31]
Rat hippocampus culture (Supplementary Fig. 58)	primary neuron	100	iGluSnFR, iGABASnFR	wide-field fluorescence	reporter: Marvin, J.S. et al.[32] data: this work

Supplementary Table 1. List of datasets analyzed in this study.

Dataset	Network configuration
**Voltage imaging**	
Mouse cortex L1 (Fig. 1, 5, Supplementary Fig. 17, Supplementary Fig. 18, Supplementary Fig. 20, Supplementary Fig. 22, Supplementary Fig. 23, Supplementary Fig. 24, Supplementary Fig. 25, Supplementary Fig. 26, Supplementary Fig. 27, Supplementary Fig. 28, Supplementary Fig. 29, Supplementary Fig. 30, Supplementary Fig. 41, Supplementary Fig. 44, Supplementary Fig. 63)	Default (input frame = 61, blind conv channels = 64, one by one channels = [32, 16], last layer channels = [64, 32, 16], Unet channels = [16, 32, 64, 128, 256], blind spot size = [1, 1])
Zebrafish dorsal part of the cerebellum (Fig. 3, Supplementary Fig. 9, Supplementary Fig. 10, Supplementary Fig. 65, Supplementary Video 2)	Changed blind spot size to [3, 3] from default
Zebrafish spinal cord (Fig. 5, Supplementary Fig. 19, Supplementary Fig. 20, Supplementary Fig. 37, Supplementary Fig. 41, Supplementary Video 3)	Default
Mouse cortex L2/3 (Fig. 4, 6, Supplementary Fig. 12)	Default
Mouse cortex L2/3 (Supplementary Fig. 14, Supplementary Fig. 15)	Default
Cultured rat hippocampal neuron (Supplementary Fig. 16)	Default
Mouse hippocampus CA1 (Supplementary Fig. 18, Supplementary Fig. 31, Supplementary Fig. 32, Supplementary Fig. 33, Supplementary Fig. 34, Supplementary Fig. 35, Supplementary Fig. 36, Supplementary Fig. 42)	Changed blind spot size to [3, 3] from default
Zebrafish tegmental area (Supplementary Fig. 18, Supplementary Fig. 38, Supplementary Fig. 39, Supplementary Fig. 40, Supplementary Fig. 43)	Changed blind spot size to [3, 3] from default
Mouse cortex L1 (Fig. 4)	Changed blind spot size to [3, 3] from default
Mouse culture (Supplementary Fig. 21)	Changed blind spot size to [3, 3] from default
Mouse hippocampus (Fig. 6)	Changed Unet channels to [64, 128, 256, 512, 1024] from default
**Structural Imaging**	
C. elegans (Extended data Fig. 1, Supplementary Fig. 48, Supplementary Video 4)	Changed input frame to 21 from default, Changed blind spot size to [3, 3] from default
Penicillium (Fig. 6)	Changed input frame to 21 from default
Mouse embryos; intestine, bone, tail (Extended data Fig. 2, Supplementary Fig. 50)	Changed input frame to 21 from default
**Calcium Imaging**	
Mouse cortex L2/3 (Supplementary Fig. 51, Supplementary Fig. 52)	Changed blind spot size to [3, 3] from default
Mouse cortex V1 (Supplementary Fig. 53)	Default
Zebrafish (Supplementary Fig. 54, Supplementary Fig. 55, Supplementary Video 5)	Default
Mouse cortex V1 (Supplementary Fig. 56)	Changed blind spot size to [3, 3] from default

C. elegans (Supplementary Fig. 49)	Changed blind spot size to [3, 3] from default
**Others**	
Mouse ear skin, kidney, muscle (Supplementary Fig. 57)	Changed blind spot size to [1, 19] from default
Rat hippocampus culture (Supplementary Fig. 58)	Default

Supplementary Table 2. List of network configurations used for each dataset.

REFERENCES

1. Honzátko, D., Bigdeli, S. A., Türetken, E. & Dunbar, L. A. Efficient Blind-Spot Neural Network Architecture for Image Denoising. In *2020 7th Swiss Conference on Data Science (SDS)* (2020).

2. Abdelfattah, A. S. et al. Bright and photostable chemigenetic indicators for extended in vivo voltage imaging. *Science* **365**, 699-704 (2019).

3. Piatkevich, K. D. et al. A robotic multidimensional directed evolution approach applied to fluorescent voltage reporters. *Nat. Chem. Biol.* **14**, 352-360 (2018).

4. Xie, M. E. et al. High-fidelity estimates of spikes and subthreshold waveforms from 1-photon voltage imaging in vivo. *Cell Rep*. **35**, 108954 (2021).

5. Tian, H. et al. All-optical electrophysiology with improved genetically encoded voltage indicators reveals interneuron network dynamics in vivo. Preprint at *bioRxiv* https://doi.org/10.1101/2021.11.22.469481 (2021).

6. Abdelfattah, A. S. et al. Sensitivity optimization of a rhodopsin-based fluorescent voltage indicator. Preprint at *bioRxiv* https://doi.org/10.1101/2021.11.09.467909 (2021).

7. Huang, Y. L., Walker, A. S. & Miller, E. W. A photostable silicon rhodamine platform for optical voltage sensing. *J. Am. Chem. Soc.* **137**, 10767-10776 (2015).

8. Adam, Y. et al. Voltage imaging and optogenetics reveal behaviour-dependent changes in hippocampal dynamics. *Nature* **569**, 413-417 (2019).

9. Rozsa, Marton; Singh, Amrita; Svoboda, Karel (2021): Simultaneous Voltron (1.0) imaging and whole-cell patch-clamp recordings of somatosensory cortex layer 1 interneurons in vivo. Janelia Research Campus. Collection. https://doi.org/10.25378/janelia.c.5325254.v1

10. Piatkevich, K.D. et al. Population imaging of neural activity in awake behaving mice. *Nature* **574**, 413-417 (2019).

11. Xiao, S. et al. Large-scale voltage imaging in behaving mice using targeted illumination. *iScience* **24**, 103263 (2021).

12. Toyoshima, Y. et al. Accurate automatic detection of densely distributed cell nuclei in 3D space. *PLoS Comput. Biol.* **12**, e1004970 (2016).

13. Sim, J. et al. Nanoscale resolution imaging of the whole mouse embryos and larval zebrafish using expansion microscopy. Preprint at *bioRxiv* https://doi.org/10.1101/2021.05.18.443629 (2022).

14. Zhang, Y. et al. Fast and sensitive GCaMP calcium indicators for imaging neural populations. Preprint at *bioRxiv* https://doi.org/10.1101/2021.11.08.467793 (2021).

15. Rózsa, M. et al. Simultaneous loose seal cell-attached recordings and two-photon imaging of GCaMP8 expressing mouse V1 neurons with drifting gratings visual stimuli. DANDI archive https://doi.org/10.5281/zenodo.7041535 (2022)

16. Chen, T. W. et al. Ultrasensitive fluorescent proteins for imaging neuronal activity. *Nature* **499**, 295-300 (2013).

17. GENIE project. Simultaneous imaging and loose-seal cell-attached electrical recordings from neurons expressing a variety of genetically encoded calcium indicators. CRCNS.org https://doi.org/10.6080/K02R3PMN (2015).

18. Muto, A., Ohkura, M., Abe, G., Nakai, J. & Kawakami, K. Real-time visualization of neuronal activity during perception. *Curr. Biol.* **23**, 307-311 (2013).

19. CodeNeuro: neurofinder public benchmark (2016). http://neurofinder.codeneuro.org/

20. Shaner, N. C. et al. Improved monomeric red, orange and yellow fluorescent proteins derived from Discosoma sp. red fluorescent protein. *Nat. Biotechnol.* **22**, 1567-1572 (2004).

21. Kanda, T., Sullivan, K.F., & Wahl, G.M. Histone–GFP fusion protein enables sensitive analysis of chromosome dynamics in living mammalian cells. *Curr. Biol.* **8**, 377-385 (1998).

22. Muzumdar, M.D., Tasic, B., Miyamichi, K., Li, L., & Luo, L. A global double-fluorescent Cre reporter mouse. *Genesis*, **45**, 593-605 (2007).

23. Hwang, Y. et al. In vivo cellular-level real-time pharmacokinetic imaging of free-form and liposomal indocyanine green in liver. *Biomed. Opt. Express* **8**, 4706-4716 (2017).

24. Kim, S.B. et al. Caspase-8 controls the secretion of inflammatory lysyl-tRNA synthetase in exosomes from cancer cells. *JCB* **216**, 2201-2216 (2017).

25. Kim, J.Y. et al. Nanoparticle-assisted transcutaneous delivery of a signal transducer and activator of transcription 3-inhibiting peptide ameliorates psoriasis-like skin inflammation. *ACS nano* **12**, 6904-6916 (2018).

26. Choi, J. et al. In vivo longitudinal 920 nm two-photon intravital kidney imaging of a dynamic 2, 8-DHA crystal formation and tubular deterioration in the adenine-induced chronic kidney disease mouse model. *Biomed. Opt. Express* **14**, 1647-1658 (2023).

27. Lee, E.M. et al. Effect of resveratrol treatment on graft revascularization after islet transplantation in streptozotocin-induced diabetic mice. *Islets* **10**, 25-39 (2018).

28. Moon, J. et al. Intravital two-photon imaging and quantification of hepatic steatosis and fibrosis in a live small animal model. *Biomed. Opt. Express* **12**, 7918-7927 (2021).

29. Moon, J. & Kim, P. Intravital two-photon imaging of dynamic alteration of hepatic lipid droplets in fasted and refed state. *JLA* **10**, 313 (2021).

30. Moon, J. et al. Intravital longitudinal imaging of hepatic lipid droplet accumulation in a murine model for nonalcoholic fatty liver disease. *Biomed. Opt. Express* **11**, 5132-5146 (2020).

31. Seo, H., Hwang, Y., Choe, K. & Kim, P. In vivo quantitation of injected circulating tumor cells from great saphenous vein based on video-rate confocal microscopy. *Biomed. Opt. Express* **6**, 2158-2167 (2015).

32. Marvin, J.S. et al. Stability, affinity, and chromatic variants of the glutamate sensor iGluSnFR. *Nat. Methods* **15**, 936-939 (2018).
