# Supplementary material for: Statistically unbiased prediction enables accurate denoising of voltage imaging data
Source: Nat Methods. 2023 Sep 18;20(10):1581–92. doi: 10.1038/s41592-023-02005-8 (PMC10555843; doi:10.1038/s41592-023-02005-8)
Supplement: Supplementary file 2 — Reporting Summary [file 41592_2023_2005_MOESM2_ESM.pdf]

nature portfolio

Corresponding author(s): Young-Gyu Yoon

Last updated by author(s): Jul 28, 2023

# Reporting Summary

Nature Portfolio wishes to improve the reproducibility of the work that we publish. This form provides structure for consistency and transparency in reporting. For further information on Nature Portfolio policies, see our Editorial Policies and the Editorial Policy Checklist.

## Statistics

For all statistical analyses, confirm that the following items are present in the figure legend, table legend, main text, or Methods section.

| n/a | Confirmed | |
|---|---|---|
| ☐ | ☒ | The exact sample size (*n*) for each experimental group/condition, given as a discrete number and unit of measurement |
| ☒ | ☐ | A statement on whether measurements were taken from distinct samples or whether the same sample was measured repeatedly |
| ☐ | ☒ | The statistical test(s) used AND whether they are one- or two-sided<br>*Only common tests should be described solely by name; describe more complex techniques in the Methods section.* |
| ☒ | ☐ | A description of all covariates tested |
| ☒ | ☐ | A description of any assumptions or corrections, such as tests of normality and adjustment for multiple comparisons |
| ☒ | ☐ | A full description of the statistical parameters including central tendency (e.g. means) or other basic estimates (e.g. regression coefficient) AND variation (e.g. standard deviation) or associated estimates of uncertainty (e.g. confidence intervals) |
| ☐ | ☒ | For null hypothesis testing, the test statistic (e.g. $F$, $t$, $r$) with confidence intervals, effect sizes, degrees of freedom and $P$ value noted<br>*Give P values as exact values whenever suitable.* |
| ☒ | ☐ | For Bayesian analysis, information on the choice of priors and Markov chain Monte Carlo settings |
| ☒ | ☐ | For hierarchical and complex designs, identification of the appropriate level for tests and full reporting of outcomes |
| ☐ | ☒ | Estimates of effect sizes (e.g. Cohen's *d*, Pearson's *r*), indicating how they were calculated |

*Our web collection on statistics for biologists contains articles on many of the points above.*

## Software and code

Policy information about availability of computer code

| | |
|---|---|
| Data collection | Fusion v2.1.0.34 (for Andor Dragonfly spinning disk confocal), NIS-Elements AR v5.11.01 (for Nikon C2 plus) |
| Data analysis | ImageJ/Fiji 1.53t, MATLAB R2022b v9.13.0, Python v3.9, MetaMorph (64-bit) Version 7.10.3.279 |

For manuscripts utilizing custom algorithms or software that are central to the research but not yet described in published literature, software must be made available to editors and reviewers. We strongly encourage code deposition in a community repository (e.g. GitHub). See the Nature Portfolio guidelines for submitting code & software for further information.

## Data

Policy information about availability of data

All manuscripts must include a data availability statement. This statement should provide the following information, where applicable:

- Accession codes, unique identifiers, or web links for publicly available datasets
- A description of any restrictions on data availability
- For clinical datasets or third party data, please ensure that the statement adheres to our policy

The dataset of one-photon epifluorescence imaging with targeted illumination of QuasAr6a expressing mouse cortex L2/3 neurons simultaneously recorded with patch clamp can be downloaded from (https://zenodo.org/record/8176722).
The dataset of one-photon epifluorescence imaging with targeted illumination of Voltron2 expressing mouse cortex L2/3 neurons simultaneously recorded with patch clamp can be downloaded from (https://zenodo.org/record/8176722).

The dataset of wide-field fluorescence imaging of SomArchon expressing mouse hippocampus neurons can be downloaded from (https://zenodo.org/record/8176722).
The dataset of confocal imaging of volumetric structural imaging of Penicillium can be downloaded from (https://zenodo.org/record/8176722).
The dataset of confocal imaging of volumetric structural imaging of Alexa fluor 488 NHS-ester stained mouse embryos can be downloaded from (https://zenodo.org/record/8176722).
The dataset of in vivo single neuron simultaneous calcium recording of jGCaMP8f and electrophysiology can be downloaded from the DANDI (https://dandiarchive.org/dandiset/000168?search=jgcamp8m&pos=1).
The dataset of confocal imaging of GCaMP7a expressing zebrafish neurons can be downloaded from (https://zenodo.org/record/8176722).
The datasets from previous publications are publicly available, and the corresponding links can be found in each respective publication.

## Human research participants

Policy information about studies involving human research participants and Sex and Gender in Research.

| | |
|---|---|
| Reporting on sex and gender | N/A |
| Population characteristics | N/A |
| Recruitment | N/A |
| Ethics oversight | N/A |

Note that full information on the approval of the study protocol must also be provided in the manuscript.

# Field-specific reporting

Please select the one below that is the best fit for your research. If you are not sure, read the appropriate sections before making your selection.

☒ Life sciences ☐ Behavioural & social sciences ☐ Ecological, evolutionary & environmental sciences

For a reference copy of the document with all sections, see nature.com/documents/nr-reporting-summary-flat.pdf

# Life sciences study design

All studies must disclose on these points even when the disclosure is negative.

| | |
|---|---|
| Sample size | Each dataset presented in the manuscript corresponds to one sample. To validate our method, we used multiple datasets (Voltron1(n=9), Voltron2(n=7), paQuasAr3-s(n=12), QuasAr6a(n=6), zArchon1(n=1), SomArchon(n=1), and BeRST1(n=1)). |
| Data exclusions | None of data were excluded. |
| Replication | The method demonstrated in this work was applied on various samples and fluorescence indicators (Voltron1(n=9), Voltron2(n=7), paQuasAr3-s(n=12), QuasAr6a(n=6), zArchon1(n=1), SomArchon(n=1), and BeRST1(n=1)) and yielded consistent results. |
| Randomization | We used randomly selected n samples among m acquired images for training (m >= n). |
| Blinding | Not applicable since the training set and test set are identical for this study. |

# Reporting for specific materials, systems and methods

We require information from authors about some types of materials, experimental systems and methods used in many studies. Here, indicate whether each material, system or method listed is relevant to your study. If you are not sure if a list item applies to your research, read the appropriate section before selecting a response.

## Materials & experimental systems

| n/a | Involved in the study | |
|---|---|---|
| ☒ | ☐ | Antibodies |
| ☒ | ☐ | Eukaryotic cell lines |
| ☒ | ☐ | Palaeontology and archaeology |
| ☐ | ☒ | Animals and other organisms |
| ☒ | ☐ | Clinical data |
| ☒ | ☐ | Dual use research of concern |

## Methods

| n/a | Involved in the study | |
|---|---|---|
| ☒ | ☐ | ChIP-seq |
| ☒ | ☐ | Flow cytometry |
| ☒ | ☐ | MRI-based neuroimaging |

## Animals and other research organisms

Policy information about studies involving animals; ARRIVE guidelines recommended for reporting animal research, and Sex and Gender in Research

| | |
|---|---|
| Laboratory animals | CD-1 mice of 3-4 weeks were used for simultaneous patch clamp and voltage imaging in brain slices. NDNF-Cre+/- (JAX #028536) or PV-Cre+/- (JAX #017320) of 6 weeks - 8 months were used for in vivo QuasAr6 voltage imaging. Mice were housed in standard conditions with a reverse 12-h light/dark cycle at 23°C and 40-60% humidity. Up to five mice were housed per cage after weaning, with water and food provided ad libitum.<br><br>Mouse embryos isolated on day 15.5 of pregnancy in C57BL/6J mice were used for volumetric structural imaging using expansion microscopy.<br><br>Rat embryos isolated on day 18 of pregnancy were used for imaging spontaneous neurotransmission.<br><br>Transgenic zebrafish larvae (Tg(huc:GAL4);Tg(UAS:GCaMP7a)) with Casper background at 3-4dpf were used for calcium imaging experiments. Zebrafish were maintained under standard conditions at 28°C and a 14:10 hour light:dark cycle.<br><br>H2B-GFP (Jackson Laboratory, Stock No. 006069) and mTmG (Jackson Laboratory, Stock No. 007676) mice were purchased from the Jackson Laboratory (Bar Harbor, USA). The mice were housed in cages with independent ventilation, controlled temperature (22.5 ° C), and humidity (52.5%). They were provided ad libitum access to a standard diet and water under a 12/12 hours light/dark cycle.<br><br>Mice for calcium imaging were cared for in compliance with the Guide for the Care and Use of Laboratory Animals. All experiments were approved by the Janelia Research Campus IACUC and IBC committees. Mice were housed on a free-standing, individually ventilated (approximately 60 air changes hourly) rack (Allentown). The holding room was ventilated with 100% outside filtered air with 15–20 air changes hourly. Each ventilated cage (Allentown) was provided with corncob bedding (Shepard Specialty Papers), at least 8g of nesting material (Bed-r'Nest, The Andersons) and red Mouse Tunnel (Bio-Serv). Mice were maintained on a 12:12-h light:dark cycle. The holding room temperature was maintained at 68–72°F with a relative humidity of 30–70%. Irradiated rodent laboratory chow (LabDiet 5053) was provided ad libitum. |
| Wild animals | Not involved in this study. |
| Reporting on sex | (mice) Mice of both sexes were used without regard to sex in this study. (zebrafish larvae, mouse embryos, rat embryos) Sex is not specified at this developmental stage and was therefore not determined. |
| Field-collected samples | Not involved in this study. |
| Ethics oversight | All experimental methods involving mice and zebrafish were approved by the Korea Advanced Institute of Science and Technology Institutional Animal Care and Use Committee (KAIST-IACUC).<br><br>All of the animal experiments were performed according to the Institute of Animal Care and Use Committee guidelines of Seoul National University (Seoul, Korea).<br><br>All procedures involving animals were in accordance with the National Institutes of Health guide for the care and use of laboratory animals and were approved by the Institutional Animal Care and Use Committee at Harvard University (Harvard-IACUC).<br><br>All surgical and experimental procedures were conducted in accordance with protocols approved by the Institutional Animal Care and Use Committee (IACUC) and Institutional Biosafety Committee (IBC) of Janelia Research Campus. |

Note that full information on the approval of the study protocol must also be provided in the manuscript.
